# Australian State and Territory Eclectic Approaches to Obesity Prevention in the Early Years: Policy Mapping and Perspectives of Senior Health Officials

**DOI:** 10.3389/fpubh.2022.781801

**Published:** 2022-06-03

**Authors:** Emma K. Esdaile, James Gillespie, Louise A. Baur, Li Ming Wen, Chris Rissel

**Affiliations:** ^1^Sydney School of Public Health, Faculty of Medicine and Health, The University of Sydney, Sydney, NSW, Australia; ^2^Prevention Research Collaboration, Faculty of Health and Medicine, Charles Perkins Centre, School of Public Health, The University of Sydney, Camperdown, NSW, Australia; ^3^The Centre for Research Excellence in the Early Prevention of Obesity in Childhood, Sydney, NSW, Australia; ^4^Menzies Centre for Health Policy, Charles Perkins Centre, The University of Sydney, Sydney, NSW, Australia; ^5^Specialty of Child and Adolescent Health, The University of Sydney, Sydney, NSW, Australia

**Keywords:** early childhood, policy, obesity prevention, systems-thinking, qualitative, eclecticism

## Abstract

**Background:**

The international increase in the prevalence of childhood obesity has hastened in recent decades. This rise has coincided with the emergence of comorbidities in childhood—such as type II diabetes, non-alcoholic fatty liver disease, metabolic syndrome, sleep apnoea and hypertension—formerly only described in adulthood. This phenomenon suggests global social and economic trends are impacting on health supportive environments. Obesity prevention is complex and necessitates both long-term and systems approaches. Such an approach considers the determinants of health and how they interrelate to one another. Investment in the early years (from conception to about 5 years of age) is a key life stage to prevent obesity and establish lifelong healthy habits relating to nutrition, physical activity, sedentary behavior and sleep. In Australia, obesity prevention efforts are spread across national and state/territory health departments. It is not known from the literature how, with limited national oversight, state and territory health departments approach obesity prevention in the early years.

**Methods:**

We conducted a qualitative study including policy mapping and interviews with senior officials from each Australian state/territory health department. A series of questions were developed from the literature to guide the policy mapping, drawing on the *World Health Organisation Ending Childhood Obesity Report*, and adapted to the state/territory context. The policy mapping was iterative. Prior to the interviews initial policy mapping was undertaken. During the interviews, these policies were discussed, and participants were asked to supply any additional policies of relevance to obesity prevention. The semi-structured interviews explored the approaches to obesity prevention taken in each jurisdiction and the barriers and enablers faced for policy implementation. Thematic analysis was used to analyse the data, using NVivo software.

**Results:**

State and territory approaches to obesity prevention are eclectic and while there are numerous similarities between jurisdictions, no two states are the same. The diversity of approaches between jurisdictions is influenced by the policy culture and unique social, geographic, and funding contexts in each jurisdiction. No Australian state/territory had policies against all the guiding questions. However, there are opportunities for sharing and collaborating within and between Australian jurisdictions to establish what works, where, and for whom, across Australia's complex policy landscape.

**Conclusions:**

Even within a single country, obesity prevention policy needs to be adaptable to local contexts. Opportunities for jurisdictions within and between countries to share, learn, and adapt their experiences should be supported and sustained funding provided.

## Introduction

The international increase in the prevalence of childhood obesity has coincided with the emergence of comorbidities formerly only described in adulthood—such as type II diabetes, fatty liver disease, metabolic syndrome, sleep apnoea and hypertension (1, 2). The first 2,000 days (from conception to about 5 years of age) is a key life stage to establish lifelong behaviors for health and to prevent obesity (3, 4).

In Australia, the issue of childhood obesity emerged as a distinct policy agenda in the early 2000s. Over the last two decades obesity has risen and fallen from national and subnational political agendas. Federalism shapes the ability to take policy initiatives. The six states (New South Wales (NSW), Victoria, Queensland, South Australia (SA), Western Australia (WA) and Tasmania) and two territories [Australian Capital Territory (ACT) and Northern Territory (NT)] are constrained by “vertical fiscal inequality”—the disproportion between Commonwealth dominance of tax revenues and the high spending responsibilities of the states. The Commonwealth uses its fiscal dominance to set conditions on expenditure in national funding agreements, e.g., the National Housing and Homelessness Agreement. State and territory governments have limited resources to fill funding gaps. Commonwealth fiscal decisions can greatly influence the social determinants of health, including the social safety net (e.g., welfare payments and conditions), housing policy and funding, out of pocket costs for primary health care, and industrial relations policy such as workforce casualisation and minimum wage (5). In 2008, the National Partnership Agreement on Preventive Health (NPAPH) was Australia's largest national investment in prevention and included a national Healthy Children's Initiative which focused on childhood obesity. Since that national funding was cut prematurely in 2014, subnational governments have independently pursued childhood obesity prevention initiatives.

The Early Prevention of Obesity in Childhood (EPOCH) Collaboration sought to answer if interventions in early life could prevent obesity across a range of modalities in real world intervention settings (6). The cohort included more than 2,300 first-time mothers in Australia and New Zealand. These interventions commenced in pregnancy or by 6 months of age and all ended by 2 years of age. They focused on knowledge, skills and self-efficacy for parents (usually mothers, although not exclusively) in relation to breastfeeding, transition to solids, the importance of “tummy time,” avoidance of screen time, and sleep (6). The EPOCH trials resulted in improved behaviors and small but significant improvements in child body mass index compared to controls at 18–24 months follow-up (6). Internationally, there is a paucity of programs to support parents in the latter half of the first 2,000 days (2–5 years) (7).

As some form of childcare is attended by approximately two thirds of children aged 1–4 years in Australia (8), early childhood education and care (ECEC) services are considered a key community setting for health promotion interventions for obesity prevention and establishing healthy lifestyle behaviors in the early years. A recent study among mothers of young children in NSW identified strong support for these interventions in ECEC settings (9). State and territory education departments (and the communities department in WA) have tasked Authorised Officers to enter and assess ECEC services against regulatory obligations and standards set by the national authority for the ECEC sector, the Australian Children's Education & Care Authority (ACECQA). State authorities and their Authorised Officers are given little guidance on how to support services to maximize the health and well-being for children attending care (10, 11). Nor has there been extensive engagement with the sector to identify how (or if) health promotion could be part of their core business.

Despite the positive intervention effects found in the EPOCH trials, difference between intervention and control groups had disappeared at follow-up at 3.5 and 5 years of age (12). This suggests that families need ongoing intervention to overcome the obesogenic environments in which they live. Families exist within societies and provide their children with opportunities for healthy nutrition and being active based on the environments in which they live and the resources available to them. Spheres of influence include the child and their family and their community (including ECEC settings, public spaces and infrastructure, public transport), and societal and political influences (industry, agriculture, media, transport and planning, healthcare, and social norms) (13). To prevent “fade-out effects” such as seen in the EPOCH trials it is prudent to align early childhood obesity prevention interventions with broad environmental actions to prevent obesity (14). That is, to consider broad social/whole of population strategies along with specific interventions for families during the first 2,000 days. However, a recent study found that policies for this life stage tend to focus on support programs for parents (usually mothers) and more recently the ECEC sector (7).

Where we live matters. A 2017 Australian health analysis found people in the lowest two quintiles by socioeconomic status have significantly increased risk of poor health outcomes (15). The proportion of the population in the two lowest socioeconomic status quintiles differ across jurisdictions—while 4.2% of the population in the ACT are in the lowest two quintiles, for Tasmania it is 63.3% (16). There are key contextual differences between Australian jurisdictions, including population size and density, budgets, and degree of rurality. Australia's urban populations (just over 70%) experience determinants of health very differently to the almost 30% of the population in non-metropolitan areas (including rural, regional and remote) (17). See Supplementary File 1.1 for a summary of key demographic differences between the jurisdictions.

Given the complexity of childhood obesity prevention, it is important to examine “where we are” and “why we are.” Frameworks for obesity prevention consider these spheres of influence noted above and identify points where governments can influence and possibly prevent it. Systems approaches and sense-making frameworks (18, 19) seek to identify key areas where public policy can influence both lifestyle behaviors and the wider determinants of health. Systems thinking places a “high value on understanding context and looking for connections between the parts, actors and processes of the system” (20) and aligns strongly with ecological models which consider the social determinants of health (21). A recent study of 18 Australian policy-makers found a trend toward the uptake of systems thinking in developing “new prevention narratives,” although a minority were unclear of its utility and methods (21), suggesting emerging opportunities for collaborative partnerships.

We have previously undertaken a comparison of national policies for the early prevention of obesity in childhood for Australia compared to five similar countries (7). The present study had two aims. The first aim was to provide a snapshot of policies for the early prevention of obesity in childhood, across the public health spectrum, at the state and territory level in Australia. The second aim of this study was to explore the perspectives of senior state and territory health department officials about their experiences and the local context of developing and implementing policy options for childhood obesity prevention. To our knowledge, this is the first publication of cross-sectoral policy mapping for obesity prevention in the early years among Australia's jurisdictions.

## Methods

### Study Design

We conducted a qualitative study of early childhood obesity prevention policy (including prevention programs and initiatives) among Australian states and territories using (1) policy mapping and (2) semi-structured interviews with senior health officials who have responsibility for obesity policy. The purpose of the policy mapping was to provide context and evidence of government policy in addition to the subjective responses of the participant interviews.

### Policy Mapping and Analysis

#### Tool Development

A policy mapping tool was adapted to the Australian state and territory context from the *WHO Ending Childhood Obesity Report* with additional supportive literature (Supplementary File 1.2), to develop guiding questions to prompt policy searches. This report provides an action plan to “translate evidence into practice” emphasizing the importance of regulation (22). This adaption included public health approaches to obesity across the social model of health (23) and built upon an earlier Australian policy mapping analysis (7) that identified state and territory governments) policy responsibilities. The broad policy areas were governance, health supportive environments, ECEC settings, and health services aimed at the first 2,000 days. The policy mapping provided a snapshot of key policy examples for early childhood obesity prevention across Australian jurisdictions.

#### Mapping

We identified relevant government agencies in each state and territory, developed a search strategy, and extracted data. The policy search was an iterative process undertaken by EE commencing 1 October 2018 (prior to interviews in late 2018) with follow-up after interviews prior to mapping being finalized on 30 June 2019. The online search used key words, from the guiding questions of the policy mapping tool, in embedded search engines in identified agency websites. These searches were augmented by the advanced search tool function in the Google search engine [described in a previous study (7)]. To minimize bias the incognito function was used, the researcher browser history, cache, and cookies were cleared, and regional settings were used to localize results to Australia.

#### Analysis

Data were extracted by EE (and reviewed by CR) using a policy content analysis approach (24) and included policy name and a description of how policies were being used to achieve the elements of the guiding principles, or ways that they could potentially be leveraged to do so, and an overall rating was given. The ratings are described in Table 1.

**Table 1:** Ratings and descriptions for the policy mapping analysis.

**Result**		Description^*^
	Policy (or initiative) in place	There is a policy or initiative in place that aligns to the guiding question. This does not mean the policy has been implemented or evaluated for effectiveness.
	Policy Infrastructure	Moderate alignment to existing policies or frameworks. There are many elements in place but to extend or develop a policy in this space requires input from key stakeholders to develop or adapt to local context.
	Policy Scaffolding	Low alignment to existing policies or frameworks, however, there is some potential (in a single or multiple policy settings) for development of a policy or program in this area.
	Policy Void	No policies were found at the time of mapping, or an absence of alignment. In some instances, policies were not contextually relevant or possible for that jurisdiction (in which case it is noted in Supplementary File 2).

* *Mapped policies were publicly available online. It is likely that some policies are in existence but were not found in the desktop review nor provided by jurisdictional informants at the time of interview*.

### Interviews With Senior Health Officials

A semi-structured interview tool was developed based on systems approaches to obesity prevention and adapted to each jurisdiction (see Supplementary File 1.3). Ethics approval for this project was granted by the University of Sydney Human Research Ethics Committee (Project 2017/507).

Purposive snowball sampling was used to identify potential informants through the professional networks of the authors and their colleagues. Senior officials with current active responsibility for obesity prevention in each jurisdiction's health department—a handful in each jurisdiction—were eligible for participation (inclusion/exclusion criteria). Prospective participants were invited to participate via email. Three attempts were made to reach identified informants before attempts were made to contact another informant. In three instances, the person invited referred our invitation to a colleague within the same branch, who then accepted. In total, nine informants were recruited (of 12 invited) from Australian state and territory health departments, one from each jurisdiction except the Northern Territory, which had two informants. All participants contributed to the development and/or implementation of obesity prevention policy and programs. Interviews with state and territory informants were conducted between November and December 2018. First order coding after each interview was performed to ensure saturation point had been achieved while ensuring one interview per jurisdiction as a minimum for equal representation. Interviews averaged 63 min (range: 42–95 min).

All interviews were conducted via telephone, recorded and transcribed verbatim. Interviews were coded using thematic analysis in NVivo 10 software. All data coding and extraction were undertaken by EE. CR and LMW cross-referenced a sample of interviews to ensure a consistent coding frame. Thematic analysis (25, 26) is a tool or a method to identify, analyse, and interpret meaning—“themes”—from qualitative data. The themes provided structure to report on research findings separate to or with the use of theoretical frameworks.

## Results

The results of this study are presented in four sections. The first section summarized the policy mapping and describes a key finding of the policy mapping and interviews with senior health officials—that Australia has two distinct local health promotion models. The second and third sections describe the approaches taken to collaborate across government agencies and health supportive environments. The final section identifies key political drivers and policy levers for obesity prevention.

### Policy Mapping and Local Health Promotion Models

References to policy mapping are indicated by their Guiding Question (GQ) area or specific identifier, e.g., (GQ area A) or (GQ A.1.1) throughout the results. The policy mapping found that childhood obesity was identified as a problem in most jurisdictions (GQ A.1.1). The key life stages of pregnancy and/or early childhood (or as the first 2,000 days) were less well-defined in key strategic documents (GQ A.1.2). Having an overarching policy framework or strategy to address obesity/childhood obesity (GQ A.3) did not guarantee action or implementation plans in the areas of health supportive environments, ECEC settings, or health settings. Instead, the language used to describe the causes of obesity and to identify policy action areas were a better indication of policy infrastructure available across these areas. For the most part the initiatives that flowed out from the key strategic frameworks in Areas B–D were focused on increasing skills and knowledge at the family level, whereas the language to describe the structural causes of obesity in the context of policy options was vague, e.g., “partnerships to improve environments.” Where clear language was used to identify specific areas (e.g., food advertising) as contributing to obesity in key policy documents, specific policies to address the social determinants of health and health supportive environments were more likely. Less than half of jurisdictions had statewide funded programs to support food and physical activity environments and curriculum in ECEC services (GQ area C.1). While antenatal care and child health services/universal checks were present in all jurisdictions (GQs D.1.1, D.2.1), sub-elements within these areas were less prevalent. Additionally, programs aimed at obesity prevention across the first 2,000 days were limited (GQ D2.2.2). The limitations of these areas followed workforce capacity considerations such as training and resources (GQ area D.3). Policy mapping results were tabulated and ranked, as shown in Table 2. A one page summary and the full policy mapping results, augmented with quotes from participants, can be found in Supplementary File 2.

**Table 2:** Policy mapping tabulated results, by Health Promotion Model type.

**Area**	**Guiding questions**	**Local Government**				**Local health network**			
		**ACT**	**SA**	**Vic**	**WA**	**NSW**	**NT**	**Qld**	**Tas**
**A. Governance and leadership**									
A.1 Leadership	A.1.1 Has childhood obesity prevention been identified as a priority by leadership?								
	A.1.2 Is there an overarching policy framework, or a series of key policies or action plans to guide initiatives for the early prevention of obesity in childhood?								
	A.1.3 Does public health legislation include prevention/health and wellbeing?								
	A.1.4 Are their statutory grant-giving bodies with a remit to fund prevention-related community projects?								
A.2 Partnerships	A.2.1 Are partnerships across government noted in “key policy” identified above?								
	A.2.2 Are there formal mechanisms for collaborative exchange across sectors?								
A.3 Equity	A.3.1 Do the key policies identified outline the structural causes of obesity?								
	A.3.1a Do recommendations for action address these structural causes?								
	A.3.2 Are target populations, with higher risk of developing obesity, identified for additional support?								
**B. Environments in which we live**									
B.1 Health supportive environments	B.1.1 Do planning policies orientate built environments toward principles of active living?								
	B.1.2 Are there investments for public infrastructure (e.g., footpaths or bikeways) to encourage being active?								
	B.1.3 Are there food/nutrition policies aimed at ensuring a nutritious, affordable, accessible food system?								
	B.1.4 Are there programs to support vendors to improve food offerings in food outlets (restaurants, cafes, take-away, vending machines)?								
	B.1.5 Is nutrition information at food outlets (menu board labeling) required by legislation?								
	B.1.6 Is there engagement with food retail (supermarkets, grocers, corner stores, etc.) to reduce the availability and promotion of discretionary choices in-store?								
	B.1.7 Are local governments empowered to encourage health-supportive environments?								
	B.1.8 Are there any initiatives to reduce exposure to the marketing/promotion of discretionary choices in:								
	B.1.8a out-of-home advertising within government control?								
	B.1.8b healthcare settings?								
	B.1.8c other government-controlled buildings/parks?								
	B.1.9 Are there policies limiting the availability/provision of discretionary choices in:								
	B.1.9a healthcare settings (for visitors and staff)?								
	B.1.9b buildings, community centers, and parks under government control?								
B.2 Health promotion campaigns	B.2.1 Are there health promotion campaigns aimed at encouraging healthy lifestyle behaviors?								
	B.2.2 Are there health promotion campaigns aimed at developing/supporting healthy food systems and built environments (incl. community-capacity building)?								
**C. ECEC settings**									
C.1 ECEC settings	C.1.1 Are there support programs for center-based care settings to encourage healthy food provision?								
	C.1.2 Are there programs to support provision of food and physical activity experiences as part of the curriculum?								
**D. Health**									
D.1 Antenatal and birth services	D.1.1 Does antenatal care screen and manage hypertension, hyperglycemia, appropriate gestational weight gain?								
	D.1.2 Antenatal care within public health services:								
	D.1.2a Do they include nutrition counseling for healthy pregnancy or are there other healthy lifestyle support programs available during pregnancy?								
	D.1.2b Is breastfeeding education free (standalone or embedded into services)?								
	D.1.3 Do maternity facilities fully adhere to the Baby Friendly Health Initiative (based on 10 *Steps to Successful Breastfeeding*)?								
D.2 Early childhood health services	D.2.1 Are there free health/parenting services to support early childhood growth/nutrition (e.g., breastfeeding, complementary feeding, transition to family foods)?								
	D.2.1a Is information to support parents readily available (e.g., phonelines, websites)?								
	D.2.1b Do these include breastfeeding support?								
	D.2.2 Are there healthy lifestyle (education) programs to support families during early childhood?								
	D.2.2a Are target populations identified and actively recruited for programs?								
	D.2.3 Are Supported Playgroups offered for families that need additional support and do they include healthy lifestyle skills?								
D.3 Workforce	D.3.1 Are there training and resources available for health care professionals to support families?								
	D.3.1a Is preconception advice for nutrition and being active provided to prospective parents?								
	D.3.2 Is there a state/territory health promotion…								
	D.3.2a …agency (independent or adjunct to health department)?								
	D.3.2b …workforce (to implement initiatives locally)?								

*ACT, Australian Capital Territory; SA, South Australia; Vic, Victoria; WA, Western Australia; NSW, New South Wales; NT, Northern Territory; Qld, Queensland; Tas, Tasmania*.

*Legend 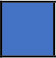 Policy in place 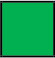 Policy infrastructure 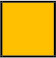 Policy scaffolding 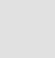 Policy void*.

Policy mapping indicated that no two jurisdictions were the same in their approach to obesity prevention. Policies were eclectic and sporadic, rather than coordinated or long-term. These heterogenous results emerged out of the different health promotion contexts which have developed across Australia. We found that Australian state and territory governments have developed unique authorizing environments for obesity prevention. Across Australia's six states and two territories there were broadly two local Health Promotion Model (HPM) types. These HPMs relate to the eclectic practices of local program delivery, the presence and structure of Local Hospital Networks (LHN) (see Supplementary File 1.1), and the extent of involvement from Local Governments (LG).

In the first local HPM, obesity prevention activity is primarily delivered through local hospital or health networks, which link hospitals and population health services across a geographic area (LHN HPM). In the second, health promotion activity is primarily driven through local government (LG HPM). Table 2 is organized by these two HPM types.

The LHN HPM was typified by NSW and included the NT, Queensland, and Tasmania. In NSW the authorizing environment sat under the Premier's Priority to reduce childhood obesity by 5%. The health department developed a statewide prevention strategy (*Healthy Eating and Active Living*), and the Office of Preventive Health delivered the *Healthy Children Initiative* with settings-based approach (Supplementary File 2.2). *Munch & Move* is a program to improve ECEC settings delivered by a dedicated health promotion workforce embedded in health promotion units of the 15 Local Health Districts in NSW. It is the main state-wide early years initiative in NSW. Another program funded by the Office of Preventive Health is the *Get Healthy in Pregnancy* program (a coaching services delivered via telephone and managed by a third-party provider). Under this model, the Office of Preventive Health provides centralized support and strategic direction to Local Health Districts for specific settings and does not offer centralized support for healthy food and built environments.

In 2013 Queensland lost its dedicated health promotion workforce embedded in its Hospital and Health Services (see Supplementary File 2.4, GQ D.3.2). It does have Children's Health Queensland, a state-wide Hospital and Health Service aimed at health service delivery for children. However, at the time of mapping and interviews Children's Health Queensland lacked state-wide early childhood programs for obesity prevention. Recent election commitments were made to rebuild the health promotion capacity and a new authorizing environment for prevention was established, including the *Health and Wellbeing Strategic Framework* (27), and Health & Wellbeing Queensland as an independent health promotion agency. Queensland led intergovernmental work for childhood obesity prevention and the development of the national obesity strategy.

The *Healthy Tasmania Five Year Strategic Plan* (28) is guided by the Premier's Health and Wellbeing Council and identifies the early years as a key life stage for health (Supplementary File 2.6). Tasmanian approaches to preventive health implementation drew from previous experiences of success at a community level. The Tasmanian participant noted that several initiatives/programs have been running for more than 10 years, including *Neighborhood Houses, Eat Well Tasmania*, and *Family Food Patch*.

While historically child health has been more focused on remote communities in the NT, there has been a shift toward whole-of-NT child health services which includes emerging attention toward obesity prevention (Supplementary File 2.3). This is supported by the 10 year *Starting Early for a Better Future* (29) strategy. The *NT Nutrition and Physical Activity Strategy* (30) has five objectives including remote food security, healthy gestational weight and an early years focus. NT has the highest proportion of people living in remote areas, as well as the highest proportion of First Nations people. NT Health is working with the Aboriginal Health Forum to develop standardized reporting against national BMI key performance indicators across NT. The Northern Territory and Tasmania have very small local governments, with very limited capacity in some instances to participate in health promotion activity:

“*There is huge disparity between those local government councils that have got big capacity, versus those that have got little capacity… we've got 29 local government councils for half a million people” (Tasmania)*

The second type, LG HPM, was typified by Victoria, but also included SA, WA, and the ACT. Victoria, SA, and WA have pivoted toward LG responsibility for health promotion, each updating their Public Health Acts, while the ACT acts as both territory and LG. A state public health plan is the primary central mechanism for health promotion work, and LGs are required to develop local plans in response.

Victoria has had a more fragmented public health administrative structure, historically, local government has played a much greater role than in other states (Supplementary File 2.7). In addition to the health promotion goals of the *Victorian Public Health and Wellbeing Plan* (31), Victoria's *Early Years Compact* (32) is an agreement between health, education, and LGs to ensure continuity of services across the early years. Local councils are also supported through nine Regional Assemblies by regional arms of state departments. VicHealth is a statutory health promotion foundation that provides structural support to LGs and community health promotion projects. As VicHealth is independent it has the autonomy to be able to take a longer-term view to health promotion, advocate for broad public health actions at the state and territory level, and challenge industries that are harmful to health. VicHealth also runs health promotion campaigns (GQ B.2) aimed not only at personal/family behaviors, but also aimed at changing cultural norms.

Western Australia's *Sustainable Health Review* (33) has underpinned a significant amount of recent and ongoing health system change. The WA *Public Health Act 2016* (34) requires a state-wide State Public Health Plan (Chief Health Officer) and Local Public Health plans (from each local district). The first objective of the *State Public Health Plan* (35) is to enable healthy living (including healthy eating, being active, reducing sedentary time) and is supported by the *WA Health Promotion Strategic Framework* (36). At the time of mapping, LGs were not yet required to develop their local public health plans, although some had already commenced these activities (see Supplementary File 2.8, GQ A.1.3). The *Western Australian Health Promotion Foundation Act 2016* (37) merged two grant-giving bodies, Healthway (~$20 million spend) and Lotterywest (~$260 million community grant spend):

“*The two organizations merging together provided some efficiency in terms of a shared corporate governance system, but also had potential to expand the reach and influence of Healthway and its messages” (Western Australia)*.

The *SA Public Health Act 2011* (38) established the new health promotion model in SA, requiring LGs to respond to state public health plans. The Act enables partnerships at the local level to connect LG and Public Health Partner Authorities with state entities, e.g., in planning, transport, and environment. Although SA has established strong cross-government mechanisms, the SA participant noted that the most recent state health plan could have gone further to promote a whole of government approach to prevention:

“*It missed the mark to address the whole government agenda, it's a bit of a gap at the moment” (South Australia)*

SA also recently updated their planning laws, which centralized authority on local planning decisions to state authority. At the time of mapping, there appeared to be little additional structural support for LGs. Like Queensland, SA lost their health promotion workforce in 2013 under a review of health system services (Supplementary File 2.5, GQ area D.3). *Wellbeing SA* was being established at the time of mapping, designated to be a health promotion agency within the health department, although without the independence or funding capacity of other similar organizations (e.g., VicHealth, Lotterywest, Health & Wellbeing Queensland).

For both SA and WA their LHNs provide additional health promotion support to LGs. Like Victoria, the ACT has no LHNs (Supplementary File 1.1). While the ACT Public Health Act does not specify principles of prevention and well-being, a core role of the Chief Health Officer is prevention:

“*The Public Health Act tends to be… a bit old school public health… There is still a focus though on protecting and promoting the health of the population as a key role and a statutory function of the Chief Health Officer” (Australian Capital Territory)*

The uniqueness of the ACT as both territory and LG and the absence of LHNs, aligns the ACT more with the LG HPM (Supplementary File 2.1). Its *Healthy Weight Initiative* leveraged this capacity to deliver on projects that normally require at least two levels of government (more on this in the next section).

While jurisdictions were similar in the leadership areas under both models (GQ A.1), the LG HPM jurisdictions have more policies in all areas, most notably in the health supportive environments areas (see Table 2). Policies and policy infrastructure were more likely under the LG HPM for health promotion workforce (GQ area D.3), universal child health checks and parent support lines/online information (GQ D.2.1), health supportive environments (GQ B.1), and a stronger emphasis on equity and partnerships (GQ areas A.2–A.3). Policies and policy infrastructure were more likely under the LHN HPM for statewide universal healthy lifestyle programs and targeted programs for families needing additional support (GQs D.2.2–D.2.3), antenatal care (GQ area D.1), and slightly more likely for health promotion campaigns (GQ area B.2). However, these associations are not necessarily causally related.

Health departments in most jurisdictions engage with the strong community not-for-profit sector who have positive community standing to deliver programs or fill service gaps. For example, Nutrition Australia is a not-for-profit health promotion organization with branches in most jurisdictions. In their ACT and Queensland branches, they offer pay-for-service programs into the ECEC sector where neither funding nor health promotion capacity is forthcoming from government. In contrast, the Victorian branch of Nutrition Australia is funded directly by the health department to deliver the *Healthy Eating Advisory Service* (alongside the Cancer Council who deliver the *Achievement Program*) into ECEC settings (Supplementary File 2.7, GQ C.1). In Victoria's service model delivery, the health department does not deliver services but funds a mixture of government and non-government agencies to provide services. These “frontline” organizations provide feedback on community needs and contribute to the evidence base used to develop policy in Victoria. Health departments use a range of different ways to report on the progress of policy implementation, presenting an opportunity to contribute to building evidence and justify ongoing investment into initiatives, e.g., Public Health Information Management System in NSW (Supplementary File 2.1, area D.3).

Participants noted the use of a range of structural elements to keep health promotion and prevention on the agenda. These included updated public health (Victoria, SA, WA) and planning acts (Queensland, Tasmania, SA) and legislation to include/consider the prevention side of public health and well-being, and the establishment of statutory agencies (Victoria, NSW, Queensland) with a health promotion and prevention remit. Several jurisdictions noted the waxing and waning of mandates to progress prevention policy is influenced by political ideology.

Partnerships that developed through these HPMs were at the local health network or council level and may include local implementation arms of state/territory departments such as transport or planning. This is different to the centralized health promotion work managed by health departments in conjunction with other state/territory government agencies in a more top-down approach. That work tends to focus on strategic partnerships and initiatives at the state/territory or regional level, which is the context for the next sections.

### Approaches to Collaboration Across Government Agencies

This study identified many themes and subthemes from the interviews and policy mapping. Table 3 provides a summary of these themes sorted into enablers and barriers to policy development and implementation. References to the appropriate policy mapping area are indicated throughout the text.

**Table 3:** Summary of themes—barriers and enablers.

**Enablers**	**Barriers**
Leadership enablers •Provide mandate •Facilitate structural support incl. legislation, strategic oversight, funding •Proactive investment •Ministerial support beyond health	Limitations of leadership •Perception of health “imperialism” •Perception that health is already well-funded •Funding and workforce capacity •No enduring structural support for prevention •Personal beliefs of ministers •Preference for immediacy, visibility
Governance •Flexibility in policy design •Flexible funding considerations •Clearly defined scope •Multi-strategy approaches •Long-term strategies and investment •Creative approaches to policy e.g., use of legal infrastructure (not regulatory)	Governance •Focus on health services/family-directed •Departmental restructuring, workforce implications •Nature of short policy cycles, conflict with long term investment for prevention •Limitations on policy experimentation
Collaboration enablers •Partnership approach, minimize competition •Policy co-production •Commitment to participate (by all parties) and maintaining relationships •Supporting submissions/business cases •Ongoing mechanisms for collaboration to build off existing successes •National funding supports longer-term investment, policy experimentation and sharing	Barriers to Collaboration •*Ad hoc* partnerships •No or limited understanding of other agencies priorities
Discourse •Physical activity initiatives have more positive narratives, e.g., social connectivity, seen as giving people more	Discourse •Nutrition initiatives have negative narratives, e.g., “nanny state” or taking something away from people •Economic rationalism •“Personal responsibility” •Resistance to regulatory pathways
Evidence •Evidence of policy efficacy and impacts across sectors •Policy experimentation •Re-framing narrative to “evidence-informed” rather than “evidenced-based”	Evidence •Attribution tricky •Trend toward single initiatives focus on the individual/families
Economic •A place for well-being •Liveability, population growth •Food tourism	Economic •Industry influence
Other •Public acceptability	Other •Poor communication with the public •Hyper-focus on obesity narrative

This section explores approaches to collaboration across government agencies, commencing with two brief case studies (ACT, SA) before exploring broader experiences across all states and territories. Consistent enablers were ongoing support from leadership, clearly defined scope, flexibility, shared outcomes and successes, and incremental change. Barriers to collaboration across sectors were perceptions of health “imperialism,” limits on leadership, departmental restructuring, and funding or workforce capacity.

The ACT has a well-developed approach to cross-government prevention (39). From 2011 the health department had a mandate from their Chief Minister, who was also the Minister for Health, to undertake several years of collaborative work across sectors, including commissioning research and partnering with academics and non-government health promotion organizations to provide an evidence base for population approaches to obesity prevention. In developing their *Healthy Weight Action Plan* (40) the ACT had multiple discussions across government and invited experts:

“*…into the room with us. And we talked through [the evidence and]…what people have suggested in terms of their contributions from a Directorate [government agency] perspective and what was achievable and what wasn't” (Australian Capital Territory)*

The key factors identified as helping in this policy development were a clearly defined scope, inviting perspectives from all relevant agencies about what might be feasible or not, and having agencies take ownership of specific policy areas. At the core of implementation was a clear mechanism for collaborative work across agencies, that brought:

“*…the whole of government together and having really good mechanisms of working across government to achieve big policy outcomes” (Australian Capital Territory)*

Similarly, Health in All Policies (HiAP) in SA adopted a broader whole-of-government approach (41–44). This benefited from significant support from state leadership over an extended period. Starting in 2007, with an opportunity to experiment with policy innovation, was followed by an ongoing mandate to build on their community of practice for over 12 years. The *SA Public Health Act 2011* established partnerships across government (vertical and horizontal) and principles such as the equity principle required by the Act, which also enables the *State Public Health Plan*, create a long-term enabling environment beyond single policy cycles (Supplementary File 2.5, area A). This in turn supported developing relationships in an ongoing manner, reflecting:

“*…an evolutionary change in the South Australian public sector policy group where we've all been learning from each other and incrementally, hopefully, getting better at doing it” (South Australia)*

This authorizing environment, over time, changed public sector culture toward collaboration. If the policy workforce tends to stay in the public sector, even as they move across agencies, it enriches these networks over time—although this may be more possible in SA with a smaller bureaucracy (like the ACT) than other jurisdictions:

“*Most policy people… they might move around [the public sector] …but they don't leave it” (South Australia)*

With a mandate, SA were able to develop their own HiAP methods appropriate to an Australian context over time, such as Health Lens Analysis and 90-day Projects (Supplementary File 2.5, area A). They took the view that starting with a determinants approach opened up the dialogue with other government departments by making opportunities for alignment more explicit and:

“*… working with them in ways that respects their understanding and their ways of knowing and their evidence approaches… The co-design methodology, the shared agenda, the shared responsibility, the finding of common solutions acceptable to all, is the cornerstone of our approach” (South Australia)*

While many jurisdictions supported the principles of HiAP several noted barriers to using the approach, such as its time-consuming methods. They also noted the language appears to preference health which may put-off other agencies they are trying to engage, two jurisdictions referring to their own processes of collaboration as HiAP “by stealth” (WA, ACT). SA recognized that HiAP has a different meaning in other jurisdictions:

“*…for South Australia people know what that means in the policy world. So the understanding of what it is and how we work precedes the name” (South Australia)*

Elements identified for successful engagement across government include having a mandate from leadership and the provision of concurrent structural opportunities to undertake the work. These include long-term strategic policies (Queensland, WA, ACT), Public Health Acts which ensure the long-term maintenance of a health and well-being mandate (Victoria, SA, WA), and a dedicated health promotion workforce (NSW). No jurisdiction had all of these elements at the time of mapping.

Participants noted barriers to engagement with other sectors included the perception that health already has a lot of funding to deliver on their core business and health's “imperialist” reputation (based on historical context). These barriers can be overcome by taking steps to understand the priorities of those agencies:

“*we take that whole government lens, without being health imperialistic… actually asking people, ‘What do you guys do?' And then assessing where there are elements of co-production that they may not have been aware of…” (Australian Capital Territory)*

Informants from all jurisdictions felt that it was the role of health departments to make connections with external partners to prevent obesity. However, as their capacity to do so can be limited the default becomes *ad hoc* relationships or negotiating to the point where strategies are developed but initiatives not implemented (and an expectation that other agencies will take the lead). Participants noted that health departments were clear on the actions needed, and which departments were responsible, but have neither the authority nor the capacity to lead other agencies. When health departments do have capacity to seek policy alignment with other sectors, participants noted elements that supported success. These included minimizing competition between agencies and taking pro-active investment for capacity building. Partnerships developed out from offering support, identifying common ground, working on small projects, developing good will, maintaining relationships, and co-defining problems and solutions. This process of alignment supported the sustainability of cross-government relationships by finding solutions that both appease “the hierarchy” and focused on shared outcomes:

“*So that means that the agencies that we partner with have to be prepared to put people around the table in a consistent way and we have to listen to them… it doesn't mean there's not tension, but it's generally characterized with positive outcomes” (South Australia)*

It also means being creative in filling some of the capacity gaps in other sectors, for example funding positions in other agencies to ensure a health lens is included in policy formation and implementation. Study participants highlighted some examples such as Victoria funding health promotion positions in LGs during the *Healthy Together Victoria* initiative. The ACT funds an official in education to act as a health-education nexus, and Tasmania has a HiAP-trained health official seconded to contribute to liveability projects with the Department of Premier and Cabinet. Other examples were given of health providing capacity support for funding:

“*You need to apply quite a lot of ingenuity to get things done… We will sometimes partner or provide letters of support, to other agencies (government, NGOs, research groups), when it comes to funding submissions…” (Western Australia)*

“*So how can we support each other even in business cases and submissions to government and things like that, to do things with mutual benefits” (Australian Capital Territory)*

Some participants also talked about utilizing different parts of the health department to engage in different activities in order to maintain relationships, by:

“*…seeking mutual gains – the ‘carrot' approach… [however], where a mutual gain outcome is not possible… [it] is not our role [to push for an outcome] as this work would compromise our positive relationship. So, we need other players in the health department to play the ‘stick' role” (South Australia)*

Some jurisdictions (ACT, SA, WA) noted other sectors initiating engagement with health when reviewing or updating their own high level policy frameworks in recent years (GQ A.2.2). This indicates that cross-government work is being considered and there is a growing willingness to harmonize strategies. Examples include harmonizing active travel policies with emissions reduction targets for climate change (ACT) and reducing traffic congestion (ACT, NSW, WA, Victoria), and planning legislation updates (Queensland, SA, ACT) were developed in partnership with health. While collaboration on prevention is desirable it is not core business for most agencies so when funding contracts, whole of government work is unlikely to continue in the absence of structural support such as the methodologies undertaken in SA and ACT (GQs A.1.2, A.2.2), or public health acts that embody partnership principles (SA, Victoria—Supplementary Files 2.5, 2.7, GQ A.1.3).

Childhood obesity is widely recognized as a public health problem, requiring collaboration across sectors to implement “multiple strategic approaches” (Tasmania). However, the specific mix of interventions and investments needed to address it are not yet known:

“*Determining the dose, scale, volume and mix of a variety of types of interventions… remains a challenge” (Tasmania)*

While the role of environments in obesity prevention are becoming more accepted, where and how to act is less well-understood. Participants noted the challenges in pursuing environment policy where the evidence was less clear about how to translate or scale up in different jurisdictional contexts or making the business case for economic investment:

“*It's also quite difficult to make some of the economic arguments around it because attribution is so challenging” (Australian Capital Territory)*

“*There might be some windows coming up soon, but we probably need a little bit more evidence from where other places have tried to do this sort of work” (Tasmania)*

The next section explores the key components of health supportive environments through the lenses of physical activity and food/nutrition.

### Health Supportive Environments

The themes that emerged about policy development and implementation for health supportive environments were the importance of leadership beyond health, the interplay of positive and negative discourses about physical activity and nutrition, and the influence of industry. Successful strategies took a long-term multi-strategy approach, building upon successive policies and looked beyond single strategies aimed solely at parents.

By promoting the mental health benefits and social and community connectedness, and its impacts on learning, rather than a focus on an energy balance or physical fitness alone, physical activity policies have gained more traction with departments beyond health, notably education, planning and transport (active living/transport features in most jurisdictions).

The promotion of the social benefits of an active population and environmental considerations (such as creating and protecting green space), influenced Queensland, Tasmania, ACT, SA, and WA to add broader principles of health and well-being and “liveability” to their planning laws. For planning:

“*…terms like liveability and wellbeing are big important issues there. People would rarely think about obesity though, outside of Health” (Queensland)*

“*In the Act… developers, for example, have to address the active living principles in their application… So yeah, walkability and liveability are key considerations” (Australian Capital Territory)*

In SA, the impact of recent changes to the Planning Act on health will depend on new compliance rules under development at the time of this study. SA health and environment departments were partnering to support the planning department in developing compliance rules to support healthy built environments, in turn supported by Cabinet. However, this process came with resistance from other players in the built environment:

“*Well, industry is lobbying, of course, the government. The Department of Planning is drafting the guidelines, so we are consulting with them. We're trying to help shape and inform the way they do it, but they've got lots of needs to balance… And we're working really hard (and to some small success) to increase the focus on ‘healthy liveable neighborhoods'… The work we do with the Environment Department is… really about increasing the community's re-connection with nature and open green space… So there's tension and that tension is being played out here, but the Environment Department and the Health Department are working together to present a united voice to the Planning Department” (South Australia)*

A study was undertaken in the ACT that demonstrated the connection between physical activity and academic outcomes in school (GQ B.1.1). This supported engagement with education about physical activity and alignment with the national curriculum. However, this alignment has not driven similar approaches to ECEC settings (GQ area C.1). The ACT also commissioned research to look at both physical activity and food environments, they:

“*…saw some evidence of effects, particularly in the physical activity space, but in terms of nutrition and… the food environment, there was really nothing, the best we can hope for there was sort of ‘promising' things as evaluated by them” (Australian Capital Territory)*

The physical activity evidence was another lever that made engaging with other agencies more straightforward. The limitations of evidence for food environments were an extension of the limitations in government monitoring of food environments. The issues raised with the availability and use of evidence was a recurring theme, explored further in the next section.

Some study participants noted that it was politically easier to promote physical activity because of positive messaging attributes—being active “gives” you more—whereas a lot of the messaging about nutrition comes across as restricting people. The high attribution to personal responsibility and a concurrent concern about being perceived as infringing on personal choice (i.e., “nanny-state” approaches) can result in policy choices regressing toward personal/family skills and knowledge unless efforts are made to gain public interest:

“*A lot of those things by default can come back to people, knowledge and skills” (Queensland)*

At the same time, advocates for the food and advertising industries can influence politicians across multiple sectors, and interrupt efforts to act in the food environment, especially when less “scientific” evidence is available. Food manufacturing and the head offices for food retail are limited to two or three jurisdictions, influencing how policy makers act:

“*I think for the NT because we're a small jurisdiction and we don't have big manufacturers, we're not bombarded as much, as such. So we don't have that pressure they have in other jurisdictions” (Northern Territory 2)*

“*We probably don't have the same issues that Victoria and New South Wales have, in that we haven't got a big commercial manufacturing sector, that's constantly lobbying our government” (Tasmania)*

Despite these barriers, there are examples of leadership in food environment policy. The ACT and Victoria have policies to remove all promotion of discretionary foods and drinks from government-controlled settings (GQs B.1.8b-c, B.1.9). The ACT has enacted policy that prohibits discretionary foods and drinks from their out-of-home advertising assets on their bus network, and at the time of mapping Queensland had announced a policy to prohibit discretionary foods and drinks from all government-owned assets (GQ B.1.8a). For the ACT, the transport minister announced the decision to remove discretionary foods and beverages food from public buses, a policy which was supported by Health to implement. It was:

“…*relatively out of the blue … And obviously we've had good outcomes in that people [department revenue or advertising companies] haven't lost money so the world didn't explode because we don't advertise [fast food]… And I think we should look to extend it, frankly, to other modes of travel” (Australian Capital Territory)*

Key to the policy success (and permanence), was monitoring the potential fiscal outcomes and generating evidence that the policy did not cause a net loss to the transport department. Other jurisdictions note hesitancy and taking a slow approach in the out-of-home advertising policy space. Participants in different jurisdictions noted that barriers to this policy lever include the perception that a non-health agency may lose revenue from advertising on their assets (usually transport), seemingly unaware of the evidence available from the ACT, and hesitancy to implement a policy that might have negative public blow back:

“*[The concern is] …the transport department may temporarily lose funds if they do a lot of advertising of unhealthy food and drink on public transport vehicles and bus stops… I think it's early days in this space” (South Australia)*

“*…the government probably wants to see how [the introduction of a ban of alcohol advertising on public transport infrastructure] plays out before it looks to expanding that to junk food for instance” (Western Australia)*

In the ACT, it was the cross-government mechanisms and supportive policy environment that allowed the expansion of healthy food availability and promotion policies from health and school settings to all government buildings and assets across the ACT (GQs B.1.8–B.1.9). By starting with health and education sectors, using consistent criteria, and offering support through the ACT Nutrition Support Service (developed and delivered by Nutrition Australia ACT, a not-for-profit), it gave suppliers and vendors an opportunity to grow to meet a new food supply demand, and then expand into other government settings. It also provided opportunities for businesses to expand their offerings more widely in the community, and for health to establish partnerships with business representatives and to co-create evidence of economic viability. At the same time the partnership with Nutrition Australia ACT established an ongoing workforce who specialize in partnering with businesses to improve their food offerings, which carried over to the *Healthier Choices Canberra* (GQs B.1.4, B.1.6) program:

“*[It] has unbelievably popular with businesses… We have relationships with the Canberra Business Chamber through the… program and that has been amazingly useful and beneficial in terms of being able to bring businesses along and really getting them to see themselves as a partner in establishing that there is in fact a market” (Australian Capital Territory)*

Some programs exist to support better stocking practices and promotion signaling in local food retail (NT, ACT), or to support the sport and recreation sector to establish appropriate sponsorship (i.e., not from fast food) while maintaining their capacity to attract funding (ACT). Support for local stocking practices in food retail can be very different for urban vs. remote communities. While in the ACT this included using information tags on products to promote comparable healthier options in-store, in remote NT communities it can be around the cost of healthy food and making sure appropriate infant foods are available at all (Supplementary Files 2.1, 2.3, GQ B.1.6).

Support for foodservice outlets (GQ B.1.4) included engaging with training institutes to build capacity among the hospitality workforce (ACT, Tas) and engaging with businesses to develop healthy food options on menus (ACT) or children's menus (SA). Take away food outlets in remote areas have been flagged as a potential element to increasing rates of obesity and chronic disease in some remote communities in the NT:

“*Take-away stores are becoming more prevalent and affecting the local food environments. With longer opening hours than remote stores, some concerns have been raised about the potential link between increasing obesity and chronic disease in remote areas with increased take-away options” (Northern Territory 2)*

Underlying the development and implementation of prevention policy were some key political drivers and levers, explored in the next section.

### Key Political Drivers and Levers

The key political drivers identified by study participants included funding, a deregulation agenda, economic growth, and positive perceptions of the government by the public. Levers included creative policy experimentation, positive framing, and community engagement. Some participants noted while external funding from the Commonwealth can enable major investment into obesity prevention initiatives, its withdrawal can damage structural support especially in jurisdictions with less resources.

Key economic drivers, such as funding changes within health departments, influence the approaches taken to achieve long-term outcomes. While having supportive departmental leadership is essential, changes to funding can incapacitate the workforce to deliver policy outcomes, e.g., defunding the health promotion workforces in SA and Queensland (Supplementary Files 2.4, 2.5, GQ D.3.2). Health departments undergo restructuring often which has implications in terms of loss of corporate knowledge and relationships within and beyond the health department. It takes time to build up a community of practice for preventive health work and requires an authorizing environment. While the prevention of obesity was noted as a priority in all jurisdictions health departments, participants described that the level of funding attributed to prevention (compared to “frontline” services) and workforce capacity (e.g., due to restructuring) reflected that it was less urgent than other priorities.

Participants noted the barriers to taking a legislative approach under a broader deregulation agenda:

“*...the whole regulatory impact statement work, is to distill things [each single initiative] down to, ‘Well, what is the evidence that* this *will make a difference?”' (Tasmania) [original emphasis]*

When faced with resistance to regulatory approaches to prevention, departments can be creative in circumnavigating the regulatory framework ideology to normalize health-supportive environments. These include using procurement policies to meet food standards in government-controlled settings and contracts with companies who sell advertising space on public assets to remove discretionary choices advertising—both using legal infrastructure to modify food environments.

In response to this—and their own unique circumstances—Australian states and territories take quite eclectic and occasionally experimental approaches to obesity prevention. Many note that what is missing is providing adequate funding to learn from natural experiments to find “what works” in different contexts:

“*…doing ‘safe-to-fail' experiments… you throw a lot of small amounts of money out to see what comes up from the grass roots and where the strengths are. Then, you can start to play to community strengths… [It's] a more creative approach to [explore] what the mix of interventions that we need might be” (Tasmania)*

There are a lot of different types of evidence used in policy (45), and its use in obesity prevention is complicated. While scientific evidence is valued by policy elites, it is not the only factor taken into consideration and there are evidence gaps about what works best especially for physical activity and food environments. Most health officials are acculturated to think in terms of “evidence-based practice,” which is appropriate for clinical and acute health care needs. However, this study found many participants were changing evidence narratives, referring to “evidence-informed” prevention policy making. This was found to be a more inclusive in considering a broader policy context:

“*…there's a lot of different ways that we describe evidence” (Victoria)*

This approach includes peer-reviewed literature but also respects different forms of evidence, including: community voices, personal and practitioner experiences, informal process evaluation to demonstrate impact of programs, using case studies to develop workforce capacity, international consensus (e.g., the *WHO Ending Childhood Obesity Report*), policy benchmarking (e.g., www.informas.org), and commissioned research/scoping reviews which identify “promising” interventions to make the case for policies aimed at built and food environments. It can also include the experiences of other departments and leveraging off routinely collected data to develop policy and monitoring systems for policy experimentation.

Participants discussed leveraging economic growth aims for health and well-being aims. For example, the concept of “liveability” is emerging as important in the planning sector. It presents an opportunity for a determinants approach to be taken to influence policy decisions about social and affordable housing, public transport and services accessibility. Liveability intersects with smaller jurisdictions seeking to increase their populations (to encourage economic growth) by promoting liveable neighborhoods (Supplementary Files 2.5, 2.6). Food tourism is another area which can be leveraged to progress healthy environments, especially in SA and Tasmania. For example, in Tasmania there is political appetite for supporting tourism, because of its positive impact on the economy. The *Eat Well Tasmania* campaign has leveraged off this appetite to engage with primary producers and retailers to develop Tasmania's local food culture (Supplementary File 2.6). Additionally, they have worked with training institutes to build the capacity of the food service work force, impacting on the local economy, and making healthy affordable food available locally:

“*There is quite an interest that is evolving with the food culture thing, at a whole government level. … because tourism is a major economic driver, but if you make it available for tourists, you're also going make it available for the local community. We're trying to intersect with the tourism sector… [and] the primary producers” (Tasmania)*

An identified barrier to successfully make the case for investments for long-term population level interventions, is the political driver to demonstrate policy success within short political/election cycles. While some jurisdictions identified policymakers are beginning to see the political value of investing in long-term strategies, that prevention is a “marathon not a sprint” (ACT), there are many barriers to securing ongoing support and keeping prevention on the agenda:

“*I think some politicians recognize there may be votes in being committed to longer term agendas” (Tasmania)*

Participants noted a political preference toward immediacy (being able to show what actions are being taken now), over longer-term actions such as legislative changes. This preference for something visible and fast can override the value of evidence:

“*The experience of just making something happen fast and for it to be visible, can preference what is evidenced-based practice” (Queensland)*

This preference for visibility reflects a culture where policies and programs aimed directly at families as recipients are perceived by policy makers as having a higher value than policies aimed at addressing determinants of health and the food and physical activity environments. This culture is influenced by political leanings:

“*A political environment can influence how much is focused on individual responsibility, versus more community collaborative collective impact.” (Tasmania)*

Decision makers are influenced by a range of factors relating to personal and political party ideology, and perceptions of public value. Senior officials respond to their ministers' needs which are influenced by industry, stakeholder, and community group representatives who speak to the interests and portfolios of politicians. Having a strong mandate (e.g., NSW Premier's Priority) represents an opportunity to influence ministers across multiple sectors, however, it is limited by the ideological constraints of “personal responsibility,” a deregulation agenda, and economic growth. The presence of economic rationalism is strong on both sides of politics, and presenting a business case for prevention across a system is trickier than tapping into lesser interests of ministers:

“*…politicians of the day have particular issues that they are specifically interested in, perhaps because stakeholder groups have come in and spoken about it, or they've heard it through their interactions with the Victorian community” (Victoria)*

The potential influence policy makers may lay in approaches to engagement with the public. Study participants had divergent views about the way obesity prevention is/should be portrayed to the public. Some cited concerns over the consequences of stigma relating to public health messages, relying too heavily on telling people *what* to do (rather than *how* to), or the use of non-health settings (such as schools) to monitor childhood obesity prevalence:

“*Obviously, we need to be able to track trends in obesity over time… tick yes, that needs to happen. Is it about weighing every child in school? I'm not sure. Then, how do you manage that feedback to the parents… in a way that's sensitive and appropriate?” (Tasmania)*

“*I think it's far more about having something that people can understand and engage with. When you start talking about physical activity or sedentary behavior or the nutrition environment, [people] will immediately switch off and think that it's like the nanny state” (Australian Capital Territory)*

Some jurisdictions identified problems with historical approaches taken by their own departments, such as an overt focus on obesity, rather than its causes. Those participants willing to learn from past misjudgements emphasized the need for public engagement to focus on environmental causes and desired outcomes, such as well-being or social connectedness, to overcome the potential stigmatizing impacts of obesity policy:

“*We used the word obesity and that was wrong… I think labeling is really important and not creating a stigma around that. Because we know in South Australia that people in our poorest communities are... you know, over 40% of the population of poor suburbs are big compared to 20% in our wealthiest suburbs. So ‘being obese' is normalized in that community… And they're not necessarily in control of that” (South Australia)*

## Discussion

This study provided a snapshot of obesity prevention policies which impact on the first 2,000 days across Australian jurisdictions. It found that no Australian state or territory had policies in place against all the guiding questions, derived from international consensus on actions for the prevention of obesity in childhood. It also found eclectic policy practices between the jurisdictions, influenced by the unique local contexts in each jurisdiction.

### Support Services, Early Childhood Settings and Environments

#### Health Services/Settings

Standalone obesity prevention programs for pre-conception, during pregnancy, or supporting parents of young children were limited across Australian jurisdictions. The only two guiding questions where all jurisdictions had policies in place—antenatal care and universal child health checks—were also two areas with clear national guidelines (46, 47), suggesting the utility of national policy frameworks.

There are opportunities to extend the support offered in community health settings or telephone-based services to include health promotion messages aimed at obesity prevention. However, the contextual option for such programs is likely to sit with upskilling an existing workforce such as those within universal well child programs and child and family services. Within that option, the maldistribution of the health workforce between urban and rural settings—and its association with poorer health outcomes—needs to be addressed to ensure equity (48). Studies have shown workforce interest in obesity prevention (49), including rural communities (50), but health promotion workforce investment needs to be sustained alongside strong policy infrastructure such as that for the *Key Ages & Stages* program in Victoria. Alternatively, states/territories can tap into existing third party provided telephone-based programs (such as the *Get Healthy* suite of programs). Proportionate or progressive universalism applied to healthcare services is likely to benefit those experiencing deprivation the most (51).

#### Early Childhood Education and Care Settings

Three jurisdictions had programs to improve the food and physical activity environments and curriculum in ECEC settings. An umbrella review (52) on the characteristics of successful ECEC interventions for nutrition found that ECEC staff need external support to achieve and maintain healthy eating initiatives. Successful interventions were multi-component (i.e., nutrition, physical activity, child development, etc.), multi-strategy (e.g., educator training to increase skills and knowledge, educator feeding styles, menu planning, positive feeding environments, policy support, etc.) and included parents (52).

While ECEC settings are a key setting for child development and equitable health outcomes (53), educators face high burn out due to workload and limited remuneration (54). Those seeking to promote health within ECEC settings should understand the different roles of those within the sector (e.g., center directors, educators, cooks, etc.). Furthermore, national or statewide policies that seek healthy food provision in ECEC settings must also be supported by policy infrastructure for equitable food access, especially in regional and remote communities. There may be some economies of scale gained from the national harmonization of health promotion policies to support the sector, such as harmonizing state/territory nutrition guidelines which are currently not aligned across Australia (10).

Studies in Australia (55) and the UK (51, 56) confirm interventions in ECEC settings are effective in preventing obesity in the early years especially when partnered with broader community capacity building focused on children in socially deprived areas. However, as with prevention programs aimed at families (14), these too have “fade out” effects in later childhood (51).

#### Environments

The ACT and Victoria had the highest coverage of policy infrastructure for health supportive environments. Australian overweight and obesity data from the 2017–18 Census reflects that Victoria has the lowest prevalence of childhood obesity in Australia, this had decreased since the previous Census (2014–15) (57). Research data from a Victorian community obesity prevention program, *Romp and Chomp*, showed the effectiveness of community-wide interventions in preventing obesity in the early years (55). Data from the same Census shows that ACT residents (adults) are healthier than other Australians (58). However, it must be noted that the ACT population is generally more advantaged, the jurisdiction is geographically small with a budget less constrained by the disadvantages of population dispersal than other jurisdictions, and the ACT government is enabled to undertake both territory and LG functions. Those features make the ACT public sector quite agile compared to the other states and territory.

Environments are physical sites where systems of power (racism, sexism, capitalism and inequality) are exchanged from society to the individual/families (53, 59). Environmental policies improve the social/individual interface to compensate for inequality, they can exhibit significant cost savings in the mid-to-long term (13), and they can eventually change the norm (60). Such interventions are likely to impact on the wider determinants of obesity across the life cycle and would support any intervention aimed at the family or ECEC settings level.

Two interrelated neoliberal political drivers impacted on the likelihood of policies trying to change food or physical activity environments. Policies perceived to impact on personal freedom, e.g., the removal of sugary drinks from government settings, are bound up within public sector reform to reduce the impact of regulation—the “deregulation agenda” (61). Often referred to as “nanny state” in media discourse (62), implementing regulatory measures require the development of a business case assessed through the national Best Practice Regulation framework. The driver to avoid appearing to be a “nanny state” acts as an ongoing constraint in this policy space and has been noted in other studies (63, 64). This study found several examples of jurisdictions using legal frameworks (procurement policies and contractual agreements), but not regulation, to improve health supportive environments—thus circumventing the deregulation agenda. For those processes to succeed, ministerial support (including beyond health) was required.

A focus on growing the economy was the second political driver. While this driver can act as a constraint in this policy space, i.e., it is difficult to progress policies which can be argued as posing a risk to the economy or jobs, it also represents an opportunity. The increased attention to policies which impact on “liveability” and “wellbeing” are linked to efforts to make an area seem desirable to live and encourage population growth. Many jurisdictions are leveraging these terms (rather than “health” or “obesity”) to partner with multiple agencies across government (e.g., planning, communities, environment, transport, and economic development) in addition to LGs and the private sector to progress healthy environments. The co-benefits for liveability with public health, social inclusion, environmental sustainability and the economy “are now well recognized by urban policymakers internationally” (p.1) (65). Currently, although many planning policies aspire toward “liveability” the reality is they are not being implemented. A recent study found that despite the “policy rhetoric championing urban liveability” (p.11) (65) no capital city in Australia performed well on the domains underlying healthy, liveable neighborhoods. As such environments continue to be a space requiring more leadership in Australia.

### Local Health Promotion Models

The findings of this study show that both LG and LHN HPMs can enable programs and initiatives for the early prevention of obesity in childhood. For example, NSW and Victoria—LHN and LG HPM, respectively—are very similar in their settings-based approaches. They both had ECEC programs and both states had invested in large trials for obesity prevention programs in the early years with additional national research funding support. At the time of writing, both states were working on strategies to scale-up these interventions into existing services state-wide.

Additionally, this study found that jurisdictions with the LG HPM were more likely to have policies for health supportive environments. In Australia, LGs have been identified as a key target for action as “they manage many settings where children congregate” (p.356) (66) as well as local planning considerations for health, e.g., enabling employment opportunities, food access and walkability. The findings of this study suggest the LG HPM may have more capacity to engage with environments at the local level than seen in the LHN HPM. As their primary purpose is to deliver health services, LHNs may have trouble divorcing from a (service delivery or) hospital-centric point of view.

Given the division on power between federal, state and LGs in Australia there are constraints on local governance powers, which sit within their state/territory legislative framework—they are “creatures of the states” with no constitutional autonomy (65). The main independent source of revenue for LGs are property/business owner rates, user charges, and fines (67). A study found these constraints heavily impeded NSW LGs ability to implement international recommendations for nutrition interventions at the local level (67). Investment by some jurisdictions in systems approaches at the local level in Australia (68) are subject to the overarching strategies of the state government of the day. There may be multiple political cycles where statewide prevention strategies “miss the mark” to enable systems approaches at the local level.

The *Public Health Act 2016 (WA)* is contributing to LGs higher involvement in obesity prevention activities, although there is evidence that LGs have been participating in such activities for over a decade in WA (66), and many Sydney LGs (in NSW) also participate in health promotion policies with no overarching central government structural framework (67), indicating that LGs are interested in health promotion activities. A study investigating Victorian LGs experiences with health promotion found they held a stronger affinity with addressing the social determinants of health (enabled in the Victorian public health Act) than with aligning to the state priorities within the *Victorian Public Health and Wellbeing Plan* (69). This indicates legislative elements of public health or planning Acts may galvanize LGs in the prevention space more so than statewide strategic policies.

We can look to other countries to learn lessons for LG involvement in prevention initiatives. In 2013 the UK transferred the responsibility for public health from the National Health Service to local authorities (local government), taking a clinical mindset with it (70). This included mandatory and non-mandatory services such as obesity prevention. Since the adoption of austerity measures from 2010, local authorities have faced significant funding cuts, forcing the prioritization of statutory functions and trade-offs between non-statutory services (56). A recent natural policy experiment found that these funding cuts were incrementally correlated to increases in obesity among children at school entry, where the *Sure Start* program (community based early years health service with additional supportive links to childcare and employment/income for parents) had been defunded as a result of these austerity measures (56).

This suggests that policy should shift toward the constraints on LGs as there may be validity in decentralization. However, there is a risk that requiring LGs to participate in health promotion, including strategies aimed at improving determinants, could “bring with it a cost-shifting, or even legitimization of state or national governments' divestment of some of their responsibility for public health” (p.86) (69). Instead, Australia needs long-term commitment to prevention and investment for the wider determinants of health at all levels of government.

### Systems Approaches

Whole of government approaches represent an opportunity to overcome the siloed nature of government agencies. Leadership is required to declare priorities, establish cross-government meetings, and provide the imprimatur to continue. Study participants felt these structures are important, but also sought organizational commitment through maintaining the presence of the same people over time to develop cohesive relationships. The ACT and SA experiences were the accumulation of a range of supportive structural factors that embedded cooperation across sectors and into a range of public sector workforce practices. The success of working across government was tied to (horizontal) collaborative approaches and shaking off “health imperialist” approaches of the past. Policy harmonization can reduce the barriers to collaboration with other sectors by providing top-down (vertical) signaling from leadership as well as structural support.

Although language around partnerships and equity featured in most jurisdictions' key preventive health documents, when it came to specific initiatives to address these areas there was limited policy infrastructure or policy scaffolding to build upon. Three interrelated concepts may help to explain these findings, “short-termism” (70), “lifestyle drift” (59, 70), and “implementation deficit” (71). Policymakers face many competing interests, and the temptation to follow the path of least resistance (22), coupled with the desirability of showing actions and outcomes in the short term are strong incentives for policymakers to focus on lifestyle factors (70).

However, this only explains some of the gap between identified causes of obesity and the implementation of actions. Implementation deficit is the phenomenon whereby the intent of a government is expressed in their policies, however actions to that end are not carried out (71). Lifestyle drift is a phenomenon whereby there is a shifting from interventions aimed at determinants onto individual/family behavior using language such as “empowerment” and “choice” (59). Neoliberal modes of governance inherently reconfigure the responsibility for health and well-being at the feet of the individual (59), which extends to parents in the case of young children. This is not to dismiss the utility of interventions aimed at individual/family lifestyle behaviors (6, 72, 73) or in ECEC settings (52), rather it is to highlight the need to also address wider determinants concurrently (13, 70, 74). Mixed in with lifestyle drift is another phenomenon known as “policy invisibility” (75). As policies move away from families or key settings toward determinants, they lose their visibility. However, they can be made apparent through resource allocation, identifying material impact, and acceptability (public reaction) (75). Interventions are urgently needed from all levels of government and across the public health spectrum (23) between the family, the environments in which they live, and the broader social (76) and commercial (63) determinants of health. Achieving this requires government commitment including the design of governance for implementation by agencies fit for purpose. Partnering with specialized non-government organizations can be beneficial, such as having a specialized workforce, established community relationships, and the ability to be more flexible and meet local community needs. However, these organizations rely heavily on government funding so their workforce is susceptible to the same economic shocks as health departments. Outsourcing what is essentially a government service (i.e., delivering community programs and policies) slowly erodes the responsibility and accountability of government, key features of neoliberalism and short-termism.

### Eclecticism and Collaboration to Find the Way Forward

Multiple jurisdictions referred to their processes as “HiAP by stealth” reflecting a key element of eclecticism across Australian jurisdictions—the way a solution or process is represented in one jurisdiction is often not organisationally compatible in another. While the same or similar concepts can (and have) been taken up across jurisdictions, they first need to be re-packaged to fit into ministerial priorities and use language and jargon that makes sense in the local context. Eclecticism in health care can be associated with negative outcomes (77), but it is not inherently responsible for policy gaps. A systems approach to address obesity will never have a “one size fits all” solution and eclectic practices across jurisdictions help to resist oversimplifying the complexity of the ongoing nature of obesity prevention. An opportunity exists to position systems thinking at the forefront of obesity prevention, without rejecting everything that has come before it (21).

In the UK, guidance for LGs notes that they “should not feel constrained to implement only interventions with evidence of effectiveness. The evidence base to tackle this serious issue will only improve if areas try new interventions and then evaluate them” (p.63) (78). The utility of natural experiments has been identified in the literature as an emerging area to generate evidence for population health interventions aimed at health-supportive environments (79). HiAP was a policy experiment, and it took leadership to permit a shift in the ways sectors collaborated for preventive health in SA.

Taking an eclectic approach allows for innovation within and between states but what is needed are better ways for jurisdictions to learn from each other, to share, adapt, and scale up initiatives so they are contextually relevant but still have some consistency across jurisdictions. To achieve this requires a mechanism to facilitate sharing, learning, and adaptation to context. Previous examples of such mechanisms could be found in the NPAPH and Australian National Prevention Health Agency for intergovernmental exchange, and the ACT *Healthy Weight Action Plan* developed good mechanisms for working across government.

The lack of national funding widens disparity between jurisdictions as only those jurisdictions with enough internal funding can continue to provide health promotion interventions if national funding is revoked. Calls for a renewed national prevention agency and reinvestment in health promotion are not new (7, 80). However, this study identified some key elements that such an agency could provide. An agency that facilitates the sharing of ideas and practice-based knowledge across all levels of government, including LGs (67). It could provide a mechanism for policy makers and practitioners to link up with the right part(s) of agencies within and beyond health who share contextually similar circumstances (e.g., rural vs. urban or LG vs. LHN HPMs). It could shoulder the administrative burden that acts as a barrier to collaboration for public servants between jurisdictions, and it could fund “safe-to-fail” natural experiments and use easy and cost-effective measurements to ascertain the elements of efficacy that are context specific and how they can be adapted across multiple Australian contexts. Creating such a learning policy environment allows for the curiosity and experimentation needed to answer “what works” and “for who” with obesity prevention (81).

### Strengths and Limitations

To our knowledge, this is the first study to compare policies for the early prevention of obesity across Australian states and territories. The guiding questions used in the policy mapping tool can monitor progress in policy development over time. It must be restated that the tool did not quantify the effectiveness of found policies, a limitation that extends to this study generally. This study sought to capture some of the complexity of obesity prevention policy between Australian state and territory governments, from the perspectives of senior health officials, and it contributes to the limited empirical evidence of “lifestyle drift” [see also (70)]. This study did not seek out the perspectives from senior officials in other relevant departments, as the primary aim was to consider the complexity of obesity policy between jurisdictions, not within a single jurisdiction. However, future research should consider exploring the complexity of obesity prevention policy within a single jurisdiction across more sectors with policy responsibility (or opportunity to implement policies) for obesity prevention.

## Conclusions

The first 2,000 days is a critical period in which to intervene for establishing lifelong habits. However, without concurrent intervention in environments and the wider determinants of health, the positive effects of interventions in family and ECEC settings are likely to “fade out” and therefore not maximize their potential impact across the life cycle. Even within a single country, obesity prevention policy needs to be adaptable to local contexts. The eclecticism undertaken by Australian states and territories provides opportunities to share, learn, and adapt their experiences within and between jurisdictions (at all levels of government) but need funding and structural support to do so.

This study found that senior health officials worked within a neoliberal paradigm. This often resulted in an implementation deficit between the identified causes of obesity in overarching strategic frameworks and the interventions/programs that flow from them. The global disruption of COVID-19 presents an “important window of opportunity to collectively change the system such that communities are able to live with good health, dignity and in an environmentally sustainable way” (53). Eclectic governance structures enable diverse policy responses in Australia. While eclecticism captures this diversity, a national prevention agency has the potential to create a decentralized, innovative, and experimental learning policy environment to enable learning within and between jurisdictions.

## Data Availability Statement

The original contributions presented in the study are included in the article/Supplementary Material, further inquiries can be directed to the corresponding author.

## Ethics Statement

This study was approved by the University of Sydney Human Research Ethics Committee (2017/507). The participants provided written informed consent to participate in this study.

## Author Contributions

EE developed the study design, undertook policy mapping and analysis (cross-referenced by LW and CR), conducted the interviews with senior health officials and undertook the analysis (reviewed by LW and CR), and wrote the manuscript. All authors contributed to the final manuscript.

## Funding

EE was a recipient of scholarships from the Australian Government Research Training Program and the NHMRC Centre of Research Excellence for the Early Prevention of Obesity in Childhood (grant number 1101675).

## Conflict of Interest

The authors declare that the research was conducted in the absence of any commercial or financial relationships that could be construed as a potential conflict of interest.

## Publisher's Note

All claims expressed in this article are solely those of the authors and do not necessarily represent those of their affiliated organizations, or those of the publisher, the editors and the reviewers. Any product that may be evaluated in this article, or claim that may be made by its manufacturer, is not guaranteed or endorsed by the publisher.

## Abbreviations

ACT: Australian Capital Territory
NSW: New South Wales
NT: Northern Territory
SA: South Australia
WA: Western Australia
ACECQA: Australian Children's Education & Care Quality Authority
ECEC: Early childhood education and care
GQ: Guiding question
HPM: Health Promotion Model
LG: Local government
LHN: Local Hospital Network
NPAPH: National Partnership Agreement on Preventive Health
WHO: World Health Organisation.
