# Supplementary material for: Australian State and Territory Eclectic Approaches to Obesity Prevention in the Early Years: Policy Mapping and Perspectives of Senior Health Officials
Source: Front Public Health. 2022 Jun 3;10:781801. doi: 10.3389/fpubh.2022.781801 (PMC9204007; doi:10.3389/fpubh.2022.781801)
Supplement: Supplementary file 1 [file Data_Sheet_1.PDF]

Supplementary File 1: Supplementary material for introduction and methods

1.1 Table: Key demographic and socioeconomic indicators by state and territory 2

1.2 Policy mapping tool adaption from the *WHO Ending Childhood Obesity* report 4

1.2.1 Example of adapting *WHO Ending Childhood Obesity Report* recommendations for the Australian state/territory context..7

1.2.2 Summary of the WHO Ending Childhood Obesity Report sections 7

1.2.3 Examples of policy areas beyond individual state/territory jurisdiction in Australia................8

1.3 Semi-structured interview guide 9

1.1 Table: Key demographic and socioeconomic indicators by state and territory

**2016 – 2018**	**ACT**	**NSW**	**NT**	**Qld**	**SA**	**Tas**	**Vic**	**WA**
Population (1)								
Total	397,397	7,480,228	228,833	4,703,193	1,676,653	509,965	5,926,624	2,747,410
Aboriginal and/or Torres Strait Islander (% of jurisdiction)	6508 (1.6%)	216,176 (2.9%)	58,248 (25.5%)	186,482 (4.0%)	34,184 (2.0%)	23,572 (4.6%)	47,788 (0.8%)	75,978 (3.1%)
Proportion of population by Index of Relative Socio-economic Advantage and Disadvantage (SEIFA IRSAD)(1)*								
Quintile 1: most disadvantaged	0.7%	19.1%	23.5%	21.4%	25.7%	37.2%	16.2%	13.3%
Quintile 2	3.5%	18.8%	11.1%	22.2%	25.1%	26.1%	18.0%	18.4%
Quintile 3	12.1%	17.1%	19.8%	21.7%	22.4%	18.3%	21.4%	22.9%
Quintile 4	29.1%	17.4%	25.2%	20.7%	17.9%	13.8%	23.7%	24.0%
Quintile 5: most advantaged	54.6%	27.6%	20.5%	14.0%	8.9%	4.6%	20.8%	21.4%
Population dispersal % (2)								
Major City	99.8	75.3	-	63.8	73.5	-	77.8	78.1
Inner Regional Area	<1	18.7	-	19.7	12.8	67.8	18.3	8.7
Outer Regional Area	-	5.6	60.2	14.0	10.3	30.3	3.9	7.2
Remote	-	<1	19.5	1.5	2.6	1.5	<1	3.3
Very Remote	-	<1	20.3	1.1	<1	<1	-	2.7
Land size (km^2) (3)	2,358	800,811	1,348,094	1,730,172	984,275	68,018	227,496	2,526,646
Population density 2018 (persons/km^2) (3)	178.5	10.0	0.2	2.9	1.8	7.8	28.4	1.0
Budget financial year 2018-19 ($billion) (4-11)	$5.82	$79.66	$6.89	$56.70	$19.53	$6.06	$62.96	$30.50
Local Hospital Networks (or equivalent)	no additional	15 Local Health Districts	2 Local Hospital Networks	16 Hospital & Health Services	10 Local Health Networks	4 Tasmanian Health Organisations	not networked (86 distinct entities)	6 Health Service Providers
Number of Local Governments	-	128	17	77	68	29	79	152

*Across Australia, almost half (48%) of the people who identify as Aboriginal and/or Torres Strait Islander live in areas of most disadvantage, compared to 18% non-Indigenous Australians. Further, while 22% of non-Indigenous Australians live in areas of most advantage, only 5% of people who identify as Aboriginal and/or Torres Strait Islander do (1).

References

1. Australian Bureau of Statistics. Data from: Census of population and housing: Reflecting Australia - stories from the Census, 2016. cat. no. 2071.0. (2017) https://www.abs.gov.au/ausstats/abs@.nsf/Lookup/by%20Subject/2071.0~2016~Main%20Features~Snapshot%20of%20Australia,%202016~2

2. Australian Bureau of Statistics. Data from: Regional population growth, Australia 2016-17. cat. no. 3218.0. (2018) https://www.abs.gov.au/AUSSTATS/abs@.nsf/DetailsPage/3218.02016-17?OpenDocument

3. Australian Bureau of Statistics. Data from: Regional population growth, Australia, 2017-18. cat. no. 3218.0. (2019) https://www.abs.gov.au/AUSSTATS/abs@.nsf/DetailsPage/3218.02017-18?OpenDocument

4. Australian Capital Territory Government. Budget Paper 3: Budget Outlook. Canberra: ACT Government; 2018.

5. New South Wales. Budget Paper No.1. Sydney: NSW Government; 2018.

6. Northern Territory. Budget Paper No. 2: Budget Strategy and Outlook 2018-19. Darwin: NT Government; 2018.

7. Queensland Government. State Budget 2018-19 Budget Strategy and Outlook: Budget Paper No. 2. Brisbane: Queensland Government; 2018.

8. South Australian Government. State Budget 2018-19 Budget Statement Budget Paper 3. Adelaide: SA Government; 2018.

9. Tasmanian Treasury. The Budget: Budget Paper No. 1. Hobart: Tasmanian Government; 2018.

10. Victorian Government. Victorian Budget 18/19 Getting Things Done. Melbourne: Victorian Government; 2018.

11. Western Australian Government. 2018-19 Budget Economic and Fiscal Outlook: Budget Paper No. 3. Perth: WA Government; 2018.

1.2 Policy mapping tool adaption from the *WHO Ending Childhood Obesity* report

Area	Guiding questions	WHO Ending Childhood Obesity Implementation Plan
**A. Governance & leadership**		
A.1 Leadership	A.1.1 Has childhood obesity prevention been identified as a priority by leadership (Premier/First Minister or Health Minister)?	Leadership section, A. Leadership
	A.1.2 Key policy: Is there an overarching policy framework, or a series of key policies or action plans to guide initiatives for the early prevention of obesity in childhood?	Leadership section, A. Leadership and D. Good Governance
	A.1.3 Does the state/territory legislation for public health include prevention/health and wellbeing?	Leadership section, A. Leadership
	A.1.4 Are their statutory grant-giving bodies with a remit to fund prevention-related community projects?	Leadership section, A. Leadership
A.2 Partnerships	A.2.1 Are partnerships across government noted in 'key policy' identified above?	Leadership section, A. Leadership, B. Joint Action, D. Good Governance
	A.2.2 Are there formal mechanisms for collaborative exchange across sectors (e.g. working groups, policy/outcome joint statements, embedded health positions in agencies outside of health)?	Leadership section, A. Leadership, B. Joint Action, D. Good Governance
A.3 Equity	A.3.1 Do the key policies identified outline the structural (incl. social/commercial) causes of obesity? (such as employment/family income, affordable or social housing, adverse early childhood experiences, food security, food systems including promotion, built environment and access to safe/appropriate spaces for being active, etc)	Leadership section, B. Joint Action, Actions 1.9(a-c). Previous research identified that the *WHO Ending Childhood Obesity* report focused more heavily on food and physical activity environments than other determinants of health (Link) so equity guiding questions have been added here based on elements identified in WHO *Closing the Gap in a Generation: Health Equity through Action on the Social determinants of health* (Link).
	- A.3.1a Do recommendations for action/initiatives address these structural causes?	
	A.3.2 Are target populations (with higher risk of developing obesity) identified for additional support?	
**B. Environments in which we live (e.g. work, shop, eat, be active and play)**		
B.1 Health supportive environments	B.1.1 Do planning policies orientate built environments towards principles of active living?	Leadership section, B. Joint Action Actions 2, Actions 2.2
	B.1.2 Are there investments for public infrastructure (e.g. footpaths or bikeways) to encourage being active?	Leadership section, A. Leadership and B. Joint Action, Action 2, Actions 2.2
	B.1.3 Are there food/nutrition policies aimed at ensuring a nutritious, affordable, accessible food system? (e.g. incentivise local food production or increase healthy food access in disadvantaged communities, zoning policies, or incentives to retailers)	Leadership section, B. Joint Action Action 1, Action 1.9 (a-c)
	B.1.4 Are there programs to support vendors to improve food offerings in food outlets (restaurants, cafes, take-away, vending machines)?	Leadership section, D. Good Governance, Action 1.1

	B.1.5 Is nutrition information at food outlets (menu board labelling) required by legislation?	Leadership section, D. Good Governance, Action 1.1
	B.1.6 Is there engagement with food retail (supermarkets, grocers, corner stores, etc) to reduce the availability and promotion of discretionary choices in-store?	Leadership section, D. Good Governance, Action 1.1, Actions 4.5
	B.1.7 Are local governments empowered to encourage health-supportive environments?	Leadership section, B. Joint Action and D. Good Governance, Action 1.9
	B.1.8 Are there any initiatives to reduce exposure to the **marketing/promotion** of discretionary choices in:	
	- B.1.8a out-of-home advertising (billboards, transport vehicles, street furniture, transport hubs such as train stations) within government control?	Leadership section, B. Joint Action Action 1.1, Actions 1.3(a-c) (see Supplementary File 1.1.1 for more information)
	- B.1.8b healthcare settings?	
	- B.1.8c other government-controlled buildings/parks?	
	B.1.9 Are there policies limiting the **availability/provision** of discretionary choices in:	
	- B.1.9a healthcare settings (for visitors and staff)?	Leadership section, B. Joint Action Action 1.1, Actions 1.8(a-b)
	- B.1.9b buildings, community centres, and parks under government control?	
B.2 Health promotion campaigns	B.2.1 Are there health promotion campaigns aimed at encouraging healthy lifestyle behaviours?	Action 1.1d, Actions 2.1(a-d) Action 4.13 (a-b), Actions 4.3
	B.2.2 Are there health promotion campaigns aimed at developing/supporting healthy food systems and built environments (incl. community-capacity building)?	Action 4.13(c-d)
**C. Early childhood education and care (ECEC) settings**		
C.1 ECEC settings	C.1.1 Are there support programs for centre-based care settings to encourage healthy food provision? (e.g. management: policies and menu audits; staff: training and resources; families: resources)	Action 4.9, Action 1.8b, Actions 4.13
	C.1.2 Are there programs to support provision of food and physical activity experiences as part of the curriculum?	Actions 4.12, Action 4.11b
**D. Health (community and tertiary health settings and health promotion activities)**		
D.1 Antenatal and birth services	D.1.1 Does antenatal care screen and manage hypertension, hyperglycaemia, appropriate gestational weight gain?	Actions 3.1, Actions 3.2
	D.1.2 Antenatal care within public health services:	
	- D.1.2a Do they include nutrition counselling for healthy pregnancy or are there other healthy lifestyle support programs available during pregnancy?	Actions 3.3, Actions 3.4(a-c)
	- D.1.2b Is breastfeeding education free (separate or embedded into antenatal education/services)?	Action 3.4c, Actions 4.3
	D.1.3 Do maternity facilities fully adhere to the Baby Friendly Health Initiative (based on *Ten Steps to Successful Breastfeeding*)?	Actions 4.2
D.2 Early childhood services	D.2.1 Are there free health/parenting services to support early childhood growth/nutrition (e.g. breastfeeding, complementary feeding, transition to family foods)? (universal health check)	Actions 4.8(a-b), Action 4.3
	- D.2.1a Is information to support parents readily available (e.g. phonelines, websites)?	
	- D.2.1b Do these include breastfeeding support?	

	D.2.2 Are there healthy lifestyle (education) programs to support families during early childhood? (statewide healthy lifestyle program)	Actions 4.8(a-b)
	- D.2.2a Are target populations identified and actively recruited for programs?	
	D.2.3 Are Supported Playgroups offered for families that need additional support and do they include healthy lifestyle skills? (targeted support programs, available across the state)	Actions 4.8(a-b)<br>(see also GQs A.3)
D.3 Workforce	D.3.1 Are there training and resources available for health care professionals to support families?	Leadership section, A. Leadership<br>Actions 2.1
	- D.3.1a Is preconception advice for nutrition and being active provided to prospective parents?	Actions 3.4(a-c)
	D.3.2 Is there a state/territory health promotion…	
	- D.3.2a …agency (independent or adjunct to health department)?	Leadership section, A. Leadership<br>Also includes public health monitoring information. Leadership section, C. Data for Action.
	- D.3.2b …workforce (to implement initiatives locally)?	

1.2.1 Example of adapting *WHO Ending Childhood Obesity Report* recommendations for the Australian state/territory context

A recommendation of the *WHO Ending Childhood Obesity Report* is to restrict discretionary choices marketing/advertising (Actions 1.3), according to the *WHO Set of Recommendations on the Marketing of Foods and Non-Alcoholic Beverages to Children* (Link). In Australia, the responsibility for broadcast and online media regulation sits with the Commonwealth Government. Beyond broadcast and online media there are other options to reduce the impact of advertising to children. In Australia an example of that is the intergovernmental work within the Council of Australian Governments Health Council to reduce the effects of marketing of discretionary choices to children across key settings, in partnership with health, education, and sport/recreation sectors. To adapt to the state/territory level, consideration was given to sites of publicly available advertising which state/territory governments have policy control. These include government assets (e.g. food outlets and signage within buildings) and out-of-home advertising assets such as public transport vehicles, street furniture and transport corridor signage, which have been included in the policy mapping tool (see guiding question B.1.8).

1.2.2 Summary of the WHO Ending Childhood Obesity Report sections

**Leadership section**	A. Leadership
	B. Joint Action
	C. Data for action
	D. Good Governance
**Actions**	1. Implement comprehensive programmes that promote the intake of healthy foods and reduce the intake of unhealthy foods and sugar-sweetened beverages
	2. Implement comprehensive programmes that promote physical activity and reduce sedentary behaviours in children and adolescents
	3. Integrate and strengthen guidance for noncommunicable disease prevention with current guidance for preconception and antenatal care, to reduce the risk of childhood obesity
	4. Provide guidance on, and support for, healthy diet, sleep and physical activity in early childhood to ensure children grow appropriately and develop healthy habits
	5. Implement comprehensive programmes that promote healthy school environments, health and nutrition literacy and physical activity among school aged children and adolescents [not applicable to this study as is focused on school aged children, not the first 2000 days]
	6. Provide family-based, multicomponent services on lifestyle weight management for children and young people who are obese [not applicable to this study as is focused on obesity treatment, not prevention]

Citation: World Health Organisation (WHO). Report of the Commission on Ending Childhood Obesity. Implementation plan: executive summary. Geneva: WHO; 2017 (WHO/NMH/PND/ECHO/17.1). Available at: https://apps.who.int/iris/bitstream/handle/10665/259349/WHO-NMH-PND-ECHO-17.1-eng.pdf

1.2.3 Examples of policy areas beyond individual state/territory jurisdiction in Australia

**Area**	**Examples**	**Responsibility**
Governance	National obesity and/or prevention strategy<br>National nutrition strategy<br>National physical activity strategy<br>& funding for initiatives to be implemented at the state/territory level	Commonwealth/ intergovernmental
Health supportive environments	Unemployment benefits and other social safety nets	Commonwealth
	Workplace rights (parental leave, breastfeeding in workplace legislation, flexible working arrangements)	Commonwealth
	Regulation on the marketing of discretionary choices (broadcast/online/digital media)	Commonwealth
	Regulation on the marketing of breast milk substitutes and commercially available foods aimed at infants and young children	Commonwealth
	Food system regulation (Food Standards Australia New Zealand)<br>- front of pack labelling<br>- nutrition information panel<br>- ingredient lists<br>- food and beverage composition requirements	Intergovernmental
	Fiscal levers available at the national level:<br>- Tax or levy on unhealthy foods and/or beverages<br>- Protective exclusions from taxes to encourage healthy eating (e.g. Australian GST does not apply to core foods)<br>- Tax benefits to manufacturers to improve composition of their food products<br>- Increases to social welfare payments to ensure families have sufficient funds to meet the costs of living	Commonwealth
ECEC sector	Authority for the ECEC sector sits nationally with an authorising body the Australian Children's Education & Care Quality Authority (ACECQA). ACECQA manages National law and regulation for the ECEC sector; the National Quality Standard (NQS), national benchmark standards in seven quality areas; the National Quality Framework (NQF), minimum standards for educator qualifications and ratio requirements, legal obligations, and learning frameworks. Under this system state and territory regulatory authorities assess services against these legal obligations.	Commonwealth/ intergovernmental
Health services	National funding for prevention in/beyond health services	Intergovernmental
	General Practitioners and primary healthcare including Primary Health Networks, Medicare scheme, Pharmaceutical Benefits Scheme	Commonwealth

1.3 Semi-structured interview guide

This study is interested in policy for the early prevention of obesity in childhood in your jurisdiction. It seeks to compare Australian state and territory approaches to this policy space and how the different contexts in each jurisdiction affects what becomes the focus of each jurisdiction. As well as policies which relate to the whole of population – such as public information campaigns and broader environmental considerations for being active, accessing healthy food and other social determinants of health – this study would like to highlight key areas for obesity prevention in early childhood (from conception until children enter school).

Policy mapping and health department view of policy landscape

1. Just to start could you please tell me a bit about your role in obesity prevention policy?
2. Is childhood obesity prevention a priority in [jurisdiction]? How do you think the problem is framed in [jurisdiction]?
3. What do you see as the key pieces of policy which drive your departments strategic planning and actions for the early prevention of obesity?
4. Can you describe the services the health department delivers for the early prevention of obesity?

- ***Prompt**: pre-conception, pregnancy, early childhood*

5. [referring to key policies identified in mapping] Can you think of any other frameworks or policies that support obesity prevention work in [jurisdiction]?
6. Can you describe the policy infrastructure for obesity prevention in [jurisdiction]?

- ***Prompt***: Legislation, funding, health promotion agency/workforce

7. Can you describe some of the context for the decision-making processes for obesity prevention policies, from your perspective?

- ***Prompt**: can you give an example/ tell me more about that?*

8. Can you describe the influence and the role of evidence in the development of [jurisdiction's] response to childhood obesity?
9. Can you please reflect on the ways [jurisdiction] is monitoring progress or outcomes?

Now I would like to turn our discussion to thinking about some of the broader environmental considerations of health, such as the social determinants of health, the food system (agriculture, manufacturing, food imports) and the physical or built environment. This includes consideration of other departments or agencies in [jurisdiction], e.g. education, social services, development, planning, transport, local government, treasury, etc.

10. Are you aware of policies beyond the health department that might support or hinder obesity prevention efforts?

- ***Prompt**: Does the health department undertake scans of obesity prevention policy across different government agencies within [jurisdiction]?*
- ***Prompt** this might include physical activity strategy incl. transport/ planning/ design considerations; food strategy incl. settings and broad food environment (education, industry, local government)*

11. Can you comment on some of the ways that health engages with other government agencies to pursue policies outside of the traditional 'health' policy space?

- ***Prompt**: types of collaborative networks and engagement, formal/ informal, etc (e.g., advocacy groups, community organisations, local government)*

12. Can you comment on the role of institutional factors (health department and/or other departments) in the adoption of prevention policies, if at all?

- ***Prompt**: collaboration between departments, explicit government priorities, treasury support (institutional factors have been noted in the literature/ observed in other jurisdictions)*
