# Supplementary material for: Australian State and Territory Eclectic Approaches to Obesity Prevention in the Early Years: Policy Mapping and Perspectives of Senior Health Officials
Source: Front Public Health. 2022 Jun 3;10:781801. doi: 10.3389/fpubh.2022.781801 (PMC9204007; doi:10.3389/fpubh.2022.781801)
Supplement: Supplementary file 2 [file Data_Sheet_2.PDF]

Supplementary File 2: Supplementary materials for results

Results of policy mapping in each state and territory

One page summary of policy mapping 2

Supportive information to interpret policy mapping 3

2.1 Australian Capital Territory 5

2.2 New South Wales 13

2.3 Northern Territory 20

2.4 Queensland mapping 28

2.5 South Australia 37

2.6 Tasmania 47

2.7 Victoria 56

2.8 Western Australia 65

One page summary of policy mapping

The policy mapping found that childhood obesity was identified as a problem in most jurisdictions (Guiding Question (GQ) A.1.1). The key life stages of pregnancy and/or early childhood (or as the first 2000 days) are less well defined in key strategic documents (GQ A.1.2). Having an overarching policy framework or strategy to address obesity/childhood obesity (GQ A.3) did not guarantee action or implementation plans in the areas of health supportive environments, ECEC settings, or health settings. Instead, the language used to describe the causes of obesity and to identify policy action areas were a better indication of policy infrastructure available across these areas. For the most part the initiatives that flowed out from the key strategic frameworks in Areas B-D were focused on increasing skills and knowledge at the family level, whereas the language to describe the structural causes of obesity in the context of policy options was vague, e.g. 'partnerships to improve environments'. However, where clear language was used to identify specific areas (e.g. food advertising) as contributing to obesity in key policy documents, specific policies to address the social determinants of health and health supportive environments were more likely.

Antenatal screening and management of pregnancy-related risk factors for obesity (parent and child) (GQ D.1.1), was one of the few guiding questions where all jurisdictions had policies in place. Materials to support antenatal anticipatory breastfeeding support were available in half of the jurisdictions. Few jurisdictions fully adhered to the *Baby Friendly Health Initiative* (GQ D.1.3). Generally, information and advice for parents in the early years was readily available, although access to additional services to support parents with infant feeding, particularly breastfeeding, was limited. While there were no state-wide healthy lifestyle/prevention programs available for families with children in early childhood (GQ D.2.2), the smaller programs that did exist tended to actively identify target populations for recruitment. The programs that were available at the time of mapping were not yet available as state-wide programs, and implementation research projects were underway to investigate modes for program scalability. Universal child health clinics (GQ D.2.1) were another example of services offered by all jurisdictions, including support for infant and young child feeding and early movement skills. Although there was wide variation in the comprehensiveness of services offered and policy guidelines to support implementation, e.g. the *Key Ages and Stages* universal child health program in Victoria had a comprehensive policy framework. Less than half of the jurisdictions had both a health promotion agency and sufficient workforce to support local implementation of health promotion activities (GQ D.3)

There were very limited health promotion campaigns on any media which focused on the promotion of healthy lifestyles for families and/or for the early years (implicit and explicit health promotion), to encourage supportive environments for breastfeeding, or to build community capacity to promote health supportive environments (GQs B.2). Three jurisdictions had government-funded health promotion programs to support centre-based long-day care in ECEC settings (see GQs C.1). In one program, *Munch & Move* in NSW additional support was provided to the early childhood directorate within the education department, whose authorising officers were required to assess ECEC services against the National Quality Framework. The Victorian programs included the *Achievement Program* (supporting healthy lifestyle related learning and experiences) and the *Healthy Eating Advisory Service* (providing menu audits to centres and support to improve food offerings), both delivered by not-for-profit health promotion organisations. The Tasmanian program was adapted from a former Victorian program. Although some non-government organisations provide resources for the ECEC sector in other jurisdictions for a fee, government-funded programs were more equitable and had better reach.

Most jurisdictions have planning and/or transport policies that link health and wellbeing to the built environment, being physically active and reducing sedentary behaviour, although few provide additional policy tools to local governments to enable health supportive planning decisions (GQs B.1.1, B.1.2, B.1.7). Additionally, policies to improve the food environment in terms of access, provision, and promotion were quite limited, often with no policy at all or very few elements that align to GQs. Health settings are one area where policies to limit the availability of discretionary foods and drinks to visitors and staff are becoming normalised across Australia, and to a lesser extent, policies to reduce exposure to marketing of these products in those settings (GQs B.1.8b, B.1.9a). All but three jurisdictions have enacted requirements for energy (kilojoule) labelling on food outlet menu boards (GQ B.1.5). The policy was being considered by WA at the time of mapping. The smaller jurisdictions of both Tasmania and NT had not acted as major food outlets already adopted applied the policy nationally.

Supportive information to interpret policy mapping

Please note: These tables are not intended to be an absolute mapping of all policies for the early prevention of childhood obesity in Australian states and territories. Rather, it is a compilation of examples of policies and policy levers being used by Australian jurisdictions to support and improve health supportive environments and healthy lifestyle behaviours to prevent obesity. Where appropriate, quotes from study informants have been included, denoted in bold, e.g. (**ACT informant**). More comprehensive mapping on health supportive food (see www.informas.org) and physical activity (Link, Link, Link) environments at the state/territory level can be found elsewhere in the literature.

For items where results were 'Policy void' this indicates that no policies were found for the guiding question. That does not necessarily mean they did not exist, just that no policies were found online at the time of mapping. Conversely, for items where results are 'Policy/initiative found' it does not necessarily mean that those policies are effective, or have even been implemented, just that they exist and align with the guiding questions. Where possible URLs have been included as '(Link)' to provide the reader with more information if they are interested. While every attempt was made to ensure active URLs, some will likely break into the future and cannot be guaranteed to be active (click on URLs at your own discretion). Initial mapping was undertaken between mid-2018 to mid-2019, and URLs were checked and updated prior to paper submission in 2021. This allowed some additional updates – noted as Update 2021 – to be added to the mapping notes but these additional comments did not impact on original results.

If you use the data in this Supplementary File in anyway, ensure you include a reference and an in-text note about the location in Supplementary File 2.

Legend

Colour	
(blue)	Policy/initiative in place
(green)	Policy Infrastructure
(yellow)	Policy Scaffolding
(grey)	Policy Void

**Acronyms and abbreviations – all jurisdictions**			
ACT	Australian Capital Territory	ABS	Australian Bureau of Statistics
NSW	New South Wales	ACM	Australian College of Midwives
NT	Northern Territory	BFHI	Baby Friendly Health Initiative
Qld	Queensland	CATI	Computer Assisted Telephone Interview
SA	South Australia	CBC	Centre-based care
Tas	Tasmania	COAG	Council of Australian Governments
Vic	Victoria	ECEC	Early childhood education and care
WA	Western Australia	MESCH	Maternal Early Childhood sustained nurse Home Visiting Program
		MoU	Memorandum of Understanding
		NGO	Non-Government Organisation
		NHMRC	National Health and Medical Research Council
		NPAPH	National Partnership Agreement for Preventive Health
		NQF	National Quality Framework
		SSBs	Sugar-sweetened beverages
		WHO	World Health Organisation

Acronyms and abbreviations – jurisdiction-specific	
**a) Australian Capital Territory**<br>MACH Maternal and Child Health	**e) South Australia**<br>CaFHS Child & Family Health Service<br>HiAP Health in All Policies<br>OPAL Obesity Prevention and Lifestyle<br>RPHP Regional Public Health Plan<br>SPHP State Public Health Plan
**b) New South Wales**<br>CHAT Communicating Healthy Beginnings Advice by Telephone<br>DPC Department of Premier and Cabinet<br>HCI Healthy Children Initiative<br>HCI Healthy Children Initiative<br>HEAL Healthy Eating and Active Living<br>LHD Local Health District, 15 across the state<br>MoH Ministry of Health<br>OPH Office of Preventive Health	**f) Tasmania**<br>CHaPS Child Health and Parenting Service<br>DHHS Department of Health and Human Services<br>THS Tasmanian Health Service<br>TPPs Tasmanian Planning Policies
**c) Northern Territory**<br>CAHS Central Australia Health Service<br>DHF Department of Health and Families<br>FaFT Families as First Teachers<br>HU5K Healthy Under 5 Kids<br>TEHS Top End Health Service	**g) Victoria**<br>DET Department of Education and Training<br>DHHS Department of Health and Human Services<br>HEAS Healthy Eating Advisory Service<br>KAS Key Ages and Stages<br>MAV Municipal Association of Victoria<br>MCH Maternal and Child Health<br>MPHWP Municipal Public Health and Wellbeing Plan<br>VACCHO Victorian Aboriginal Controlled Community Health Organisation<br>VHEE Victorian Healthy Eating Enterprise<br>VPHWP Victorian Public Health and Wellbeing Plan
**d) Queensland**<br>HHS Hospital and Health Service<br>LEAPS Learning, Eating, Active Play and Sleep<br>NAQ Nutrition Australia Queensland<br>SPP State Planning Policy	**h) Western Australia**<br>SPHP State Public Health Plan<br>LPHP Local Public Health Plan

2.1 Australian Capital Territory

Area	Guiding questions	Result	Notes
**A. Governance & leadership**		**ACT**	
A.1 Leadership	A.1.1 Has childhood obesity prevention been identified as a priority by leadership (Premier/First Minister or Health Minister)?		Yes. Enabling policy environment. The *Healthy Weight Initiative* (2014-2018) was led by the Chief Minister (who was also the Minister for Health) and included several implementation groups, a taskforce, and regular progress reports. Specific health and settings-based opportunities for the first 2000 days were mostly missed, but the upstream approaches undertaken in the initiative still impacted on weight for children under five years – kindergarten children proportion with overweight and obesity went from 16% at baseline 2010-2012 to 15% at 2014 (see Healthy Weight Initiative 2016 progress report Link).
	**A.1.2 Key policy/policies**: Is there an overarching policy framework, or a series of key policies or action plans to guide initiatives for the early prevention of obesity in childhood?		The *Healthy Weight Initiative* was under review at time of interview: next iteration will be called *Healthy and Active Living*, a recognition of moving away from a focus on weight towards a focus on lifestyle (although obesity prevention remains a key pillar).<br>The Healthy Weight Initiative was supported by *Towards Zero Growth: Healthy Weight Action Plan* (Link). This was developed as a whole of government plan with input from ACT directorates (during the interview, ACT participant noted that a journal article has been published on this process Link). At the heart of this process was engaging sectors to see the problem of obesity beyond 'client-centred' approaches (i.e. personal responsibility) towards structural approaches. *Towards Zero Growth* had four work areas identified: food environment, schools, workplaces, urban planning. Implementation teams were established across different agencies, for health and non-health programs and policies.<br>The ACT informant noted that despite the successes of the *Healthy Weight Action Plan*, prevention was not receiving enough funding in general: "There's a lot of rhetoric at all levels of government… about how important prevention is, but it continues to not get anything like the level of funding that… 'Frontline' services seem to get… So when it comes to arguing with colleagues in Treasury as to the extent to which, you know, investment in prevention will offset demand on acute services, for example, that's very challenging" (**ACT informant**).
	A.1.3 Does the territory legislation for public health include prevention/health and wellbeing?		No. The Public Health Act in the ACT does not contain 'well-being' or chronic disease prevention elements. However, health promotion is a key role and statutory function of the Chief Health Officer.
	A.1.4 Are their statutory grant-giving bodies with a remit to fund prevention-related community projects?		Not a statutory body, but an initiative called *Health Promotion Grants* Program ($2.1milion noted in *Towards Zero Growth*, 2014). *Healthy Canberra Grants* funded programs with Oz Harvest and the Australian Breastfeeding Association in

			2017-2018 under the ACT Health Promotion Grants Program (Link). In 2013-2015 funding focused on overweight and obesity, newer funding models focus on chronic disease and social inclusion.
A.2 Partnerships	A.2.1 Are partnerships across government noted in 'key policy' identified above?		Yes. As noted above, the ACT interview identified that the creation of the action plan was a collaborative effort. Across the six themes of *Towards Zero Growth*, partnerships with multiple agencies named (see next point for agency leads of themes).
	A.2.2 Are there formal mechanisms for collaborative exchange across sectors (e.g. working groups, policy/outcome joint statements, embedded health positions in agencies outside of health)?		*Towards Zero Growth*: The strategies for obesity prevention are noted as happening 'beyond the health sector' and different organisations take the lead across the six work areas (Food environment: ACT Health. Schools: Education and Training Directorate. Workplaces: Chief Minister and Treasury Directorate. Urban planning: Environment and Sustainable Development Directorate. Social inclusion: Community Services Directorate. Evaluation: ACT Health). The ACT informant noted the collaborative processes were "sort of Health in All Policies, but by stealth" (**ACT informant**).
A.3 Equity	A.3.1 Do the key policies identified outline the structural (incl. social/commercial) causes of obesity?<br>(such as employment/family income, affordable or social housing, adverse early childhood experiences, food security, food systems including promotion, built environment and access to safe/appropriate spaces for being active, etc)		Some. Food and built environments do feature as causes of obesity, positioned as: while it is up to the individual to make health choices, governments can influence the environments in which people make decisions about eating and being active. However, while there are some related policies and programs that seek to address housing, food security, adverse experiences, they are not directly linked to preventive health – nor noted in the key obesity prevention policies.<br>Nutrition Australia (an NGO) delivers a range of government programs and services (e.g. assist businesses to comply with ACT Healthy Food and Drink Policy), support Community Services Directorate to help make food banks/ emergency food relief have healthier offerings.<br>*Healthy Canberra Grants* support OzHarvest training for healthy eating knowledge and skills for cooking on a budget for people facing food insecurity and emergency food relief staff (i.e. not targeting structural causes of food insecurity and the focus is on emergency food relief rather than chronic food insecurity).<br>*The Canberra Plan: Towards our Second Century* (Link) noted future priorities of early intervention for at risk families, support for children in public/social housing, early childhood centres.<br>*ACT Food and Nutrition Strategic Framework 2012-2018* (Link) note the Health Directorate can support other sectors to address the socioeconomic determinants of nutrition/food insecurity.
	- A.3.1a Do recommendations for action/initiatives address these structural causes?		In *Towards Zero Growth* recommendations to address environmental causes are focused on closed (e.g. schools) and open (e.g. advertising in public places) settings, e.g. workplaces, planning for active travel, schools as key settings for healthy environments, engagement with food retail.

			However, the 'Social inclusion' theme focuses on personal responsibility approaches to experiences of food insecurity (i.e. awareness of nutrition, cooking skills and confidence).
	A.3.2 Are target populations (with higher risk of developing obesity) identified for additional support?		Yes, both in direct programmatic support and in terms of identifying support from the food sector. Target populations identified as low socioeconomic households and parents from culturally and linguistically diverse backgrounds.<br>A range of health services are available to support Aboriginal and Torres Strait Islander families (Link).
**B. Environments in which we live (e.g. work, shop, eat, be active and play)**		**ACT**	
B.1 Health supportive environments	B.1.1 Do planning policies orientate built environments towards principles of active living?		*ACT Planning Strategy* Link (and website for planning strategy Link). The *Territory Plan* (Link) guides development and planning for the ACT (statutory document, supported by the Planning and Development Act 2007). ACT Health was involved in the review of the planning guidelines and the Planning Act. The Planning Act now requires that developers address 'active living principles' in their applications.<br>The ACT informant noted engagement with the education sector on physical activity/active living aligned with the national curriculum, and the use of evidence aimed at meeting education goals was key to partnering in this space: "We actually have a study in the ACT to demonstrate that… [children that] are more physically active and engaged in things, actually do better in their NAPLAN [education standardised testing] scores. That I think has assisted, you know, 'If your key KPI is for good NAPLAN scores ... well we can help you with that'" (**ACT informant**).
	B.1.2 Are there investments for public infrastructure (e.g. footpaths or bikeways) to encourage being active?		*Transport for Canberra: Transport for a sustainable city 2012-2031* (Link) – public transport and active transport modalities are promoted in an aspirational sense as the desired primary form of travel within the territory. Built around the case for designing the city as a compact, walkable space (a city 'designed to reduce travel'). Allocation of $30 million to active travel in 2016 (over 4 years). Expenditure on path maintenance is presented collectively with road maintenance expenditure.<br>ACT has advantage in active transport policy space as the ACT government is both territory and local government so direct investment into paths is more straightforward.<br>The *Healthy Weight Initiative Progress Report* (June 2016, Link) notes that *ACT Active Travel Framework* and the Active Travel Office to link up transport modes.
	B.1.3 Are there food/nutrition policies aimed at ensuring a nutritious, affordable, accessible food system? (e.g. incentivise local food production or increase healthy food access in disadvantaged communities, zoning policies, or incentives to retailers)		There were no specific food/nutrition policies. These are mentioned under the social inclusion theme of *Towards Zero Growth*. "Implementation may include joint initiatives with local food markets, retailers or community groups who provide existing support services to low-income groups" (p.18). Mapping did not find any explicit examples of programs in this space and wording implies emergency food relief rather than structural programs to increase local food access and affordability. Although the ACT had many initiatives in the food space overall, an overarching policy framework was missing. This may due to the approach taken to build upon

			existing successes and the difficulties in making blanket claims about efficacy across the food system. It becomes difficult to make "some of the economic arguments around [nutrition] because attribution is so challenging" (**ACT informant**).
	B.1.4 Are there programs to support vendors to improve food offerings in food outlets (restaurants, cafes, take-away, vending machines)?		*Healthy Choices Canberra* (Link) is an initiative that has partnered with the Canberra Business Chamber, Nutrition Australia ACT (funded to provide support to food outlet and retail), and public food vendors such as cafes and restaurants, as well as sporting venues and kids venues, to improve the food offerings to families. It also has an arm that looks at improving food retail. Healthier options are indicted with 'HC' icons. Participating venues are referred to as 'local heroes' and are listed on a website (Link).
	B.1.5 Is nutrition information at food outlets (menu board labelling) required by legislation?		Yes. In 2017 the ACT implemented menu board labelling regulation, based on the NSW legislation, except in the ACT the regulation applies once a business has seven stores in the ACT (whereas in NSW it applies once there are 20 stores). The ACT policy aligns with the NSW policy in terms of applying to companies with 50 stores nationally (*Kilojoule displays* Link).
	B.1.6 Is there engagement with food retail (supermarkets, grocers, corner stores, etc) to reduce the availability and promotion of discretionary choices in-store?		*Healthier Choices Canberra* does extend to some food retailers in Canberra (see B.1.4) and are also listed on the website as 'local heroes'. The majority of food retailers participating here are 'IGA' and 'Friendly Grocer' affiliates (i.e. not part of the two major supermarket chains who constitute ~80% of the national food retail share, Coles and Woolworths). The main public facing feature of this program is to display tags on shelves to identify healthier comparable choices. It also lists some suppliers appropriate for catering/functions.<br>While *Towards Zero Growth* identified partnerships with food retail (i.e. mandate that checkouts have at least one aisle without discretionary choices), the *Healthy Weight Initiative Progress Report* (June 2016) omitted that action area.
	B.1.7 Are local governments empowered to encourage health-supportive environments?		ACT is a territory with semi-autonomous authority and acts across policy areas devolved to both 'state' and local government.
	B.1.8 Are there any initiatives to reduce exposure to the **marketing/promotion** of discretionary choices in:<br>- B.1.8a out-of-home advertising (billboards, transport vehicles, street furniture, transport hubs such as train stations) within government control?		Transport Canberra removed junk food advertising from ACTION buses in 2015 (Link), and then light rail later. This was driven by an announcement from the Transport Minister, and support was provided by ACT Health Directorate to define the criteria for the guidelines. Like the school canteen menu changes, there were concerns that this policy may lead to a loss of revenue for the department, but later economic analysis revealed no net loss of revenue for this policy. Transport has taken an active role in linking key policy space elements over time including sustainability and climate change mitigation, health and physical activity, planning a walkable city, ensuring access to public transport in addition to the traditional elements of transport (infrastructure, safety, congestion).<br>This policy does not extend to out-of-home advertising within the ACT government control.

	- B.1.8b healthcare settings?		*ACT Healthy Food and Drink Choices Policy* (Link) seeks into increase the range of healthy foods and drinks available and promoted to staff and visitors to health facilities, as well as ACT Health functions and events. Based on a traffic light system (developed by Nutrition Australia, an NGO), goal is to have majority 'green' foods and a maximum of 20% 'red' foods and drinks.
	- B.1.8c other government-controlled buildings/parks?		ACT Public Sector (ACTPS) policies apply to all government facilities and to vendors selling at these settings and includes 'advertising, promotion and placement of foods and drinks at ACTPS workplaces, facilities, activities and functions'. *ACTPS Healthy Food and Drinks Choices Policy* (Link) and *ACTPS Vending Machine Management Policy* (Link). These policies use the same traffic light system and food marketing/promotion is monitored in these settings.
	B.1.9 Are there policies limiting the **availability/provision** of discretionary choices in:<br>- B.1.9a healthcare settings (for visitors and staff)?		*ACT Healthy Food and Drink Choices Policy* – noted under marketing/promotion above. This policy supports increases in healthier food options with limits on energy dense, nutrient poor. It expanded from ACT health and education settings to across government. "So it started out with an ACT Health policy and then that was adapted into a ... all of government thing and so, for example, vending machines… they have been removed from schools. There are none… [And] all vending machines that are in government workplaces and things like that have to meet quite strict criteria" (**ACT informant**).
	- B.1.9b buildings, community centres, and parks under government control?		*ACTPS Health Food and Drink Choices Policy* and *ACTPS Vending Machine Management Policy* seek to increase the availability of healthy alternatives in food service outlets at ACT Government workplaces and facilities. This requires ongoing engagement with and support for small business operators on-site. This establishes a workforce to support businesses in the broader community seeking to develop healthier food and drink offerings.<br>The *ACT Nutrition Support Service* Link received initial grant under the Healthy Weight Initiative in 2014-2017 to support ACT Government settings and select community settings and organisations. The service is delivered by Nutrition Australia ACT (an NGO) and supported settings to create healthy eating environments.
B.2 Health promotion campaigns	B.2.1 Are there health promotion campaigns aimed at:<br>- B.2.1a encouraging healthy lifestyle behaviours?		*Good Habits for Life* (0-8y) promoted healthy lifestyles for families, it linked up with the *Healthier Choices* (Link) initiative there is *The Great Canberra Cook Off* (Link), with a category for 8-12 year olds (Little Chef)
	- B.2.1b developing/supporting healthy food systems and built environments (incl. community-capacity building)?		*It's Your Move* was a public engagement campaign, not an obesity prevention campaign but it did engage high school students to use design thinking to come up with innovative solutions to self-identified issues within their schools. The initiative has won international awards and many of the projects focused on food and physical activity environments (within school settings).
**C. Early childhood education and care (ECEC) settings**		**ACT**	
C.1 ECEC settings	C.1.1 Are there support programs for centre-based care settings to:		Healthy food policies in school settings exist in most Australian jurisdictions, although such requirements have not been extended to the ECEC sector. The ECEC

	- C.1.1a encourage healthy food provision? (e.g. management: policies and menu audits; staff: training and resources; families: resources)		sector is regulated nationally under the National Quality Framework (NQF) (those regulations are implemented and monitored at a state/territory level through either the education or communities departments), so it would make sense to develop these standards in a nationally consistent way. The *feedAustralia* initiative offers support to ECEC sector via an online menu planning tool menu reviews (Link) – those jurisdictions which do not already provide such services could encourage services to access this program.<br>The *ACT Nutrition Support Service* (Link) received an initial grant under the Healthy Weight Initiative in 2014-2017 to support settings, including ECEC settings (ACT Government and select community settings and organisations). The service was delivered by Nutrition Australia (an NGO) and supported settings to create healthy eating environments through services such as professional development training. At the time of mapping, this service was no longer available to ECEC services although the website was still being maintained, including access to the simple resource *Menu Planning in Childcare* (Link). Other support services are available to ECEC settings under a fee-for-service model.<br>*Fresh Taste* (Link) free program for schools (starting with public schools) targeted food environment and culture around food at schools. Menu items were grouped based on traffic light criteria; schools are supported to develop school-wide food policies; additional support is provided to canteens. It coincided with the removal of vending machines from schools and worked with school parents' organisations to attenuate fears surrounding potential loss of revenue for school canteens. This was also supported by the ACT Nutrition Support Service (Nutrition Australia ACT). Ongoing support for food provision (agreements with canteen operators) and food and nutrition curricula (resources).
	- C.1.1b provide food and physical activity experiences as part of the curriculum?		Program *Kids at Play Active Play* (Link) free program. Promotes fundamental movement skills, active play and discourages screen time; embedded physical activity skills into early childhood university courses and worked with the Teacher Quality Institute to offer accredited professional development training. The interview participant noted that, "we find that that gets teachers engaged, if you can give them professional development points, and it's something they're interested in then they participate in the things" (**ACT informant**).
**D. Health services**		**ACT**	
D.1 Antenatal and birth services	D.1.1 Does antenatal care screen and manage hypertension, hyperglycaemia, appropriate gestational weight gain?		*Clinical Practice Guidelines: Pregnancy Care 2019 edition* (national guidelines) recommend monitoring of blood pressure, weight and screening for hyperglycaemia (Link)
	D.1.2 Antenatal care within public health services:		
	- D.1.2a Do they include nutrition counselling for healthy pregnancy or are there other healthy lifestyle support programs available during pregnancy?		No services found at the time of mapping, although there is a booklet called *Good Nutrition in Pregnancy* (Link).

	- D.1.2b Is breastfeeding education free (separately or embedded into antenatal education/services)?		Not clear. Although the *Early Pregnancy and Parenting Support* line (D.2.1a) does list breastfeeding support, as a phone line it is unlikely to offer the hands on support needed to really develop breastfeeding skills. Antenatal breastfeeding education classes are run for a fee by the Australian Breastfeeding Association.
	D.1.3 Do maternity facilities fully adhere to the Baby Friendly Health Initiative (BFHI) (based on *Ten Steps to Successful Breastfeeding*)?		Two hospitals have received BFHI accreditation, although Canberra has a range of birthing centres also (Link) – no additional policy requiring public health facilities to become BFHI accredited.
D.2 Early childhood services	D.2.1 Are there free health/parenting services to support early childhood growth/nutrition (e.g. breastfeeding, complementary feeding, transition to family foods)?		Maternal and Child Health (MACH) services provide a range of support for parents during early childhood, support by MACH nurses. These include first home visits, drop-in clinics as well as scheduled appointments, new parents groups (four sessions for infants under 4 months), and additional sessions on breastfeeding and ‘understanding your baby’. Families are provided with a ‘Blue Book’ for their child’s personal health record and include regular check-ups at MACH check-ups (at 11 Child Health Clinics in ACT, 3 are collocated with Child & Family Centres), There are also three Child & Family Centres (Link) across the ACT that provide support and programs for parents of children aged 0-8 years.<br>Additional pamphlets include Tucka talk: baby’s first foods (Link), food for 1-3 year old’s (Link); food for your 4-6 year old (Link).<br>Community dietitians are available for support for a range of pregnancy, breastfeeding and young child feeding (Link)
	- D.2.1a Is information to support parents readily available (e.g. phonelines, websites)?		*Early Pregnancy and Parenting Support* line (Link) provides support during early pregnancy, breastfeeding, and maternal emotional wellbeing. It could be expanded to provide additional support around nutrition and early movement.<br>*Early Parenting Counselling* (Link) is a free service to support parents of 0-5year old’s – this service does not support healthy lifestyle behaviours, but it does support attachment and mental health.
	- D.2.1b Do these include breastfeeding support?		The Early Pregnancy and Parenting Support line (D.2.1a) includes breastfeeding support in addition to drop in clinics via MACH services (see D.2.1), booklet to support maternal nutrition during breastfeeding, *Good nutrition while breastfeeding* (Link).
	D.2.2 Are there healthy lifestyle (education) programs to support families during early childhood?		None found at the time of mapping
	- D.2.2a Are target populations identified and actively recruited for programs?		n/a
	D.2.3 Are Supported Playgroups offered for families that need additional support and do they include healthy lifestyle skills?		None found at the time of mapping
D.3 Workforce	D.3.1 Are there training and resources available for health care professionals to support families?		None found at the time of mapping
	- D.3.1a Is preconception advice for nutrition and being active provided to prospective parents?		None found at the time of mapping

	D.3.2 Is there a state/territory health promotion…<br>- D.3.2a …agency (independent or adjunct to health department)?		No – health promotion work is embedded within health department activities (no separate agency). Because ACT government acts as territory and local government, local implementation of programs is part of core business (often in partnership with NGOs, e.g. Nutrition Australia ACT). Population monitoring and surveillance is undertaken via annual computer-assisted telephone interview (CATI) self-reported anthropometry, health status, health behaviours – target 1200 adults and 500 children (2-15 years) (*ACT General Health Survey* Link)
	- D.3.2b …workforce (to implement initiatives locally)?		There is a health promotion workforce embedded within the ACT Health directorate

2.2 New South Wales

Area	Guiding questions	Result	Notes
**A. Governance & leadership**		**NSW**	
A.1 Leadership	A.1.1 Has childhood obesity prevention been identified as a priority by leadership (Premier/First Minister or Health Minister)?		Leadership is a priority: there is the *Premier's Priority* to reduce childhood obesity by 5% by 2025. It is also a health department priority through the *Healthy Children Initiative (HCI).*
	A.1.2 **Key policy**: Is there an overarching policy framework, or a series of key policies or action plans to guide initiatives for the early prevention of obesity in childhood?		The *Healthy Children Initiative* (Link) commenced in 2011, funded by the NPAPH and continued despite national funding cuts. HCI is funded by the Ministry of Health (MoH) and delivered by the Office of Preventive Health (OPH) and across NSW's 15 Local Health Districts (LHDs). This strategy takes a settings-based approach to deliver state-wide programs to improve healthy lifestyle behaviours.<br>Although the *Premier's Priority* target is for a 5% reduction of obesity in school aged children, it is implicit that in order to achieve the target for that age group in 2025, sustained actions need to be taken in the early years and during pregnancy.<br>The *Healthy Eating Active Living Strategy: Preventing overweight and obesity in New South Wales 2013-2018* (HEAL) (Link) was a whole of government framework to support healthy eating and being active, i.e. a risk factors approach to prevention. The strategy has four directions: built environment (see section B.1), state-wide programs (see section C.1), routine health service delivery (see D.3.1), and public education campaigns (see section B.2). Within the state-wide framework sits the HCI programs, one of which is the *Munch & Move* program – aimed at ECEC settings (see section C.1 for more details). The *Get Healthy Service* (see section D.1.2a) also sits under the *HEAL Strategy* and provides a telephone healthy lifestyle coaching service for adults.<br>The *First 2000 Days Framework* (Link) is a health system strategy to encourage all NSW health professionals to understand the importance of the first 2000 days and provide continuity of care from the antenatal period until children commence school. It builds on the First 1000 Days work, but "NSW Health has chosen to expand the focus beyond the first 1000 days to the first 2000 days of life to incorporate additional evidence that quality early education in the preschool years has a strong bearing on long term outcomes" (p.13). This effectively ties their early years focus to health and ECEC settings.
	A.1.3 Does the state/territory legislation for public health include prevention/health and wellbeing?		The *Public Health Act 2010* & *Public Health Amendment (Review) Act 2017* (Link) were reviewed and there were no references to wellbeing or health promotion that related to prevention of chronic disease.
	A.1.4 Are their statutory grant-giving bodies with a remit to fund prevention-related community projects?		There are no statutory or grant-giving organisations but the MoH funds many programs at the LHD level (both directly and indirectly through health promotion budgets) and grants such as the Translational Research Grants to encourage evidence-based interventions delivered at scale.

A.2 Partnerships	A.2.1 Are partnerships across government noted in 'key policy' identified above?		Yes, the HEAL strategy identifies who are the main and secondary partners with each of the actions listed under the 'What we will do' headings
	A.2.2 Are there formal mechanisms for collaborative exchange across sectors (e.g. working groups, policy/outcome joint statements, embedded health positions in agencies outside of health)?		HEAL Strategy Senior Officers Group (HEAL SOG) holds a quarterly meeting to report on the progress of actions outlined in the strategy. It is attended by senior policy officers across government and co-led by MoH and the Department of Premier and Cabinet (DPC). Additionally, the DPC Premier's Implementation Unit provides structural support to organisations at the implementation level to identify and overcomes barriers.
A.3 Equity	A.3.1 Do the key policies identified outline the structural (incl. social/commercial) causes of obesity? (such as employment/family income, affordable or social housing, adverse early childhood experiences, food security, food systems including promotion, built environment and access to safe/appropriate spaces for being active, etc)		The HEAL strategy does not directly talk about the structural causes of obesity as they relate to family housing and income or broad food systems, nor the commercial influences of obesity. It notes the problem of rising obesity are the personal choices of individuals, and the solutions are to support people to make healthy choices, "and create an environment that supports healthier living through better planning, built environments and transport solutions" (p.7).<br>HEAL does mention investing in research about food security for disadvantaged and remote communities.
	- A.3.1.a Do recommendations for action/initiatives address these structural causes?		HEAL strategy actions are aimed a food and built environments as well as settings, but do not identify other policies to address more structural causes.<br>There are some other policies which relate to housing and early childhood adverse experiences. *Healthy, Safe and Well* (see D.2.1) – identifies the importance of early intervention to support families and minimise adverse early childhood experiences.<br>There are some housing policies, although they tend to be targeted and do not support the principle of ensuring the population is well housed. *Housing for Health* (Link) assesses, repairs, or replaces health hardware in Aboriginal community housing (the program uses a proactive 'survey and fix' methodology) –health hardware for nutrition is one of four 'critical' principles. The program originated in South Australia aimed at remote communities (*Health habitat*, Link) with a specific focus on children aged 0-5 years. Updated map of the projects, by LHD (Link)
	A.3.2 Are target populations (with higher risk of developing obesity) identified for additional support?		In the HEAL strategy priority populations were identified, these were: Aboriginal communities, Culturally and Linguistically Diverse communities, regional and remote communities, and socio-economically disadvantaged communities. It notes that strategies and programs that sit under the HEAL framework are "appropriate for, and responsive to, NSW's diverse communities" (p.21) in addition to "specific and targeted actions for Aboriginal people" (p.20).
**B. Environments in which we live (e.g. work, shop, eat, be active and play)**		**NSW**	
B.1 Health supportive environments	B.1.1 Do planning policies orientate built environments towards principles of active living?		The NSW planning Act was recently updated. While the initial consultation draft that was circulated included community health and wellbeing, it was omitted in the legislation that passed. The *Environmental Planning and Assessment Act 1979 No 203* (Link) includes the principle of 'good design'. This is supported by the *Better Placed* (Link) suite of guidance support for liveability, productivity, and environmental management in design, from the Office of Government Architect. The Better Placed policies do identify the relationship

			between the built environment and health and wellbeing. There are no levers to challenge developers who do not implement principles of 'good design'.<br>The *Healthy Urban Development Checklist* (2009, Link) was developed by NSW Health and Sydney South West Area Health Service (now a LHD) to encourage healthier built environments.
	B.1.2 Are there investments for public infrastructure (e.g. footpaths, bikeways or greenspace) to encourage being active?		NSW does not have a physical activity plan, but active living is a key principle of the HEAL strategy.<br>The *Walking and Cycling Program Guidelines* (Link) for funding a range of projects relating to walking and cycling (infrastructure, non-infrastructure, planning and design, metropolitan and regional). A range of policies and programs relate to increasing active transport (Transport for NSW) (Link). *NSW Active Charter for Children* (Link) is an active transport plan for school aged children.<br>A Sydney-based series of policies around increasing green space in urban settings, such as the *Green Grid* and urban tree canopy to connect pathways, cycling paths, parks and open spaces (Link), sits under the policy *A Metropolis of Three Cities* with the Greater Sydney Commission (an independent organisation funded by the NSW government that has strategic oversight on policy across both state and local government in the greater Sydney area, e.g. planning, environment, etc).
	B.1.3 Are there food/nutrition policies aimed at ensuring a nutritious, affordable, accessible food system? (e.g. incentivise local food production or increase healthy food access in disadvantaged communities, zoning policies, or incentives to retailers)		NSW does not have a nutrition policy, but healthy eating is a key principle of the HEAL strategy. HEAL states that health will partner with the planning and infrastructure department to preserve and where possible increase local food production; partner with councils to encourage healthier cooking practices in local food outlets; support research into food security. It also covers a few select settings for healthy food policies (see B.1.9 and C.1.1)<br>Other policy areas include maintaining existing menu labelling regulations and working nationally to impact on front-of-pack labelling, support food reformulation dialogue, reduce children's exposure to marketing.
	B.1.4 Are there programs to support vendors to improve food offerings in food outlets (restaurants, cafes, take-away, vending machines)?		There were no state-wide programs to engage with food retailers to improve food offerings. However, there were a few instances where local councils sought to support small food outlets to swap to healthier cooking oils. These occurred under the authorising environment of the *HEAL Strategy*, where efforts to make cooking oils healthier were a key action.<br>The Heart Foundation's *Healthier Oils* program partnered with the Food Authority and local environmental officers (dedicated positions in local councils) in the Cessnock Local Council (Link). The Western Sydney Local Health District partnered with the Parramatta Local Government Area were influenced by this program and undertook an environmental scan and a pilot study (Link)<br>These relationships across LHDs health promotion officers, local councils and environmental health officers, and small to medium food outlets provide examples of the types of programs that could potentially be developed to effect different elements of the foods served in these venues, and how they can be undertaken locally.
	B.1.5 Is nutrition information at food outlets (menu board labelling) required by legislation?		In 2010 NSW amended the *Food Act*, requiring all chain food service outlets (with >20 stores in NSW or >50 stores nationally) to display kilojoule values next to each item on the menu -

			kJ information (Link). This legislative change in NSW sparked the progression of most state and territory governments to follow by amending their own food Acts. Work to ensure national consistency was undertaken with the COAG Obesity Working Group.
	B.1.6 Is there engagement with food retail (supermarkets, grocers, corner stores, etc) to reduce the availability and promotion of discretionary choices in-store?		None found at the time of mapping
	B.1.7 Are local governments empowered to encourage health-supportive environments?		There were 128 local governments in NSW at the time of mapping. There were some significant state government efforts to amalgamate councils, to some success.<br>Most of the 15 LHDs Health Promotion Units engage with some/most of the local councils within their district to promote health, however, local governments are limited in what they can achieve without the legal right to enact many policy tools. While many jurisdictions are seeking partnership models with local governments, NSW prefers a decentralised approach.
	B.1.8 Are there any initiatives to reduce exposure to the marketing/promotion of discretionary choices in:<br>- B.1.8a out-of-home advertising (billboards, transport vehicles, street furniture, transport hubs such as train stations) within government control?		None found at the time of mapping
	- B.1.8b healthcare settings?		The *Healthy Choices in Health Facilities* policy (see B.1.9) does not mention reducing promotion or marketing of unhealthy foods in NSW Health facilities. It does mention the promotion and increasing the availability of healthy food and drink options.
	- B.1.8c other government-controlled buildings/parks?		None found at the time of mapping
	B.1.9 Are there policies limiting the availability/provision of discretionary choices in:<br>- B.1.9a healthcare settings (for visitors and staff)?		The *Healthy food and drink in NSW health facilities for staff and visitors: Healthy Choices in Health Facilities* 2017 (Link) applies to all food outlets in NSW Health facilities selling drinks and food to staff and visitors. It uses the *Health Star Rating* (Link) to determine the 'healthy' status of products (all other jurisdictions that have such policies use a traffic light system), i.e. products with 3.5 stars or above indicate 'healthier options' under this framework. This policy also requires the removal of SSBs from all Health facilities.
	- B.1.9b buildings, community centres, and parks under government control?		None found at the time of mapping
B.2 Health promotion campaigns	B.2.1 Are there health promotion campaigns (any media type) aimed at encouraging healthy lifestyle behaviours?		The *Make Healthy Normal* campaign was a health promotion campaign at the time of mapping (Update 2021, that campaign has finished and all links now re-route to the Healthy Eating Active Living website, Link). It encouraged healthy lifestyle behaviours, often using messages aimed at families although nothing specifically about early childhood.
	B.2.2 Are there health promotion campaigns aimed at developing/supporting healthy food systems and built environments (incl. community-capacity building)?		None found at the time of mapping
**C. Early childhood education and care (ECEC) settings**		**NSW**	

C.1 ECEC settings	C.1.1 Are there support programs for centre-based care settings to encourage healthy food provision? (e.g. management: policies and menu audits; staff: training and resources; families: resources)		Ongoing support for the ECEC sector is provided through the *Munch & Move* program (Link). Centre-based care (i.e. long day care) carers, educators, and services receive support and training through the dedicated health promotion workforce embedded within NSW 15 LHDs. The program has high reach with ~87% of centre-based care centres (>3500) at the time of mapping, and looking to extend to different service types, e.g. "we're just about do a validation study on much of these practices in family day care" (**NSW informant**). Also, to use the program to engage with parents "The [ECEC] programs do have an element of keeping parents informed and engaging them in the program, and we monitor that through our program induction indicators or practices" (**NSW informant**).<br>*Munch & Move* has centralised support through the Office of Preventive Health and digital infrastructure (e.g. website, eLearning program training), in additional to the local health promotion workforce embedded into each LHD. The program supports centres to provide healthy foods and beverages (or ensure healthy lunchboxes) and opportunities to be active and limit screen time. The nutrition elements of the program are supported by the *Caring for children: Birth to 5 years* guidelines (Link). The program has used the PHIMS reporting mechanism to justify ongoing investment (see D.3.2b for more information about PHIMS). The Commonwealth-funded (but NSW developed) *feedAustralia* initiative offers support to ECEC sector via an online menu planning tool and menu reviews (Link).
	C.1.2 Are there programs to support provision of food and physical activity experiences as part of the curriculum?		*Munch & Move* provides support for curriculum development in physical activity and nutrition at the service level and provides in-training to the appropriate staff within the Early Childhood Directorate within the Department of Education.
**D. Health (community and tertiary health settings and health promotion activities)**		**NSW**	
D.1 Antenatal and birth services	D.1.1 Does antenatal care screen and manage hypertension, hyperglycaemia, appropriate gestational weight gain?		*Clinical Practice Guidelines: Pregnancy Care 2019 edition* (2018 national guidelines) recommend monitoring of blood pressure, weight and screening for hyperglycaemia (Link). The *Standard Schedule of visit for low risk women* (e.g. from Western Sydney LHD, Link) sets out how and when screening for these potential issues occurs during usual antenatal care.
	D.1.2 Antenatal care within public health services:		Antenatal care within the public health system is offered in antenatal clinics at public hospitals or midwives' clinics at either a birth centre or a Community Health Centre. The *Having a Baby* book (2012) (Link) covers a range of information to support women through the process of pregnancy and having a baby in the public health system in NSW. Antenatal classes may incur a fee in some areas where they are not offered for free in the public system, they are not offered as state-wide routine care.
	- D.1.2a Do they include nutrition counselling for healthy pregnancy or are there other healthy lifestyle support programs available during pregnancy?		Referrals to dietitians and physiotherapists are available through antenatal clinics, if needed. The Having a Baby book recommends daily exercise in addition to specific pre- and postnatal exercises (after checking with doctor to make sure there are no health problems), provides healthy eating information and discusses healthy weight gain during pregnancy. Antenatal classes may offer healthy lifestyle information for pregnancy, but classes are not universally available as part of routine state-wide care. However, more broadly, NSW offers *Get Healthy in Pregnancy* (Link) a module within the *Get Healthy Information and Coaching Service* (Link), a telephone-based healthy lifestyle coaching service offered at a time convenient for

			the participant. The service is funded by the Office of Preventive Health and delivered by a third party (Healthdirect/ Remedy Healthcare). Coaches are required to have appropriate health qualifications and undergo specific training.
	- D.1.2b Is breastfeeding education free (separately or embedded into antenatal education/services)?		Breastfeeding education may be offered in public but antenatal classes are not available as part of routine state-wide care. The *Having a Baby* book has a section on breastfeeding and introduction to solids, and there is an additional booklet, Breastfeeding your baby (Link)
	D.1.3 Do maternity facilities fully adhere to the Baby Friendly Health Initiative (based on *Ten Steps to Successful Breastfeeding*)?		The BFHI is a strategy noted under the *Healthy, Safe and Well* strategic plan.<br>The Policy Directive *Breastfeeding in NSW – Promotion, Protection and Support* (Link) includes system wide recommendations to promote, protect and support breastfeeding across the NSW Health system (in addition to private health settings and environmental health officers of local councils). The role of this Policy Directive is to provide frameworks for the BFHI in maternity services, community facilities and neonatal services.<br>At the time of mapping 10 hospitals and Child & Family Health Services were BFHI-accredited. More services likely utilise relevant BFHI frameworks but have not achieved full accreditation.
D.2 Early childhood health services	D.2.1 Are there free health/parenting services to support early childhood growth/nutrition (e.g. breastfeeding, complementary feeding, transition to family foods)?		*Healthy, Safe and Well; A strategic health plan for children, young people and families 2014-2024* (Link) (NSW Kids and Families), Strategic direction 1 focuses on the care for women and babies and provide transition support from postnatal care to parenthood such as connecting families to services.<br>*Child and Family Health Centres* (Link) are available across the state. They provide health, development, and wellbeing checks at set time points (1-4 weeks, 6-8 weeks, 6 months, 12 months, 18 months, 2 years, 3 years, 4 years). Additionally, there are 'drop-in' clinics and other education/ information opportunities – guided by the Policy Directive *Maternal & Child Health Primary Health Care Policy* (Link). *Sustained home nursing visiting services* are available for families who need additional support in five sites across the state (Link)
	- D.2.1a Is information to support parents readily available (e.g. phonelines, websites)?		The *Healthy kids* website has little information for children in the early years (Update 2021, Healthy kids website no longer supported by NSW government, new website with early years content called *Healthy Eating Active Living* (Link) which is different to the HEAL strategy)
	- D.2.1b Do these include breastfeeding support?		Breastfeeding information/support is available at the Family and Child Health services (e.g. many LHDs offer drop-in centres to support feeding) but no support is offered over the phone as it is in other jurisdictions.
	D.2.2 Are there healthy lifestyle (education) programs to support families during early childhood?		The *Healthy Beginnings* program (Link) originated as a research trial delivered with vulnerable families through some existing sustained home visiting services. The program included support with feeding and movement during key life stages. The program was then trialled as a counselling service over the telephone or via SMS for low risk families, called the *Communicating Healthy Beginnings Advice by Telephone* (CHAT) Trial (see Link). At the time of data collection NSW had announced they would embed the *Healthy Beginnings* program (as the CHAT Trial) into the *Get Healthy Service* (Link) as an telephone support service for parents of young children (0-2 years). "What we don't have in programs currently is direct contact with parents… So with the Get Healthy Service as a platform, for Healthy Beginnings that is likely to be developed into a service for parents of zero to two year olds"

			(**NSW informant**). There was also a Translational Research Grant to test the feasibility of a similar program for parents of children aged 2-6 years. In addition, the Healthy Beginnings program would further be scaled up into all sustained home visiting services across the state.
	- D.2.2a Are target populations identified and actively recruited for programs?		Healthy Beginnings was initially designed to support vulnerable families through existing home visiting services, which was intended to be scaled to all home-visiting services
	D.2.3 Are Supported Playgroups offered for families that need additional support and do they include healthy lifestyle skills?		Supported playgroups have been provided with resources but not any ongoing support, "We don't have a program in Supported Playgroups. What we've done is develop a range of resources. The LHDs have had a brief period where they've made contact with any Supported Playgroups that they could find in their area and encouraged them to use resources. Our plan in that was to work with auspice organizations but we don't have resourcing to do that right now" (**NSW informant**)
D.3 Workforce	D.3.1 Are there training and resources available for health care professionals to support families?		The *Healthy kids for professionals* website (Link) is designed to support health professionals to follow the national weight management guidelines along the asses, advise, assist, arrange support and referral pathway. It provides resources to support sensitive discussions with families about child weight and how to refer children/families into programs (if available) or onto specialised services. This aligns with strategic direction 3 of the *HEAL Strategy* (healthy eating and active living advice as part of routine service delivery).<br>The Health Education and Training Institute (HETI) offers a range of training modules for NSW Health staff, e.g. My Health Learning Course Code: *45338916 Breastfeeding Promotion, Protection and Support* (Link). Records of courses completed follow NSW Health employees across a range of settings to retain accreditation across workplaces and ensures mandatory training is kept up to date.
	- D.3.1a Is preconception advice for nutrition and being active provided to prospective parents?		There is a *Thinking of Having a Baby* brochure (Link) outlining preparation 3-6 months before pregnancy, but the information provided is limited (and does not mention being active)
	D.3.2 Is there a state/territory health promotion…<br>- D.3.2a …agency (independent or adjunct to health department)?		The OPH drives state-wide programs via embedded workforce in LHDs (LHDs and the OPH make up the implementation arms of the Ministry of Health). These programs undertake a settings-based approach to their programs, for the early years this situates around the ECEC sector.<br>*Population monitoring*: CATI survey for self-reported anthropometry, health status, health behaviours and access to healthcare. Continuous data collection – all ages (parents are proxies for children under 16 years) (*NSW Population Health Survey* Link).<br>The *Population Health Intervention Management System* (PHIMS) (Link) allows for real time reporting of health promotion activities across NSW in multiple programs under the Healthy Children Initiative, including *Munch & Move*. It records services engaged in the program and the extent of program practices being achieved, at the LHD-level.
	- D.3.2b …workforce (to implement initiatives locally)?		NSW has a dedicated preventive health workforce employed across NSW 15 LHDs in Health Promotion Units, who have relationships with (for early childhood) ECEC centres and staff, and "all the local health districts will have a relationship with their local council, and may be on community development groups and so on" (**NSW informant**).

2.3 Northern Territory

Area	Guiding questions	Result	Notes
**A. Governance & leadership**		**NT**	
A.1 Leadership	A.1.1 Has childhood obesity prevention been identified as a priority by leadership (Premier/First Minister or Health Minister)?		Chronic disease prevention for adults, including obesity and other chronic diseases, is a priority for the NT. The *NT Chronic Conditions Prevention & Management Strategy 2010-2020* (Link) supports a life course approach and highlights the importance of the early years.<br>Between the *Annual Report 2017-2018* (Link) (mid-2018) to *Annual Report 2018-2019* (Link) (mid-2019) NT Health priorities shifted from an acute-focused illness prevention to a population focused illness prevention outlook. While in mid-2018 preventing illness priorities focused on acute services such as *Maternal Early Childhood Sustained Nurse Home Visiting Program* (MECSH) and minimising substance misuse (p.38), by mid-2019 the priorities shifted to prevention of chronic conditions and diabetes, food security, and early childhood (*MECSH* co-designed for use in Aboriginal communities, *Healthy Under 5 Kids*, and align with childhood obesity efforts in the National Obesity Strategy once it is released) (p.39-40)<br>- And MESCH was adapted with ACCHO partners to meet needs of families in community
	A.1.2 Key policy/policies: Is there an overarching policy framework, or a series of key policies or action plans to guide initiatives for the early prevention of obesity in childhood?		Several policies identify pre-conception, pregnancy, and the early years as key life stages to improve health and prevent chronic disease.<br>*The Best Opportunities in Life: Northern Territory Child and Adolescent Health and Wellbeing Strategic Plan 2018-2028* (Link) is a strategic NT Health (and partnerships) plan. It aims to improve health and wellbeing from 0-24 years through an overarching framework to guide health, housing, education, youth justice, child protection and police services. It is aimed at service providers (government, non-government and Aboriginal Community Controlled Health Services). Of relevance is *Priority Action 1.1: All children (and their families) are supported from birth to age five to ensure healthy development and school readiness* which highlights a range of key early childhood initiatives:<br>• Healthy Kids Under 5 (HU5K) (well child programs)<br>• Australian Nurse Family Partnership Program (ANFPP) + Maternal Early Childhood Sustained Home-visiting (MECSH) program<br>• Engagement with ECEC sector (partnership between Dept Education & Health)<br>• Families as First Teachers (FaFT)<br>• Child and Family Centres (expanding from 6 to 17) (Dept Territory Families)<br>• Central Australian Aboriginal Congress: integrated service model for under-fives (Aboriginal Community Controlled Health Services; Dept Health)<br>Priority Action 3.2: Chronic conditions are addressed by health promotion, prevention, and early intervention (p.44) is linked to the *Chronic Conditions Prevention and Management Strategy 2010-2020: Population health and wellbeing* (Link). That strategy's principles include

			a focus on the early years, addressing the social determinants of health, and working in partnership across sectors. The most recent *Implementation Plan 2017-2020* (Link) links into the cross-government approaches to address the social determinants of health in *The Best Opportunities in Life* and the Early childhood Development Plan (*Starting Early for a Better Future*). *Starting Early for a Better Future* (Link) is a 10 year strategy to integrate childhood development services and overcome barriers to collaboration between health, early education and family support. The current *Implementation Plan 2018-2022* (Link) at the time of mapping includes actions and targets for housing reforms, homelessness, access to affordable, nutrition and fresh food for all families.<br>The *NT Health Nutrition and Physical Activity Strategy 2015-2020* (Link) has five objectives including to improve remote food security, healthy gestational weight, early years focus on nutrition and being active, schools, healthy weight for the population and takes a life course approach. It is supported by the Nutrition and Physical Activity Unit within NT Health.
	A.1.3 Does the territory legislation for public health include prevention/health and wellbeing?		Not at the time of mapping
	A.1.4 Are their statutory grant-giving bodies with a remit to fund prevention-related community projects?		Not at the time of mapping
A.2 Partnerships	A.2.1 Are partnerships across government noted in 'key policy' identified above?		Partnerships identified primarily focus on health, education and social services for family support and less so on health supportive environments. *Starting Early for a Better Future* notes all the ways a collaborative approach could be undertaken, but informants noted the capacity to carry out these elements is quite limited. "The *Starting Early for A Better Future* strategy ... [is] where that inter-sectoral stuff is happening. It's pretty clear what the actions are, the expectations and targets, who is responsible..." (**NT informant 1**).<br>"…we don't have that capacity… As far as an overarching policy to reach into different departments to actually to be committed to look at some health changes, but its a good idea." (**NT informant 2**).
	A.2.2 Are there formal mechanisms for collaborative exchange across sectors (e.g. working groups, policy/outcome joint statements, embedded health positions in agencies outside of health)?		None found at the time of mapping
A.3 Equity	A.3.1 Do the key policies identified outline the structural (incl. social/commercial) causes of obesity?<br>(such as employment/family income, affordable or social housing, adverse early childhood experiences, food security, food systems including promotion, built environment and access to safe/appropriate spaces for being active, etc)		The Northern Territory is a sparsely populated, but geographically large jurisdiction. It's capital, Darwin, is classified as an Outer Regional Area by the Australian Bureau of Statistics (ABS) and 60% of the population reside here, a further 20% reside in 'regional centres' (classified as remote by the ABS) Alice Springs, Gove/Nhulunbuy, Katherine, Tennant Creek, the remaining 20% of the population reside in very remote communities. There are more than 70 remote Aboriginal communities (populations estimated between 200—3000 people).<br>The link between housing, early adverse childhood experiences, and food security with health is made in all the policies in A.1.2.<br>Theme 4 in *The Best Opportunities in Life* is Health equity for Aboriginal children and young people is increasing

			Food security features in the *NT Health Nutrition and Physical Activity Plan*. (Update 2021, the CAHS public health nutritionists undertook a needs assessment in Alice Springs for food security in 2019-2020 (Link))<br>*Healthy Under 5 Kids* (HU5K) (Annual Report 2018 (Link)) program is a well-child health schedule for remote Indigenous children offered by the Department of Health and Families (DHF).<br>"The social determinants of health are the key drivers of chronic conditions and include early life circumstances, education, employment, occupation, income, social inclusion, nutrition and substance use. To fully achieve the goals of the Strategy a collaborative, whole of government approach supported by the non-government, private and industry sectors is required" (*Chronic Conditions Prevention and Management*, p.6)
	- A.3.1.a Do recommendations for action/initiatives address these structural causes?		Generally, when there is a Commonwealth-funding partnership, e.g. housing, there tends to be more investment in addressing structural causes.<br>Two initiatives of note, run by the Department of Local Government, Housing and Community Development. The *Our Communities, Our Future, Our Homes (Remote Housing)* (Link) program is an investment of $1.1 billion in 73 remote communities (essentially all remote communities) for new housing, home extensions, maintenance and government employee housing between 2017-2027 (supplemented with $550 million from the Australian Government 2018-2023).<br>The *Homelessness Strategy 2018-2023* (Link) is consistent with the social landlord approach to improve public housing policy and increase supply of affordable and social housing: head leasing; support growth for the community housing sector.<br>The *Remote Indigenous Stores and Takeaways* program has Commonwealth investment and is aimed at increasing access to healthy foods in remote communities (see B.1.6).
	A.3.2 Are target populations (with higher risk of developing obesity) identified for additional support?		Target populations identified in the *NT Chronic Conditions Prevention Management Strategy* include Aboriginal people, people experiencing low socio-economic status, people living in rural and remote areas, and prison inmates.
**B. Environments in which we live (e.g. work, shop, eat, be active and play)**		**NT**	
B.1 Health supportive environments	B.1.1 Do planning policies orientate built environments towards principles of active living?		Several policy documents seek to improve 'liveability' in the NT.<br>*Our Economic Future: Northern Territory Economic Development* (Link) (Department of Trade, Business and Innovation) identify six sectors likely to offer the highest economic growth (several types of mining, agriculture, tourism, and international education). In order to achieve economic growth on those sectors, the population needs to grow to supply a workforce.<br>The Department of Infrastructure, Planning and Logistics supports this policy through several policies. The infrastructure strategy, *Planning for a vibrant future* (Link) supports *Our Economic Future* and together they make up the Northern Territory Government's Economic Development Framework and Infrastructure Strategy. The *10 Year Infrastructure Plan 2018-2027* (Link) is a companion to this strategy and highlights the desire to increase the population and make infrastructure investments to increase the desirability of the NT as a place to live (p.8).

			Principles in an earlier planning scheme suggest that the principles of urban liveability have influenced NT planning policy for some time. See the *Northern Territory Planning Scheme* 2007 (Link) which aims to "promote a more compact urban form in appropriate locations to maximise infrastructure utilisation and enhance urban liveability" (NTPS part 2, section 4.1).
	B.1.2 Are there investments for public infrastructure (e.g. footpaths, bikeways or greenspace) to encourage being active?		The *NT Health Nutrition and Physical Activity Strategy 2015-2020* (Link) objective 3: includes the promotion of active lifestyle for children aged 0-5 years.<br>The *Darwin Regional Transport Plan* (Link) (Department of Infrastructure, Planning and Logistics) integrates with the planning and land use plans. It notes low utilisation with public transport (buses and ferries) reportedly due to low and unreliable services. The policy regards active transport also, goal is to provide connected cycle and walking paths.<br>Darwin has the highest proportion of the population walking and cycling to work, compared to other jurisdictions (Link).
	B.1.3 Are there food/nutrition policies aimed at ensuring a nutritious, affordable, accessible food system? (e.g. incentivise local food production or increase healthy food access in disadvantaged communities, zoning policies, or incentives to retailers)		The *NT Health Nutrition and Physical Activity Strategy 2015-2020* was developed under the NT Health Nutrition and Physical Activity Unit. The Primary Health Care teams in the CAHS and TEHS are responsible for implementing and evaluating interventions under this strategy. Objective 1: improve food security, particularly in remote communities; Objective 3: optimise feeding practices for children aged 0-5 years. This policy notes that children in the NT can be at risk of either under nutrition (Aboriginal children living in remote areas) and that overweight and obesity is an emerging risk among Aboriginal and non-Aboriginal children living in urban communities. The recommended actions for objective 3 include programs at the family and community level (incl. ECEC settings and broader food environments). Under objective 1, the CAHS and TEHS were to undertake needs assessments for food and nutrition security. (Update 2021 CAHS completed the Alice Springs Food Security Needs Assessment 2019-2020 (Link)).<br>Food prices in the NT are some of the most expensive in Australia – even in the capital city of Darwin. People living in remote areas have lower access to healthy foods.<br>Some small inroads have been made further upstream, at the food production end of the food system, although with quite a limited outcome. Under the School Nutrition Program, NT schools were having difficulty sourcing ham with a low enough sodium content to meet the standards. NT Health work with local manufacturers to develop a ham that met the standards. A study participant noted that the "manufacturers did pretty good, but it's actually taken a long time… it's quite a bit of work" (**NT informant 1**).
	B.1.4 Are there programs to support vendors to improve food offerings in food outlets (restaurants, cafes, take-away, vending machines)?		None found at the time of mapping
	B.1.5 Is nutrition information at food outlets (menu board labelling) required by legislation?		Not at the time of mapping. Additional laws in NT were not deemed necessary as large outlets decided to roll out consistent menu labelling in all jurisdictions
	B.1.6 Is there engagement with food retail (supermarkets, grocers, corner stores, etc) to reduce the availability and promotion of discretionary choices in-store?		There were no urban programs found at the time of mapping.<br>Food insecurity in remote areas is noted in the *NT Health Nutrition and Physical Activity Strategy 2015-2020*, where residents have few options in where they purchase their food.<br>The *Remote Indigenous Stores and Takeaways (RIST) program* (see newsletter (Link) and resources (Link)) is aimed at improving the nutritional profile and food affordability in remote

			stores. *Outback Stores* are 23 Commonwealth-government owned stores and have a Healthy Food Strategy. In addition, *Community Stores Licensing* requires stocking healthy groceries to improve access to food. While prices are still higher than urban supermarkets, the mark up on healthy foods is usually lower). An informant noted the difficulty in ensuring age-appropriate long life foods for infants in these stores, "Particularly for the infants, we've had a lot of trouble getting iron rich and suitable foods, potentially texture appropriate as well. We get pouches, I guess for food safety reasons and for convenience, etcetera. They'll often stock them it in stores, and it is very difficult to find high iron, healthy, with vegetables etcetera, products for infants starting solids" (**NT informant 1**).
	B.1.7 Are local governments empowered to encourage health-supportive environments?		None found at the time of mapping.<br>NT has 16 Local Government Authorities (plus 5 unincorporated areas, representing 4% of population)
	B.1.8 Are there any initiatives to reduce exposure to the marketing/promotion of discretionary choices in:<br>- B.1.8a out-of-home advertising (billboards, transport vehicles, street furniture, transport hubs such as train stations) within government control?		None found at the time of mapping
	- B.1.8b healthcare settings?		*Healthy Choices Made Easy* (Link) is applicable to all NT Health premises prohibits the promotion of any RED category foods and drinks (those of low nutritional value, e.g. SSBs)
	- B.1.8c other government-controlled buildings/parks?		None found at the time of mapping
	B.1.9 Are there policies limiting the availability/provision of discretionary choices in:<br>- B.1.9a healthcare settings (for visitors and staff)?		*Healthy Choices Made Easy* is applicable on all NT Health premises incl. vending machines, kiosks, fundraising, food trolley and catering. It uses the traffic light system, RED category items are limited to 20% of all available items.
	- B.1.9b buildings, community centres, and parks under government control?		None found at the time of mapping
B.2 Health promotion campaigns	B.2.1 Are there health promotion campaigns (any media type) aimed at encouraging healthy lifestyle behaviours?		At the time of mapping, the *LiveLighter* campaign was on license from the Cancer Council in WA (Link). This program is focused on adults, and not on families or early childhood. The NT has engaged with National campaigns (e.g. Go for 2 and 5; Girls make your move; swap it, don't stop it; Measure Up; Get Moving) but would like more input for adaptability to be locally appropriate.
	B.2.2 Are there health promotion campaigns aimed at developing/supporting healthy food systems and built environments (incl. community-capacity building)?		Under the NPAPH the NT ran a healthy lifestyle community program in collaboration with the SA Obesity Prevention and Lifestyle (OPAL) program 2011-2016. In the NT the program was called *Childhood Obesity Prevention and Lifestyle* (COPAL), and when the NPAPH funding was cut in 2014, the NT was unable to continue to deliver the program nor undertake evaluation of the program. However, Palmerston Council continued with many of the practices and other healthy lifestyle programs (see 2015 Municipal Plan Link)<br>The NT has an online community engagement platform, *Have your say NT* (Link). This platform had a consultation on early childhood, which informed *Starting Early for a Better*

			Future. At the time of mapping there was a public consultation regarding upcoming Planning Reform.
**C. Early childhood education and care (ECEC) settings**		**NT**	
C.1 ECEC settings	C.1.1 Are there support programs for centre-based care settings to encourage healthy food provision? (e.g. management: policies and menu audits; staff: training and resources; families: resources)		At the time of mapping, there was a resource available to support CBC meal provision, *Long Day Care Menu Planner* (Link), and partnerships between Health and Education to engage with the ECEC sector are in Priority Action 1.1 in *The Best Opportunities in Life: Northern Territory Child and Adolescent Health and Wellbeing Strategic Plan 2018-2028* (Link). Interview participants noted that this was a new area for the NT and several programs were expanding across the territory (including FaFT). NAQ Nutrition (Queensland branch of Nutrition Australia, an NGO) offers their *Food Foundations* program into NT ECEC services as a subscription, ~$100/annum (Link).<br>Healthy food policies in school settings exist in most Australian jurisdictions, although such requirements have not been extended to the ECEC sector. The ECEC sector is regulated nationally under the National Quality Framework (NQF) (those regulations are implemented and monitored at a territory level through the education department), so it would make sense to develop these standards in a nationally consistent way. The *feedAustralia* initiative offers support to ECEC sector via an online menu planning tool menu reviews (Link) – those jurisdictions which do not already provide such services could encourage services to access this program.
	C.1.2 Are there programs to support provision of food and physical activity experiences as part of the curriculum?		See C.1.1a
**D. Health (community and tertiary health settings and health promotion activities)**		**NT**	
D.1 Antenatal and birth services	D.1.1 Does antenatal care screen and manage hypertension, hyperglycaemia, appropriate gestational weight gain?		*Clinical Practice Guidelines: Pregnancy Care 2019 edition* (national guidelines) recommend monitoring of blood pressure, weight and screening for hyperglycaemia (Link).<br>NT antenatal checks include blood pressure, blood glucose, and gestational weight gain.
	D.1.2 Antenatal care within public health services:		Most government service delivery in the NT is divided between the Top End (Darwin and Katherine regions and East Arnhem) and Central (Big Rivers, Barkly, Central Australia).<br>Health service delivery is divided between two local hospital networks: Top End Health Service (TEHS) and the Central Australia Health Service (CAHS). TEHS and CHAS are each responsible for hospital care and primary health care in their areas<br>Birthing services are offered in only four hubs across the NT: Darwin, Alice Springs, Katherine, and Nhulunbuy. As a geographically large jurisdiction this means that for many families usual antenatal care is primarily GP-shared care or antenatal services available at the health centres, but hospitals in those hubs but are not accessible for many families who live too far away.
	- D.1.2a Do they include nutrition counselling for healthy pregnancy or are there other healthy lifestyle support programs available during pregnancy?		NT Health funds NGOs to run healthy lifestyle and antenatal programs, and *Child and Family Centres* (Link) offer antenatal services.<br>The *Healthy Pregnancy Healthy Baby Book* (Link) was developed for Aboriginal women in the NT, written in English. It includes information on healthy and safe eating and being active during pregnancy.

	- D.1.2b Is breastfeeding education free (separately or embedded into antenatal education/services)?		Information about breastfeeding is provided at some antenatal services, but it is not clear what information is provided.
	D.1.3 Do maternity facilities fully adhere to the Baby Friendly Health Initiative (based on *Ten Steps to Successful Breastfeeding*)?		The four hospitals with birthing services are all BFHI accredited (Alice Springs Hospital, Gove District Hospital (Nhulunbuy), Katherine Hospital, Royal Darwin Hospital) (Link)
D.2 Early childhood health services	D.2.1 Are there free health/parenting services to support early childhood growth/nutrition (e.g. breastfeeding, complementary feeding, transition to family foods)?		In the postnatal period care moves from midwives' clinic to community clinics. The NT *Child Health Service* (Link) is a service for regular health checks for Mums and children 0-5 years, run in community settings (Link) and remote settings (Link)<br>*Child and Family Centres* were expanding from 6 to 17 at the time of mapping (Department of Territory Families). They offer antenatal services, parent support, early childhood education and long day care, are noted as a key early childhood setting in *The Best Opportunities in Life*.<br>The *Child & Family Service (Central Australia)* (Link) is an integrated service model for under-fives delivered by the Central Australian Aboriginal Congress (Aboriginal Community Controlled Health Service). See 2016 journal article for further information (Link).<br>*Healthy Under 5 Kids* (HU5K) has been a remote program (see Annual Report 2018 (Link)), but at the time of interviews, the program was being reviewed alongside an urban program, which jointly will be scaled up to a territory-wide and universal well child health program. A NT participant noted "I guess lots of opportunities are there for early intervention if problems are picked up" (**NT informant 1**). The HU5K Education Package (Link) is a 2009 resource to support the program. Nutrition information is primarily aimed at the prevention of malnutrition and dental caries.<br>The *Maternal Early Childhood Sustained Home-visiting* (MECSH) program provides in-home sustained support to families who need additional support in early childhood.
	- D.2.1a Is information to support parents readily available (e.g. phonelines, websites)?		None found at the time of mapping
	- D.2.1b Do these include breastfeeding support?		NT government website support for *Breastfeeding services* (Link) defers to the Australian Breastfeeding Association (an NGO).
	D.2.2 Are there healthy lifestyle (education) programs to support families during early childhood?		There are some remote infant feeding programs "Things like the World Health Organization infant feeding programs. So a local version of that's been developed by Fred Hollows in Menzies" (**NT informant 1**).<br>*Families as First Teachers* (FaFT) (Link) is a parent/child early learning and family support program for remote Aboriginal and Torres Strait Islander families offered by the Department of Education in early childhood centres (Commonwealth funding for these centres). "As well as providing support and resources around nutrition, health and hygiene, the FaFT program employs an abecedarian approach, which aims to develop enriched caregiving and quality child-centred early learning experiences - we know this is linked to future health outcomes" (**NT informant 1**).<br>*Territory Parent Support* (TPS) group from 9 weeks postnatally onwards (Link) at the Child and Family Centres (see D.2.1), where "…they've got a great set up for children, and families to come in and participate and engage in an early childhood learning centre as such. And they also

			run programs that will support children and families around healthy food choices or activities" (**NT informant 2**).<br>NGOs are funded to deliver local infant feeding programs, resources for remote communities have been developed e.g. *Feeding Babies* (2013) (Link).
	- D.2.2a Are target populations identified and actively recruited for programs?		The FaFT program at the time of mapping was aimed at remote Aboriginal families across the territory.
	D.2.3 Are Supported Playgroups offered for families that need additional support and do they include healthy lifestyle skills?		*Intensive Supported Playgroups* (Link) support to address underlying family issues. At the time of mapping these programs did not specifically include nutrition and movement advice.
D.3 Workforce	D.3.1 Are there training and resources available for health care professionals to support families?		Training for local health and early childhood education professionals is provided through the HU5K and FaFT programs, "so the public health nutritionists from the strategy would actually provide a lot of education and support to the professional people working in those areas. Because they're not there all the time, they're seeing them probably once every two weeks or something like that. So basically, we're actually building the skills of the workforce a lot of the time and also encourage trying to work with the community around building some programs that they might see as supporting but with the FaFT program that seems to be an in road for a lot of the early education and prevention messages" (**NT informant 2**). At the time of mapping, these were targeted programs expected to be expanded Territory-wide soon.
	- D.3.1a Is preconception advice for nutrition and being active provided to prospective parents?		The *NT Health Nutrition and Physical Activity Strategy 2015-2020* objective 2: promote and support a healthy diet and a healthy weight among women of a childbearing age but does not
	D.3.2 Is there a state/territory health promotion…<br>- D.3.2a …agency (independent or adjunct to health department)?		The Health Promotion Strategy Unit (Link) provides capacity building and training support and contributes to the health promotion evidence base.<br>The *Health Promotion Framework* (2013) (Link) provides guidance for health promotion initiatives to be incorporated into service delivery and health service planning.<br>NT has an Epidemiology Unit to monitor NT population health status (Link). Monitoring systems were under development at the time of mapping: Primary Care Information System (PCIS) and the Community Care Information System (CCIS).
	- D.3.2b …workforce (to implement initiatives locally)?		Rather than having a dedicated workforce to implement programs locally, the NT uses a capacity-building model for local health professionals to carry out health promotion work via the Health Promotion Strategy Unit and also through specific programs, see D.3.1.

2.4 Queensland mapping

Area	Guiding questions	Result	Notes
**A. Governance & leadership**		**Qld**	
A.1 Leadership	A.1.1 Has childhood obesity prevention been identified as a priority by leadership (Premier or Health Minister)?		Yes. At the time of mapping addressing obesity and chronic disease was an identified priority of the Qld government (see priorities from 2017 election progress report, Link). A suite of policies (*Health & Wellbeing Strategic Framework*, *Keeping Queenslanders Healthy*, *Our Future State*) identify increasing the proportion of the population with healthy weight (including children, but not for children under 5 years) as a whole-of-government target (i.e. increase by 10% proportion of population at healthy weight). The WHO Ending Childhood Obesity report is cited in multiple obesity strategy announcements, including taking a whole of government approach.<br>Queensland held an Obesity Summit in 2006, established the Obesity Taskforce (joint led between Health and Sport and Recreation Departments).<br>Queensland Health Minister co-led the National Obesity Summit (February 2019) with the Commonwealth Sports Minister and the Queensland Government led the Council of Australian Governments (COAG, an intergovernmental forum) childhood obesity prevention projects starting from 2016 and was leading the National Obesity Strategy.
	A.1.2 **Key policy/policies**: Is there an overarching policy framework, or a series of key policies or action plans to guide initiatives for the early prevention of obesity in childhood?		At the time of mapping Queensland was in the early stages of developing and implementing policies for obesity prevention, however, there were limitations in the structural support for policy implementation (i.e. no health promotion workforce, the state-wide Children's Health Queensland Hospital and Health Service (HHS) had no obesity prevention strategy aimed at the early years, no direct engagement with the ECEC sector). Policies to support healthy environments were in their infancy.<br>The role of the Healthy Futures Commission (as noted in the *Healthy Futures Commission Queensland Bill 2017* (Link)) is to support action across multiple government sectors, the private sector, NGOs, researchers and the public - enabled through a process of grants from at least 55% of a budget of $20 million across three years and must consider the social aspects of health equity.<br>*Health & Wellbeing Strategic Framework* (2017-2026, Link) (name changed to *Prevention Strategic Framework* in 2020) and the operational document for overweight and obesity, the *Healthy Weight Strategy* (2017-2026) (Link) – aimed at concurrently increasing healthy and reducing unhealthy behaviours through health supportive environments and knowledge/ motivation/attitudes/skills. Healthy public policy key element: identified targets for children increased healthy weight, reduced overweight and obesity, increased physical activity, fruit and vegetable consumption (none for under 5 years). All programs under this policy must be evaluated, and use a specifically developed evaluation framework<br><br>The *Queensland Plan* (Link) is a 30-year plan across a range of metrics and focused on a collaboration across government and *Our Future State: Advancing Queensland's Priorities* (Link),

			are high level overarching long-term plans for Queensland. A third of the work areas in *Our Future State* relate to obesity/chronic disease prevention under 'Give Our Children a Great Start' (includes under 5 years, focus is on development: Target to reduce the proportion of children who are developmentally vulnerable to 22%; Child-focused family support in the early years; universal access to early education (joint with Commonwealth) - Support includes 12 early childhood learning and development services (Pathways for Early Learning and Development) (Link) [media statement]; and, Early Years Places, 50 services hubs across the state, sits within the Early Childhood Education and Care agency (Link)) and 'Keeping Queenslanders Healthy'.<br><br>Informant noted that most strategies are focused on the individual level and attributed this to there being a less clear understanding of environmental elements: "There are a number of strategies that are directed to towards education or skill development of individuals, but not lost in that mix is also the impact of environment, it's just less understood" (**Qld informant**).
	A.1.3 Does the state legislation for public health include prevention/health and wellbeing?		No, but health is a key consideration of the *Planning Act 2016*. Informant noted that regulation is a less desirable pathway: "The legislative approach, they can often be quite lengthy and can happen beyond election cycles or government terms" (**Qld informant**)
	A.1.4 Are their statutory grant-giving bodies with a remit to fund prevention-related community projects?		Not a specific body, but the *Queensland Budget Paper 3* (p.73) (Link) noted several local government grants: Local Government Grants and Subsidies program ($41.7million); Works for Queensland (regional focus) ($147.8 million); Implementing More Effective Funding Grants to Local Government ($1.3 million) – Department of Local Government, Racing and Multicultural Affairs.
A.2 Partnerships	A.2.1 Are partnerships across government noted in 'key policy' identified above?		Yes.
	A.2.2 Are there formal mechanisms for collaborative exchange across sectors (e.g. working groups, policy/outcome joint statements, embedded health positions in agencies outside of health)?		In interview, participant noted that both *Keeping Queenslanders Healthy* and *A Great Start to Life* have intergovernmental mechanisms and the Department of Premier and Cabinet are coordinating cross-government collaboration towards the *Our Future State* priorities. However, the informant noted that more broadly: "It's a bit opportunistic at times and it can be ad hoc, but... Health is clear about the kind of outcomes that we want, and ideas about what other agencies could be doing around supporting Health, and the opportunity to move some of that stuff forward can actually just come in quite random ways and sometimes unexpected" (**Qld informant**)
A.3 Equity	A.3.1 Do the key policies identified outline the structural (incl. social/commercial) causes of obesity?<br>(such as employment/family income, affordable or social housing, adverse early childhood experiences, food security, food systems including promotion, built environment and access to safe/appropriate spaces for being active, etc)		Policies for obesity prevention were fairly new at the time of mapping, several documents and strategies address key upstream areas for prevention, although policy coherence (linking up policies and strategies) is limited.<br>*Our Future State*: High level political document with a range of targets. Economy/creating employment associated with dignity; aim of supporting parents, carers and educators is to "help children better understand healthy choices…" (p.5), the language is aimed personal responsibility rather than environments; 'high quality support throughout pregnancy' to be measured by babies with healthy birth weight (no measures or specifics for the type of antenatal care offered).<br>Skills/training for employment: Investment in training infrastructure at TAFE Queensland; programs to encourage re-entering the workforce - Department of Employment, Small Business and Training.

			Advancing Our Training Infrastructure: *Back to Work*, *Skilling Queenslanders for Work*, *Queensland Budget Paper* (Capital Statement 2018-19, p.53) (Link)<br>Housing: Funding for social housing dwellings (up to 599) - Department of Housing and Public Works; Housing Construction Jobs Program, *Queensland Housing Affordability Strategy*; *Queensland Budget Paper* (Capital Statement 2018-19, p.60) (Link); National Partnership on Remote Housing Funding has ended, new National Housing and Homelessness Agreement commenced in 2018.<br>*State Planning Policy 2017* (Link) and guidance material (Link) outlines that supporting public wellbeing, through 'liveable communities and housing' is a state interest. At the time of mapping, the Minister for Housing and Public Works was also the Minister for Sport, the sport and active recreation strategy/action plan (see B.1.2) crosses over portfolios related to liveability indicators. It covers housing, employment, public open space, public and active transport. Social Impact Assessments (SIA) in addition to Environmental Impact Assessments (EIA) are required. SIAs must ensure that health and the wellbeing of the community (local and regional) are considered, potential negative impacts are minimised, and opportunities for improvement are maximised.<br>*Regional Planning Interests Act 2014* (Link) sets outs areas of regional strategic interest for social, economic and environmental prosperity. There are no references to Aboriginal or Torres Strait Islander (nor use of the term Indigenous) cultural interests.<br>Funding for safe and clean water for Indigenous communities - Department of Local Government, Racing and Multicultural Affairs - Indigenous Councils Critical Infrastructure Program for water, wastewater and solid waste infrastructure - Queensland Budget Paper 3 (Capital Statement 2018-19) (p.73) infrastructure grants for environmental health ($0.965 million) and water, wastewater and solid waste ($50 million) (Link)<br>Transparency for engagement with lobbyists: Lobbyists Register requires state and local government and opposition representatives to keep details of contacts with lobbyists for 10 years. The register has mandatory reporting and real-time transparency to try to limit commercial influence on policy (Link).
	- A.3.1.a Do recommendations for action/initiatives address these structural causes?		The recommendations have strong potential to address structural causes, but as these are relatively new policy areas it is too soon to tell if the policies that will be implemented will achieve their stated goals
	A.3.2 Are target populations (with higher risk of developing obesity) identified for additional support?		Fairly limited acknowledgement of target populations relating to actions. Prevalence data suggests inner regional and remote areas have much higher incidence of obesity than major cities in adults (no difference for children) and no statistical difference for Aboriginal and/or Torres Strait Islander adults (but children were significantly less likely to be obese); socioeconomic status: children in disadvantaged areas 2.5 times more likely to be obese than children in most advantaged areas (*Health & Wellbeing Strategic Framework* p.10)
**B. Environments in which we live (e.g. work, shop, eat, be active and play)**		**Qld**	
B.1 Health supportive environments	B.1.1 Do planning policies orientate built environments towards principles of active living?		New policy area. Queensland amended the *Planning Act 2016 (Qld)* (Link) and its associated *Guideline* (Link) and *Planning Regulation 2017* – other jurisdictions were attempting similar planning reform around the same time, but many did not pass through the legislature.

			State Planning Policy 2017 outlines that supporting public wellbeing, through liveable communities is a state interest (see A.3.1 and Queensland's new planning system (Link)) The Planning Act requires (and enables) local government to consider the wellbeing of the community; local governments are supported through the *Active Health Communities* initiative (see B.1.7). *QDesign: Principles for good urban design in Queensland* (Link) is a guideline. Developed by the Queensland Government Architect (with a foreword from the Minister of Housing and Public Works/ Sport) it contains nine principles including street connectivity, climate responsive, creating spaces for people to live. A strategy to encourage prioritising the "needs of the children and elderly" in order to respond to the diversity of community needs. "If proposed housing options, land use activities, parks, streets and transport options respond to the specific needs of the young and the elderly, it is more likely to accommodate the whole community throughout life" (p. 25)
	B.1.2 Are there investments for public infrastructure (e.g. footpaths, bikeways, or greenspaces) to encourage being active?		New statewide policy areas. Queensland Budget Paper 3: $20.2 million for cycle network development across the state (p. 122) (Link). The *Queensland Cycling Strategy* (Link) and *Queensland Walking Strategy* (Link) are being led by Department of Transport and Main Roads (a shift away from local government). Queensland has a sport and active recreation strategy led by the Minister for Sport, who is also the Minister for Housing and Public Works (high cross-over portfolios for liveability indicators) – these are strategy: *Activate! Queensland 2019-2029* (Link) and action plan *Our Active8 2019-2022* (Link). These are supported by the Health Department.
	B.1.3 Are there food/nutrition policies aimed at ensuring a nutritious, affordable, accessible food system? (e.g. incentivise local food production or increase healthy food access in disadvantaged communities, zoning policies, or incentives to retailers)		There were no specific food/nutrition policies. There was some policy scaffolding in agricultural and planning sectors. Several long-term planning and audit policy documents support a whole-of-supply chain approach to agriculture. Annual audits are conducted update an agricultural investment tool *Queensland Agricultural Land Audit* (Link); *Queensland food and fibre policy* (Link) State planning policy focus on agriculture, *State Planning Policy - state interest guideline - Agriculture, 2016* (Link) - Department of Agriculture and Fisheries; Department of infrastructure, Local Government and Planning. Regional Plans sit under the State Planning Policy (SPP) suite of land use planning and development - there are two regions where regional planning has a focus on agriculture – Regional Planning Interest Act 2014. At the same time a long-term strategic framework for the agricultural sector identifies five megatrends in global agribusiness: 1) interconnected global value chains and food supply, 2) greater global wealth and desire for convenience, 3) streamlined food system chains, 4) climate change, **5) food for health** in *Queensland Agriculture and Food Research, Development and Extension 10-Year Roadmap and Action Plan* (Link).
	B.1.4 Are there programs to support vendors to improve food offerings in food outlets (restaurants, cafes, take-away, vending machines)?		None found at the time of mapping
	B.1.5 Is nutrition information at food outlets (menu board labelling) required by legislation?		In 2017 Queensland amended the Food Act to align with New South Wales policy (i.e. 20 or more stores in Queensland or 50 or more stores nationally) - *Fast Choices* (Link).

			Queensland led the consultation for Food Standards Australia New Zealand (FSANZ) to consider regulating for nationally consistent menu labelling initiatives and broadening menu labelling schemes – i.e. to go beyond the listing of kilojoule labelling alone.
	B.1.6 Is there engagement with food retail (supermarkets, grocers, corner stores, etc) to reduce the availability and promotion of discretionary choices in-store?		*Active Healthy Communities* supports local governments to engage with local food retailers although at the time of mapping there were limited case studies to showcase success.<br>*Healthy Communities Project Pilot* (Link) Cape York partnership with Traditional Owners and Elders, Mayors, and Councillors of three Cape York communities to reduce sugar-sweetened beverage availability and consumption (18 month pilot)
	B.1.7 Are local governments empowered to encourage health-supportive environments?		To support the Planning Act 2016, state government has supports for local governments to encourage health supportive built environments. *Active Healthy Communities* (Link) is a resource for local government includes promotion of healthy foods in partnership with local food outlets and growers, limiting access to unhealthy food outlets, and leveraging the built environment to improve food environments. It also encourages locally driven projects to improve walkability and other built environment/planning considerations for being active. There are 'how to' instructions for implementing *Breastfeeding and baby care facilities code* (Link) and recommendations for using local law (e.g. food licensing, food stall sizes, etc) to improve local food offerings across multiple settings (Link).<br>After a period of local government reform in Queensland, regulatory changes (Link) require the reporting of potential conflict of interest in local government (and the explicit prohibition of any donations by property developers) (Department of Local Government, Racing and Multicultural Affairs).<br>Queensland has 77 LGAs (Link) and two local government Acts: Local Government Act 2009 Brisbane City Act 2010
	B.1.8 Are there any initiatives to reduce exposure to the marketing/promotion of discretionary choices in:<br>- B.1.8a out-of-home advertising (billboards, transport vehicles, street furniture, transport hubs such as train stations) within government control?		The Queensland government banned discretionary food and drink promotion on all government-owned advertising spaces (including Queensland's rail network, buses, bus shelters, roadsides, and outside major hospitals) – *Advertising content on Queensland Government advertising spaces* & *Policy Guideline: Unhealthy food and drink including alcohol* (Link)
	- B.1.8b healthcare settings?		Health Service Directive *Healthier Drinks at Healthcare Facilities* (March 2019), applying to all HHSs, informed an amended Directive to also include food (although not completed/released until 2020, Link)
	- B.1.8c other government-controlled buildings/parks?		*Advertising content on Queensland Government advertising spaces* (see B.1.8a) – scope extends to advertising spaces owned or positioned on land/an asset owned by a Queensland Government agency/entity, including common areas (e.g. lifts or foyer) but it excludes retail outlets and their 'footprint' within government property.
	B.1.9 Are there policies limiting the availability/provision of discretionary choices in:<br>- B.1.9a healthcare settings (for visitors and staff)?		Health Service Directive: *Healthier Drinks and Healthcare Facilities* (June 2019) and *Healthier drinks at healthcare facilities best practice guide* (2016) required the removal of sugar-sweetened drinks from Queensland's 16 Hospital and Health Services (HHS) – a network of locally

			administered health services. At the time of mapping, this directive applied to unhealthy drinks only and not to unhealthy food (although policy development was underway at the time of mapping).<br>*Update*: just outside of mapping scope (and so 'result' remains policy scaffolding), a new policy suite was introduced, influenced by the COAG Health Council project to reduce children's exposure to unhealthy food and drinks. *A Better Choice – Healthy Food and Drink Supply Strategy for Queensland Health Facilities* supported by the *A Better Choice*: food and drinks classification guides (Link). This uses a traffic light system like the ACT initiative.
	- B.1.9b buildings, community centres, and parks under government control?		No statewide public sector policy at the time of mapping.<br>A guideline policy *Be Healthy, be safe, be well* (Link) outlines 'Better health' as a key pillar for public sector workplaces and *Healthier. Happier. Workplaces.* (Link) provides organisations with support to improve food environments and could serve as the basis for policy development in this space.
B.2 Health promotion campaigns	B.2.1 Are there health promotion campaigns (any media type) aimed at:<br>- B.2.1a encouraging healthy lifestyle behaviours?		*Healthier. Happier.* (Link) – campaign drives public back to a website with sections on fitness, food and tools for behaviour change in addition to a social marketing activity primarily driven through Facebook and Instagram. This advice is mostly focused on school-aged children.
	- B.2.1b developing/supporting healthy food systems and built environments (incl. community-capacity building)?		*Active Healthy Communities* has a community engagement and social inclusion strategy to encourage participation, e.g. community-led audits of neighbourhood walkability and community-supported local food systems.<br>*Get involved* (Link) is an online platform used by the Queensland Government to consult with the public. The platform was used to consult with the public for the development of the 20-year long-term state policy, *The Queensland Plan* (referenced in this policy mapping).<br>A capacity-building and skills-development program is funded by government for Queensland Country Women's Association branch members, who then deliver healthy lifestyle programs in their communities, e.g. *QCWA Country Kitchens* (Link)
**C. Early childhood education and care (ECEC) settings**		**Qld**	
C.1 ECEC settings	C.1.1 Are there support programs for centre-based care settings to:<br>- C.1.1a encourage healthy food provision? (e.g. management: policies and menu audits; staff: training and resources; families: resources)		At the time of mapping no consistent approach to sector engagement in Queensland, nor funding for specific projects in terms of provision or curriculum.<br>Some examples exist that could be built upon for broader engagement. Under the NPAPH, the Queensland government invested in the development and evaluation of the LEAPS program (Learning, Eating, Active Play, Sleep), delivered by NAQ Nutrition (the Queensland branch of Nutrition Australia, an NGO) (Link). When that funding period ended the ongoing support for the sector moved to NAQ under the *Food Foundations* program, who offer similar sector supports for a subscription (i.e. each service pays a fee of ~$100/year) (Link), and provide resources such as *Menu Planning in Queensland ECEC settings*. It seems unlikely that the government will step in to secure funding for this program, as the Queensland participant noted that "The idea of having a fairly rock solid, well-funded strategy approach in Queensland ... I'm just not seeing that at the moment" (**Qld informant**).

			Children's Health Queensland HHS also provides some professional training and engages with the Education Department in relation to schools (and so there are some potential opportunities to support Authorised Officers (who assess ECEC services against national regulation)).<br>*Smart Choices* policy developed by Health Department and aligns with National Healthy School Canteen Guidelines, monitored by Education Department. Health funds QAST (Queensland Association of School Tuckshops, an NGO) to support public and private schools to implement, so not a lot of cross over with the ECEC sector. Compliance is around 50%.<br><br>Healthy food policies in school settings exist in most Australian jurisdictions, although such requirements have not been extended to the ECEC sector. The ECEC sector is regulated nationally under the National Quality Framework (NQF) (those regulations are implemented and monitored at a state/territory level through either the education or communities departments), so it would make sense to develop these standards in a nationally consistent way. The *feedAustralia* initiative offers support to ECEC sector via an online menu planning tool menu reviews (Link) – those jurisdictions which do not already provide such services could encourage services to access this program.
	- C.1.1b provide food and physical activity experiences as part of the curriculum?		See C.1.1a
**D. Health (community and tertiary health settings and health promotion activities)**		**Qld**	
D.1 Antenatal and birth services	D.1.1 Does antenatal care screen and manage hypertension, hyperglycaemia, appropriate gestational weight gain?		*Clinical Practice Guidelines: Pregnancy Care 2019 edition* (national guidelines) recommend monitoring of blood pressure, weight and screening for hyperglycaemia (Link)
	D.1.2 Antenatal care within public health services:		
	- D.1.2a Do they include nutrition counselling for healthy pregnancy or are there other healthy lifestyle support programs available during pregnancy?		A support program/couching service available via telephone, includes 10 sessions: *Get Healthy in Pregnancy* – licenced from NSW (Link).<br>In the *Queensland Plan* a focus area called *Give our Children a Great Start* highlights the importance of a healthy birth weight (target to increase the proportion of delivered babies at healthy birth weight to 5% by 2025).<br>*Growing Good Habits* – website for families (Link)
	- D.1.2b Is breastfeeding education free (separately or embedded into antenatal education/services)?		Queensland Health Clinical Guidelines *Establishing Breastfeeding* (Link) note that information about anticipatory guidance for breastfeeding should be supplied at each antenatal visit and develop a breastfeeding plan in partnership with the patient, and to offer a referral to a lactation consultant if risk factors identified. Included in this plan development is to respect the decision of the mother should she decide not to breastfeed (and to document in pregnancy health record for the benefit of other health practitioners)
	D.1.3 Do maternity facilities fully adhere to the Baby Friendly Health Initiative (BFHI) (based on *Ten Steps to Successful Breastfeeding*)?		The BFHI is accredited through the Australian College of Midwives (ACM). The ACM have confirmed the *Establishing Breastfeeding* Queensland Clinical Guidelines meets the standards of BFHI Australia. While the central Health Department and the associated clinical guidelines endorse HHSs (Link) seeking BFHI accreditation, it is up to the 16 independent HHSs to pursue accreditation – 15 public hospitals had BFHI accreditation in Queensland.

D.2 Early childhood health services	D.2.1 Are there free health/parenting services to support early childhood growth/nutrition (e.g. breastfeeding, complementary feeding, transition to family foods)?		The Child Health Service (CHS) (Link) available around the Greater Brisbane area for children from birth to 8 years of age (**geographically limited**). All HHSs offer some version of this program although they can have different names for the same type of service. For early childhood – Key age child health checks; drop-in clinics (up to 12 weeks, supports early infant feeding and parental concerns); infant feeding and parent support program (birth to six months, appointments and referral required for this more in-depth service, only available at some CHS) (Link).<br>Additionally, the Personal Health Record (Red Book) to record health appointments, development and growth checks.<br>Parenting groups for parents with infants vary in the age range offered, depending on the HHS, e.g. from birth to three months of age (Link), or birth to five months and 5-12 months (Link).<br>*(*Update 2021, *Early Years Places* (Link) and funding for *Neighbourhood and community centres* (Link) have been established across the state and include health services and parenting support).
	- D.2.1a Is information to support parents readily available (e.g. phonelines, websites)?		Online materials for *Healthy lifestyle resources for consumers* (Link) is a directory page for self-referral into the Get Healthy Queensland (Link) services, *Healthier. Happier.* (Link) website, and other information about diet/nutrition (Link) and fitness/exercise (Link). *Growing Good Habits* (families) has support on early child feeding
	- D.2.1b Do these include breastfeeding support?		*Growing Good Habits* (families) does not have maternal support/advice for breastfeeding.
	D.2.2 Are there healthy lifestyle (education) programs to support families during early childhood?		None beyond the (geographically limited) Child Health Service.<br>There are cooking programs available through the *Country Kitchens* (Queensland Country Women's Association) (Link) and *Jamie Oliver: Ministry of Food* (The Good Foundation), for a fee ($50-150) (Link) programs
	- D.2.2a Are target populations identified and actively recruited for programs?		The available cooking programs (D.2.2) are promoted as regional/rural programs.<br>Programs are available for Aboriginal and Torres Strait Islander families (see D.3.1a). In addition, Good Start is a program aimed at *Good Start to Life* supports Māori and Pacifika families, in the Logan area (referenced in the Logan Community Health Action Plan 2017 (Link).<br>*Making Tracks towards closing the gap in health outcomes for Indigenous Queenslanders by 2033: Investment Strategy 2015-2018* (Link)<br>A healthy start to life is one of five priorities, includes improving health literacy and reproductive health of young women and services which are culturally appropriate for antenatal, infant, child and maternal services and parenting support.
	D.2.3 Are Supported Playgroups offered for families that need additional support and do they include healthy lifestyle skills?		Supported Playgroups include a trained facilitator to engage families and provide support for families, they can be offered by outreach for families/communities in remote locations (Link).<br>They are offered on an ad hoc basis across the state.
D.3 Workforce	D.3.1 Are there training and resources available for health care professionals to support families?		Children's Health Queensland (a statewide Hospital and Health Service) developed *Project ECHO®* (Link) a free online training resource for health professionals across a range of health areas, included the Childhood Overweight and Obesity ECHO Network.<br>Website with *Healthy Lifestyle resources for health professionals* (Link) includes links to nutrition education resources, maternal and infant nutrition, referrals into programs and training, and link to consumer website *Growing Good Habits*.<br>*Brief intervention for a healthy lifestyle* (Link) training for general population and maternity and child health clinicians from the Queensland Health's Clinical Skills Development Service.

			A range of Professional development opportunities in maternal and infant nutrition (Link) include brief interventions, knowledge assessment tools for breastfeeding initiation<br>The *Queensland Child and Youth Clinical Network* (QCYCN) (Link) is a statewide network of researchers, clinicians, educators and consumers interested in improving healthcare for children in Queensland<br>*Growing Good Habits* – website for health professionals (Link)<br>*Health Workforce Strategy for Queensland* (Link); *Aboriginal and Torres Strait Islander Health Workforce Strategic Framework* (Link). Equitable access to health services, prevention and early intervention has 'significant' emphasis.
	- D.3.1a Is preconception advice for nutrition and being active provided to prospective parents?		*Get Healthy* service is a 10-session coaching service available across Queensland (on licence from NSW). It has two programs that would service the adult population in the pre-conception phase: standard program; Aboriginal and Torres Strait Islander program (both explore elements relating to improved nutrition, physical activity and maintaining a healthy weight). In addition, *Deadly Choices* Healthy Lifestyle program supports initiatives in Central, North West and Far North Queensland.
	D.3.2 Is there a state/territory health promotion…<br>- D.3.2a …agency (independent or adjunct to health department)?		The Healthy Futures Commission led to the 2017 election promise to establish a statutory public health commission with the key remit of addressing chronic disease and obesity across the life course. At the time of mapping the legislation to establish the agency *Health & Wellbeing Queensland* had passed, but was not yet enacted.<br>Population monitoring: CATI survey for self-reported anthropometry, health status, health behaviours and access to healthcare. Continuous data collection target 12,500 adults and parents as proxies for 2500 children aged 5-17 years (*Preventive health surveys* Link)
	- D.3.2b …workforce (to implement initiatives locally)?		It was an election promise in 2017 to rebuild the health promotion capacity across the state via new health promotion agency, *Health & Wellbeing Queensland* (see D.3.2a). Although it was not clear if this agency will have a workforce capable of implementing programs and initiatives at the local level. The former health promotion workforce was embedded into the 16 HHS's, but almost all positions were lost after the 2012 Queensland election (see this article for more information (Link)). Children's Health Queensland is a HHS based in Brisbane, with a state-wide remit but no prevention projects were identified at the time of mapping.

2.5 South Australia

Area	Guiding questions	Result	Notes
**A. Governance & leadership**		**SA**	
A.1 Leadership	A.1.1 Has childhood obesity prevention been identified as a priority by leadership (Premier/First Minister or Health Minister)?		An election was held in March 2018. An election campaign for Liberal party (who had formed government at time of mapping): *Better Prevention for a Healthy South Australia* (Link) focused on healthy lifestyle behaviours, children were mentioned but there was little detail available (Update 2021, this election campaign is not reported on)
	A.1.2 **Key policy/policies**: Is there an overarching policy framework, or a series of key policies or action plans to guide initiatives for the early prevention of obesity in childhood?		At the time of mapping a new government had just been formed. Priorities and policies were being reconsidered at the time of mapping and interview, but key areas for early childhood (notably the ECEC sector and how young children are impacted by the wider environments in which they live) were absent.<br>Population health considerations are embedded into processes within the public health planning system which encourage and support collaboration across government (vertical and horizontal), with the public and private sector. Several key pieces of policy informed the policy space in SA at this time.<br>The *South Australian Public Health Act 2011* requires prevention to be considered in public health administration (see A.1.3); the newly elected government campaigned on obesity prevention (see A.1.1); the *draft State Public Health Plan 2019-2024* (Link) available at the time of mapping; and the established mandate for addressing the determinants of health through their Health in All Policies (HiAP) approach all indicate strong policy infrastructure for obesity prevention in SA. However, there was no specific overarching approach for childhood obesity prevention in SA at the time of mapping.<br>HiAP (Link) started in 2007 with a mandate from the Premier, establishing an authorising environment for cross-government work across a range of health areas. It started with a 'Thinker in Residence' proposal: "to consider how South Australia could better promote health and wellbeing… We held a conference at the end of [the] residency, which the Premier opened… [and it] endorsed SA trialling a HiAP approach" (**SA informant**). The HiAP team was a small unit within the Health Department and built capacity and networks over time. In 2009 and a re-commitment in 2014 from leaderships to HiAP happened through memorandums of understanding (MOUs), the latter happened through the auspices of the 2011 public health act implementation in 2014. Methodology for engagement (learning by doing approach) and for applying HiAP thinking directly to policy issues (Health Lens Analysis model) were developed. Another form of cross government work came under the *Change@SA 90 Day Projects* model (Link) which seconded policy officers from a range of agencies to co-define problems and their solutions on a pre-defined policy area. A change of government could potentially shift the policy infrastructure in place, however, the *State Public Health Plan* released late 2018 re-endorsed the HiAP approach.

			The *State Public Health Plan 2019-2024* (SPHP, 2018) (Link) is SA's second Public Health Plan. Its vision is "A healthy, liveable and connected community" (p.4). Partnerships are emphasised as the cornerstone of the plan – across government agencies, between state and local government, through public health partnerships, and with business and community organisations. Within that policy harmonisation across government is emphasised (see Appendix 3) and focus for action is often devolved to local government, Public Health Partner Authorities (formal partnerships to collaborate with government and deliver actions to improve population health and wellbeing), public health partners (government or NGO whose core business impacts on population health and wellbeing). The SPHP guides the Regional Public Health Plans that local governments must develop and notes the previous regional plans had many initiatives relating to nutrition and physical activity. The SPHP focuses more on the ageing population, although it notes an aim for 'all ages friendly communities' and commits to support health and wellbeing in specific settings but the ECEC sector is not included (schools, workplaces, community spaces, health settings and prisons) (p.28). The plan identifies the establishment of a prevention agency, Wellbeing SA, to have governance and oversight for the implementation of the SPHP.<br>The *Health & Wellbeing Strategy* (consultation document) (Link) takes a risk factors approach and is heavily focused on acute, medical treatment.
	A.1.3 Does the state legislation for public health include prevention/health and wellbeing?		*South Australian Public Health Act 2011* (Link)<br>SA has a Chief Public Health Officer whose function is to protect and promote public health, which under this Act includes wellbeing and prevention. The Act defines public health to include prevention of chronic disease.<br>A series of principles that guide the collaborative, population-focused, preventive approach to public health in SA are set out in the Act. Section 7: proportionate regulation principle (health promotion regulation should minimise impact on business); Section 8: considerations for impacts on future generations; Section 9: principle of prevention; Section 10: population focus principle (actions should be aimed at the population, and in turn the health of individuals); Section 11: Participation principle (people should have opportunities to participate in public health decisions); Section 12: Partnership principle (12(1) 'The protection and promotion of public health requires collaboration and, in many cases, joint action across various sectors and levels of government and the community'); Section 13: Equity principle (requires equity to be considered).<br>Section 26: Established the South Australian Public Health Council – 10 members including the Chief Public Health Officer, 2 with local government experience, 1 with public health qualifications with local government experience, 2 with qualifications in public health, 1 with environmental protection experience in local government, 1 with health promotion experience, and 1 with experience in communicable disease control.<br>Section 37(1) 'A council is the local public health authority for its area' and is responsible for health promotion in their community and to partner with other councils.<br>The Act requires the development of a State Public Health Plan (Section 50) and Regional Public Health Plans (developed by councils/local government, Section 51) (see also Link).

			Under Section 51 councils are enabled to establish Public Health Partner Authority agreements with third parties to deliver/implement strategies to meet priorities in their regional public health plans (see also Link)
	A.1.4 Are their statutory grant-giving bodies with a remit to fund prevention-related community projects?		Not at the time of mapping – *Wellbeing SA* was in the process of being established when mapping was being undertaken and was intended to have such a remit as one of its roles (Update 2021, established January 2020, Link)
A.2 Partnerships	A.2.1 Are partnerships across government noted in 'key policy' identified above?		Partnerships are identified but they are broad (and not linked to specific actions) in the *State Public Health Plan 2019-2024* (Appendix 1), see also the draft SPHP (Link), Appendix 4: SA Health Commitments which did not make it into the final SPHP) – recognition of the partnerships required to address multiple determinants of health – a renewed commitment to the continuance of HiAP both with across government and non-government agencies (business sector/industry and not-for-profit/community sector) to address health inequities and social determinants of health. The informant noted to toll of frequent restructuring: "I don't know if you're aware but we're being restructured about every 18 months and we're about to be restructured again [now that] we've had a new government [elected]" (**SA informant**).
	A.2.2 Are there formal mechanisms for collaborative exchange across sectors (e.g. working groups, policy/outcome joint statements, embedded health positions in agencies outside of health)?		*SA Public Health Act* enables collaborative work through Section 12 (the partnership principle). The purpose of HiAP is to develop cross-sectoral collaboration and to develop public health lens skills among public policy officers. The *State Public Health Plan* endorses the HiAP methodologies and governance, which include a cross-government project team and executive oversight (approval/sign off via individual agency structures for decision making, then through senior officers' group and onto cabinet). An informal Community of Practice has emerged from collaborative working processes of HiAP – capacity-building activities are led by the Strategic Partnerships team (Link). Additional methodology includes *90 Day Projects* (Link) which seek to create understanding across a range of agencies in complex or highly political issues, and develop productive relationships across government but also with the community. Examples include 'State of Wellbeing'.<br>The Act and the SPHP also enable collaboration between state-local-national government, and Public Health Partner Authorities.
A.3 Equity	A.3.1 Do the key policies identified outline the structural (incl. social/commercial) causes of obesity? (such as employment/family income, affordable or social housing, adverse early childhood experiences, food security, food systems including promotion, built environment and access to safe/appropriate spaces for being active, etc)		SA Health includes the Department for Health and Ageing, the Local Health Networks, and the SA Ambulance Service. *SA Health Strategic Plan* 2017-2020 and *Early Actions Plan* (Link), In the draft/consultation version of the *SA Health & Wellbeing Strategy*, the health sector takes a risk factors approach (rather than a determinants approach) to health. This is likely due to the McCann Review (Link) and the loss of funding and staff for non-hospital services, and the shift towards partnerships to deliver on prevention in SA.<br>Non-hospital health policies do identify the structural causes of obesity, and other chronic conditions. The HiAP approach starts by examining the determinants of health. Housing, social connectedness, built and food environments, active transport, and climate change all feature in the *Chief Public Health Officer's Report 2014-2016* (Link) and the 2018 SPHP. SA has an ageing population, 75% population live in capital city and surrounds (Adelaide region) although most of the state is very remote, with a high burden of disease. At the centre of the determinants

			approach is a focus on partnerships across government, between state-local agencies, and with community organisations and businesses.<br>A part of the HiAP methodology, a *90 Day Project* on food security involved engagement with emergency food relief sector and clients, here they found that people were reliant on emergency food relief services for long periods of time, often dipping in and out of service use throughout any given year, but that the sector was not aware of this long term use: "We undertook qualitative research with both food security clients and the sector to get a really good understanding around what's going on. And I think the sector is trying to move because they hadn't understood that people are getting stuck and they're becoming dependent and they can't get out. And so we really worked how we could change the sector and we're probably about to start working on guidelines for nutrition. It probably won't meet the dietary guidelines, because I don't think it is possible for food security services at this point to be able to do that, but we are going to work them up" (**SA informant**).
	- A.3.1.a Do recommendations for action/initiatives address these structural causes?		*South Australian Public Health Act 2011* – Section 13 contains the equity principle, requiring consideration be given to reduce health disparities between population groups (see A.1.3. for more information on the Act).
	A.3.2 Are target populations (with higher risk of developing obesity) identified for additional support?		The *State Health Plan 2019-2024* (Appendix 2) recognises the variability in health needs for some population groups in SA: Aboriginal people (*SA Aboriginal Chronic Disease Consortium Road Map for Action*), people living in rural and remote areas, people experiencing socioeconomic disadvantage. Targeted programs to improve public health as well as interventions to improve health supportive environments in key settings for priority populations (e.g. programs to improve foods and beverages made available). Specific programs are not noted in this plan.
**B. Environments in which we live (e.g. work, shop, eat, be active and play)**		**SA**	
B.1 Health supportive environments	B.1.1 Do planning policies orientate built environments towards principles of active living?		The *Planning, Development and Infrastructure Act 2016* (Link) initiated significant planning systems changes in the state, still in flux at the time of mapping. It influenced how the South Australian Planning Strategy was developed to have a focus on land use but also public space and transport infrastructure. Health aligned their messages around being active and social inclusion with connection to nature, climate change mitigation and protection of natural assets and biodiversity and in doing so developed a partnership with the environment department.<br>"We're also undergoing really significant changes to our planning legislation and our planning system at the moment [but the impact of that] really depends on how well the rule book is developed… The Environment Department and the Health Department are working together to present a united voice to the Planning Department [who are drafting the new planning guidelines]. We're trying to help shape and inform the way they do it, but they've got lots of needs to balance. Because the lobbyists for large developments and housing corporations are very powerful in here… [so we aimed to] help shape and inform the way they do it… [by increasing] the focus on Healthy Liveable Neighbourhoods" (**SA informant**).<br>The South Australian Planning Strategy includes a requirement for the seven planning regions of SA to develop long-term planning strategies called *Regional Plans* (Link) including land use,

			transport infrastructure and public space. Planning regions are: Greater Adelaide (represents 75% of the population), Eyre and Western, Yorke Peninsula and Mid North, Far North, Kangaroo Island, Limestone Coast, and Murray Mallee.<br>*The Planning Strategy for South Australia: 30 Year Plan for Greater Adelaide* (Link) represents 75% of SA population. It has six targets that align to many of the liveability indicators (increasing density and minimising urban sprawl (links to protection of agricultural land), proximity to public transport, active transport infrastructure, greater housing choice, increased urban green cover, walkable neighbourhoods) and 14 policy themes, including health, wellbeing and inclusion. Under this policy theme sits the 'Healthy Neighbourhoods' elements (housing variety, access to sport and recreation facilities, green streetscape, 800m to shops, 400m to public open space, 400m to bus/800m to train/light rail, 1km to school or childcare, 5km to 'employment zoned' land.<br>The SA participant noted the *30 Year Plan* included "all kinds of environmental triggers that we know encourage activity across human interaction. Which are protective of how to prevent obesity and so on" (**SA informant**).
	B.1.2 Are there investments for public infrastructure (e.g. footpaths, bikeways, or greenspaces) to encourage being active?		There was no specific physical activity plan at the state level.<br>*Regional plans* require consideration of transport infrastructure, land use and public space.<br>*Building Stronger South Australia* 2013 (Link) was an integrated transport and land use plan between transport and planning departments and features public transport, cycling and walkability.<br>Increasing the proportion of residents that cycle was a government priority, there was also a HiAP Health Lens Analysis Project *A Whole of Government Approach to the Development of the South Australian Government Cycling Strategy* (Link). The Department of Planning, Transport and Infrastructure had a Cycling and Walking Section as well as several community programs orientated towards road safety and multiple modality (Link). Community cycling grants are available annually to create bike paths for local councils. The network of cycling routes is called Bike*direct* and there is an interactive journey planner available *Cycle Instead* (Link).<br>The *State Public Health Plan 2019-2024* reported (p.19) that public health actions in the previous Regional Public Health Plans were predominantly focused on encouraging physical activity.<br>*Healthy Parks, Healthy People South Australia* 2016-2021 (updated 2021, Link) commenced in 2016 under a Public Health Partner Authority Agreement with health and Department of Environment – focused on the health and wellbeing benefits of a community that is connected to nature and parks and in turn the need to protect these public assets. It also partners with range of stakeholders in urban planning, social inclusion, education, primary industries, and Aboriginal-controlled organisations. Additionally, the provision of greenspaces features heavily in the *30 Year Plan*.
	B.1.3 Are there food/nutrition policies aimed at ensuring a nutritious, affordable, accessible food system? (e.g. incentivise local food production or		There were no specific food/nutrition policies at the state level.

	increase healthy food access in disadvantaged communities, zoning policies, or incentives to retailers)		A range of food related policy levers for healthy weight targets were identified in the *Healthy Weight: A Desktop Analysis* report in 2012 (Link) including: food and beverage manufacturing, marketing, retail, catering/procurement, community spaces.<br>Regional Public Health Plans were activities were less focused on nutrition-focused activities compared to other activities (such as physical activity). Those activities included health literacy, social connection, healthy food availability – reported in the *State Public Health Plan 2019-2024* (p.19)<br>SA introduced a *Food Waste Levy*, applicable to any supermarket or food retail dumping food. This levy motivated a reduction in food waste but it also led to retailers dumping high volumes of ultra-processed foods into the emergency food relief sector (note: food dumping happens in all jurisdictions). SA undertook *at 90 Day Project* on food security engaging with emergency food relief providers and clients to help improve nutritional output in that area, “It won't probably meet the dietary guidelines, because I don't think it is possible for food security services at this point to be able to do that, but we are going to work them up” (**SA informant**).
	B.1.4 Are there programs to support vendors to improve food offerings in food outlets (restaurants, cafes, take-away, vending machines)?		Under the NPAPH, the Obesity Prevention and Lifestyle (OPAL) program engaged with some councils to support local businesses to improve their food offerings (Link). A taskforce in 2015 set out to improve food offerings available for children (Link), and the *Healthy Kids Menu Initiative* came out of these (Link). The program supports cafes, pubs, restaurants, clubs and hotels to improve their food offerings for children, indicating the healthier option on the menus and will eventually have a website where families can locate participating venues.
	B.1.5 Is nutrition information at food outlets (menu board labelling) required by legislation?		Yes, in 2017, amendments to SA Food Regulations were made, which aligned to the NSW policy (Labelling of kilojoule information in chain food outlets Link) – this work was brokered through the COAG Health Council Obesity Working Group.
	B.1.6 Is there engagement with food retail (supermarkets, grocers, corner stores, etc) to reduce the availability and promotion of discretionary choices in-store?		Not at the time of mapping
	B.1.7 Are local governments empowered to encourage health-supportive environments?		Regional Public Health Plans (RPHPs) are required by each local council in SA (68) under the SA Public Health Act and strategically driven by the State Public Health Plan (see Link). At the time of mapping the first cycle of RPHPs were coming to an end. Some councils develop these plans as groups – e.g. the Eastern Health Authority represents the public health elements of five local council areas and they have planned to develop their next RPHP jointly.<br>At the same time, planning decisions have been taken away from local councils. Currently there are no policy instruments available for local governments to limit the number of businesses selling predominantly unhealthy foods.
	B.1.8 Are there any initiatives to reduce exposure to the marketing/promotion of discretionary choices in:<br>- B.1.8a out-of-home advertising (billboards, transport vehicles, street furniture, transport hubs such as train stations) within government control?		At the time of mapping there were no policies in place, however, it was a policy area under consideration at the time of interviews. The SA participant identified a perceived barrier to developing such a policy: "the Transport Department may temporarily lose funds if they do a lot of advertising in unhealthy food and drink on public transport vehicles and bus stops – this is one of the areas we hope to investigate through the HiAP whole of government processes. So I think it's early days in this space, but we've got the approval to go through the government

			cabinet sub-committee process to test this through consultation and we're very heavily relying on our Health in All Policies experience. Some of our colleagues in Cabinet Office help us navigate this space, which is helpful. But, yeah, so come back to us in a year and I'll let you know how we've gone" (**SA informant**)
	- B.1.8b healthcare settings?		The Healthy food and drink choices in SA facilities (see B.1.9) applies to marketing practices in the same settings
	- B.1.8c other government-controlled buildings/parks?		Not at the time of mapping
	B.1.9 Are there policies limiting the availability/provision of discretionary choices in:<br>- B.1.9a healthcare settings (for visitors and staff)?		The *Healthy food and drink choices in SA Health facilities* (Link) started in 2008. The policy is mandatory and applies to all health services and facilities (including hospitals, community centres, health department offices, etc) where any food and beverages are provided. Outlets include kiosks, cafeterias, cafes, vending machines, shops, catering (meetings, training, functions, education programs) and sponsorship, fundraising and advertising.
	- B.1.9b buildings, community centres, and parks under government control?		Not at the time of mapping
B.2 Health promotion campaigns	B.2.1 Are there health promotion campaigns (any media type) aimed at:<br>- B.2.1a encouraging healthy lifestyle behaviours?		*Be active* (Link) encourages being active across the life course, many of the Healthy Parks, Healthy People promotions encourage being active also. There were no campaigns to support healthy eating
	- B.2.1b developing/supporting healthy food systems and built environments (incl. community-capacity building)?		Several programs were finishing or had ended at the time of mapping.<br>A skills-development program for volunteer community members to act as 'agents of change' – SA *Community Foodies* (Link) was at the end of the funding cycle at the time of mapping and potentially moving out to NGO sector.<br>A previous program – Obesity Prevention and Lifestyle (OPAL, Link), delivered in 21 local councils (including one in NT) was funded under the NPAPH; *SA Public Health Act* changes have focused on the role of and relationship with local governments in population health and Public Health Partnerships, there may be new opportunities to engage at this level.<br>*Healthy Workers – Healthy Futures* program was funded to 2018 (i.e. the time of mapping) to support healthy workplaces and prevent chronic disease (Link), providing a range of suggestions for workplaces to improve the health of their workforce (Link). It included supporting businesses/workplaces to take up practices to support breastfeeding through the Australian Breastfeeding Association, with their *Breastfeeding Friendly Workplace Accreditation* program.<br>Work around developing healthy 'food cultures' had happened in the past with the agricultural department, but nothing recently<br>Community capacity building opportunity through community consultation processes: *YourSAy* (Link) is an online portal for community members to comment on public policy, e.g. draft planning and design code consultation. The *State Public Health Plan* notes that many metropolitan and regional public health plans have initiatives to "build community voice in

			council planning and decision making on local matters, including regional public health planning governance" (p.20)
**C. Early childhood education and care (ECEC) settings**		**SA**	
C.1 ECEC settings	C.1.1 Are there support programs for centre-based care settings to:<br>- C.1.1a encourage healthy food provision? (e.g. management: policies and menu audits; staff: training and resources; families: resources)		Not at the time of mapping<br>The formerly government funded and delivered *Start Right Eat Right* program provided guidelines and resources to support services with food provision in long day care, or centre based care (CBC) such as the *Menu Assessment and Planning Guidelines for Long Day Care Centres*. It also has an award scheme to motivate ECEC sector participation, showing that the program was effective at improving nutrition intake for children aged 2-4 years (Study, Link) It was defunded as a result of the McCann Review into non-hospital spending by the health department. The SA participant noted strong relationships with the ECEC sector through this program. "We used to have a really good relationship with them in the past and we had some really great *Start Right, Eat Right* guidelines and there was a lot of work with the people that provide kids food, like the caterers that do all the food provision in childcare settings" (**SA informant**).<br>Healthy food policies in school settings exist in most Australian jurisdictions, although such requirements have not been extended to the ECEC sector. The ECEC sector is regulated nationally under the National Quality Framework (NQF) (those regulations are implemented and monitored at a state level through the education department), so it would make sense to develop these standards in a nationally consistent way. The *feedAustralia* initiative offers support to ECEC sector via an online menu planning tool menu reviews (Link) – those jurisdictions which do not already provide such services could encourage services to access this program.<br>(Update 2021. Since initial mapping, *Healthy Communities Program* has commenced (Link) it includes resources for the ECEC sector and parents of children under five (Link); *Wellbeing SA* has partnered with the Healthy Eating Advisory Service to support services to support provision of healthy meals in CBC (subscription with Nutrition Australia Victoria))
	- C.1.1b provide food and physical activity experiences as part of the curriculum?		None at the time of mapping
**D. Health (community and tertiary health settings and health promotion activities)**		**SA**	
D.1 Antenatal and birth services	D.1.1 Does antenatal care screen and manage hypertension, hyperglycaemia, appropriate gestational weight gain?		*Clinical Practice Guidelines: Pregnancy Care 2019 edition* (national guidelines) recommend monitoring of blood pressure, weight and screening for hyperglycaemia (Link). These tests are routinely undertaken in GP shared care and public hospital care options in SA.
	D.1.2 Antenatal care within public health services:		
	- D.1.2a Do they include nutrition counselling for healthy pregnancy or are there other healthy lifestyle support programs available during pregnancy?		An additional program to existing antenatal services. SA subscribes to and funds the *Get Healthy in Pregnancy* service for SA residents (Link), a lifestyle coaching program initially developed in NSW (see SF1b)

	- D.1.2b Is breastfeeding education free (separately or embedded into antenatal education/services)?		*South Australian Perinatal Practice Guidelines: Breastfeeding* (Link) identifies discussing woman's intentions for breastfeeding and identifying any potential barriers to successful breastfeeding at first antenatal visit (or at first opportunity), with subsequent visits to provide information about attachment, feeding cues and frequency, and avoidance of supplementary feeding unless medically required. Additionally, antenatal classes are held at all public hospitals in South Australia, who decide what content to present. Support for breastfeeding is provided at the Child and Family Health Service (part of the Women's and Children's Health Network) (Link)
	D.1.3 Do maternity facilities fully adhere to the Baby Friendly Health Initiative (BFHI) (based on *Ten Steps to Successful Breastfeeding*)?		A policy directive requiring all maternity services to attain BFHI accreditation could not be found during mapping. However, the majority of public hospitals and community centres (e.g. Child and Family Health Service) are BFHI accredited in SA (17 services including 10 hospitals) (Link). The *South Australian Breastfeeding Program* was developed in 2005, now hosted and Women's and Children's Health Network for SA Health. This program offers the *Baby Friendly Online Education Program* (Link) for a fee to specialist ($66), medical ($11) and general ($6) staff, and it meets the 8 hour education requirement for BFHI accreditation. It is housed on the SA Health eLearning hub, *Launch* (see D.3.1)
D.2 Early childhood health services	D.2.1 Are there free health/parenting services to support early childhood growth/nutrition (e.g. breastfeeding, complementary feeding, transition to family foods)?		*Child & Family Health Service* (CaFHS) (Link) offer health services by appointment and also offers 'drop-in' clinics, open office hours on weekdays. There are >70 service sites across the state and support families with children aged 0-5 years with breastfeeding (and formula feeding issues), introduction to solids, nutrition, etc.<br>Health Checks are offered to families at the CaFHSs at five time points: initial (1-4 weeks), 6 weeks, 6-9 months, 18-24 months and the Preschool Health Check. Opportunities exist for brief interventions for breastfeeding, introduction of solids (incl. timing) and supporting parents to provide appropriate foods and beverages.<br>CaFHS also offers *Early Parenting Groups* (4 weeks to 4 months of age)
	- D.2.1a Is information to support parents readily available (e.g. phonelines, websites)?		The CaFHS website has information on pregnancy, early childhood, children and adolescent, and family health (Link), *Parent Helpline* offers support 24/7 on behaviour, nutrition, child health and parenting (Link), and *Parenting SA* website to support parenting skills (Link)
	- D.2.1b Do these include breastfeeding support?		Face-to-face services at CaFHS offer breastfeeding support in addition to some hospitals<br>Specialised services for infant feeding available to 12 months of age for families requiring additional support (residential placement, requires referral)
	D.2.2 Are there healthy lifestyle (education) programs to support families during early childhood?		None found at the time of mapping
	- D.2.2a Are target populations identified and actively recruited for programs?		n/a
	D.2.3 Are Supported Playgroups offered for families that need additional support and do they include healthy lifestyle skills?		Supported Playgroups exist but no ongoing support available. Some resources have been provided for the *Learning Together Program* (Link)
D.3 Workforce	D.3.1 Are there training and resources available for health care professionals to support families?		SA Health has the *Launch* online platform (Link) to deliver a range of eLearning training programs for health professionals in a range of topic areas, including BFHI accreditation for

			individual health professionals. Currently there are no training modules for the early prevention of obesity in childhood.
	- D.3.1a Is preconception advice for nutrition and being active provided to prospective parents?		There is some simple advice for women about pre-pregnancy diet, supplements, etc on the *Pregnancy* section of the Women's and Children's Health Network website (Link) although it is unlikely this on its own would translate into proactive preconception care advice for prospective parents
	D.3.2 Is there a state/territory health promotion…<br>- D.3.2a …agency (independent or adjunct to health department)?		At the time of mapping the health promotion agency *Wellbeing SA* was being developed (Link). In the *Health & Wellbeing Strategy* (consultation document), it was noted the purpose of *Wellbeing SA* is to provide integrated services across care models (and life course), develop intermediate care services, "enhance efforts in relation to health promotion, prevention, screening and early intervention activity [and] Continue to provide, high value programs focussed on risk factors relating to the development of chronic disease and tailored to meet the needs of specific groups" (p.23). There was a *90 Day Project* on wellbeing (to achieve a shared understanding of wellbeing across multiple agencies) (Link).<br>The *South Australian Indicator Framework* has potential to provide evidence of impact for policies in health supportive environments overtime. In line with *SA Public Health Act 2011* equity is the focus of indicators, there are plans to develop/review 'as new data sources become available' (see *State Public Health Plan*, p.48)<br>Population monitoring: *South Australian Population Health Survey* (Link) target 7000 all ages, parents as proxies for children under 16 years.
	- D.3.2b …workforce (to implement initiatives locally)?		Despite having the SA Public Health Act there was a limited health promotion workforce at the time of mapping; a consequence of the 2013 McCann review (see Link) was the abolition of the Health Promotion Branch of SA Health. At the time of mapping *Wellbeing SA* was under development and it was unclear if the agency would have the funding or operational capacity to rebuild and develop such a workforce. The Act requires local councils to develop regional plans for public health and wellbeing, supported by the 5-year SA State Public Health Plan. The state's 68 local councils have varying capacity and capabilities to deliver programs (or to fund partners to deliver programs).

2.6 Tasmania

Area	Guiding questions	Result	Notes
**A. Governance & leadership**		**Tas**	
A.1 Leadership	A.1.1 Has childhood obesity prevention been identified as a priority by leadership (Premier/First Minister or Health Minister)?		A government goal is to make Tasmania the healthiest population by 2025 by addressing obesity and smoking (see Healthy Tasmania in A.1.2). Tasmania was one of four jurisdictions to submit to the Senate Inquiry into the Obesity Epidemic in Australia. In their submission, *The Tasmanian Government submission to Senate Select Committee into the obesity epidemic in Australia* (submission 144, July 2018, Link) focused on the need to address childhood obesity at all levels of government and across government agencies. The informant noted the need to be ready for the opening of policy windows: "…different persuasions of political groups are supportive of different things and we have to make the most of a situation at the time" (**Tas informant**).
	A.1.2 **Key policy/policies**: Is there an overarching policy framework, or a series of key policies or action plans to guide initiatives for the early prevention of obesity in childhood?		The *Healthy Tasmania Five Year Strategic Plan* (Link) outlines Tasmania's approach to prevention, adopting whole-of-government strategies including nutrition and physical activity. The plan identifies specific areas for early years (*Child Health and Parenting Service* (see D.2.1) and *Healthy Kids Tasmania* website (see D.2.1a)) and recognised the Health-in-all-policies approach used in South Australia (Link). Like many jurisdictions, the health promotion workforce in Tasmania is limited, and *Healthy Tasmania* outlines the preventative health commissioning model used to outsource some health promotion activities, as well as a partnership model to work across government.<br>Delivery of the five year strategic plan is supported by *Healthy Tasmania Fund* (Link), *Healthy Tasmania Community Innovation Grants* (Link) and *Healthy Tasmania* portal (Link), *Healthy Tasmania Community Forum* (Link), and the annual *Healthy Tasmania Neighbour Day Challenge* (Link). Cross-government interventions focus on those in the built environment to encourage physical activity:<br>• Encourage use of parks and reserves<br>• Low-cost promotion of active tourism<br>• Investment in environment, planning, and facilities by Department Premier and Cabinet and State Growth to support active recreation, sport, and physical activity<br>• Expansion of bus services to encourage more mixed-mode active/public transport<br>Whereas nutrition-based interventions are community-level strategies:<br>• Healthy workplaces in government settings (largest employer in Tasmania)<br>• Food access and food cooperatives (and emergency food relief)<br>• Mapping of prevention activity across health and community in Hospital and health care services and Community Health Plans<br><br>The *Premier's Health and Wellbeing Advisory Council* (formerly Premier's Physical Activity Council) (Link) and a seconded health position to the Department of Premier and Cabinet to work on liveability in partnership with some of the larger local governments, using a Health-in-All-Policies lens.

			Tasmania's Strategy for Children – Pregnancy to Eight Years 2018-2021 (Link) and *Early Years in Tasmania* (Link) take an ecological model of human development, and cover a range of programs and services for parents and the ECEC sector, it is led by the Department of Education. This work is linked in with Health and it pivots around the 12 *Child and Family Centres* (different to the 66 Child Health Centres, run by Department of Health and Human Services (DHHS) – see D.2.1), and kindergarten (see also *Working Together for 3 Year Olds* (Link)). The Early Years are also considered in *Healthy Tasmania* Plan and the *Child and Youth Wellbeing Framework* (Link) (DHHS).
	A.1.3 Does the state/territory legislation for public health include prevention/health and wellbeing?		The *Public Health Amendment (Healthy Tasmania) Bill 2017* (Link) updated the public health act to consider preventive health, although as a tool has only been used to deliver on specific actions to reduce smoking among Tasmanians. "We do have a Public Health Act and it does involve some things around the need to have preventive health in it, but it hasn't been used, particularly. We've used it a lot for the tobacco stuff, but not so much for legislation around what I would consider obesity prevention initiatives at this stage. But I think there is room for exploring that further" (**Tas informant**).<br>It remains a potential avenue for other aspects of prevention such as nutrition and physical activity, where changes to the planning legislation encourage consideration of health and wellbeing.
	A.1.4 Are their statutory grant-giving bodies with a remit to fund prevention-related community projects?		Not a specific grant-giving body. Between 2017/18 – 2018-19 there was $1 million fund available for community grants. A new program, the *Healthy Tasmania Community Innovation Grants* (Link) will have $6.6 million available over two years, with each grant of up to $200,000 (which can be spent over up to three years). The informant noted the idea behind these grants was similar to 'safe-to-fail' experiments: "…throwing a certain amount of funding out there [to see what 'sticks'], which is sort of what we're trying to do with our Healthy Tasmania innovation grants (although our grants are too small to be effective)" (**Tas informant**).
A.2 Partnerships	A.2.1 Are partnerships across government noted in 'key policy' identified above?		While a partnership model was identified in key documents, there were very limited actual partnerships identified at the time of mapping. Those that were identified in these documents were mostly with the education department and some support for the development Community Action Plans as well as small community grants (seed funding).
	A.2.2 Are there formal mechanisms for collaborative exchange across sectors (e.g. working groups, policy/outcome joint statements, embedded health positions in agencies outside of health)?		At the local level there are a range of supports (see B.1.7) including mapping of activities, but less so across state government. The informant noted support for Health in All Policies in principal, but felt that the language was not appropriate in Tasmania: "Even the language around Health in All Policies, to me is, even though I completely support what it is it's trying to do, the language is wrong… which I think can be really off putting" (**Tas informant**).
A.3 Equity	A.3.1 Do the key policies identified outline the structural (incl. social/commercial) causes of obesity?<br>(such as employment/family income, affordable or social housing, adverse early childhood experiences, food security, food systems including promotion, built environment and access to safe/appropriate spaces for being active, etc)		The key prevention policies are focused on personal responsibility, i.e. education and information to increase knowledge and change behaviours, rather than health supportive environments. They do not make the link to the structural causes of obesity. The *State of Public Health Report* 2018 (Link) identifies a range of structural causes of obesity and chronic disease that did not directly cross over into the key prevention policy documents.<br>Despite not being acknowledged in the key prevention policies there are a range of non-health policies that consider structural determinants of health.<br>The Tasmanian Government has a priority to increase the population and grow the economy. Tasmania has a dedicated department to support economic growth, the Department of State Growth, a key policy is the *Population Growth Strategy* (Link). The departments priority is to increase the population to

			650,000 by 2050 under three pillars: job creation and workforce development, migration, and liveability. The latter of these include a range of cross-government strategies<br>• Vibrant communities (community events; hospitality industry; creative industry/ cultural attractions; infrastructure) – Dept State Growth<br>• Work-life balance for families (Employer of Choice program; policies to encourage flexible work for parents/carers; out of school care; affordable childcare) – Dept State Growth, Dept Premier & Cabinet, Dept Education<br>• Encourage migration to Tasmania (offer support services and community integration) – Dept Premier & Cabinet, Dept State Growth<br>• Inclusive communities (multicultural inclusion) – Dept Premier & Cabinet<br>An unintended consequence of encouraging migration (and tourism) to Tasmania – to grow the economy – has resulted in housing shortages for low income households. To address this, *Tasmania's Affordable Housing Strategy 2015-2025* (Link) (Department of Communities) focuses on increasing the supply of social housing and supported accommodation. The second supporting action plan, *Affordable Housing Action Plan 2019-2023* (Link) adds $125 million on top of $73.5 million from Action Plan 1 (delivered at outlined) to deliver total supply of 2400 affordable lots and homes, assistance to 3600 households. This plan would impact on approximately 1.5% of Tasmanian households.<br>Tasmania developed a *Food and Nutrition Policy* in 2004 (see B.1.3) and "… had a Food Security Strategy, back in 2010" (**Tas informant**) – these considered a range of factors including the economy, agriculture, and emergency food relief, but were not current at the time of mapping.<br>The 12 *Child and Family Centres* (see D.2.1) administered by the Department of Education are the site of a new service delivery model for the early years (pregnancy through to five years). Co-locating service delivery from across government including Education (management) and other services including health, social work and community agencies as well as linking into other locally available services based on family need.<br>Recognition of historical violence and enduring systems of racism have underpinned to key policies in Tasmanian Reconciliation. A priority of Department of Premier and Cabinet, *Resetting the relationship with the Tasmanian Aboriginal Community* (Link), included amendments to Tasmania's Constitution to recognise Tasmanian Aboriginal people as the First People of Tasmania (attaining Royal Assent on 15 December 2016). Additionally, the *Cultural Respect Framework for Aboriginal and Torres Strait Islander Health 2016-2026* (Link) (DHHS) was developed from the findings of a community consultation.
	- A.3.1.a Do recommendations for action/initiatives address these structural causes?		The non-health policies identified in A.3.1 do address some of the structural causes of obesity, but the approach to these determinants at the state-level is ad hoc.
	A.3.2 Are target populations (with higher risk of developing obesity) identified for additional support?		Priority populations in Tasmania are identified as low-income households and Aboriginal Tasmanians.

B. Environments in which we live (e.g. work, shop, eat, be active and play)		Tas	
B.1 Health supportive environments	B.1.1 Do planning policies orientate built environments towards principles of active living?		*Land Use Planning and Approvals Amendment (Tasmanian Planning Policies and Miscellaneous Amendments) Act 2018* (Link) was part of a trend across Australian jurisdictions to update their planning legislation and policy. In Tasmania, this tapped into the recognition of the role of planning to deliver (specifically via the *Tasmanian Planning Policies* (TPPs) to relate to 'liveability, health and wellbeing of the community' (Link). TPPs are subordinate to State Policies (*Tasmanian Sustainable Development Policies*) and relate specifically to land use. Further information about the Tasmanian Planning Commission about the planning reforms (Link). Guidelines were developed for built environment and physical activity considerations in Tasmania by the Heart Foundation, *Healthy By Design* (Link).<br>The Population Growth Strategy (Link). A state priority is to increase the population to 650,000 by 2050 – underpinned by three pillars: job creation and workforce development, migration, and liveability. Investment is geared towards families: community building events (community/cultural/creative industry events, multicultural inclusion); work-life balance that supports flexible working conditions and affordable childcare; encouraging migration with support services and community integration.<br>The informant noted a range of activities around liveability were being undertaken between state and some local governments: "We have trialled a few things with some of the larger councils, and there's a lot of work going on… around liveability" (**Tas informant**).
	B.1.2 Are there investments for public infrastructure (e.g. footpaths, bikeways, or greenspace) to encourage being active?		In Tasmania the department of sport and recreation explicitly recognises population physical activity as their core business, reflected in their name – Department of Sport, Recreation and Physical Activity. Tasmania is one a few jurisdictions to have a physical activity plan, *Tasmania's Plan for Physical Activity 2011-2021* (Link), it was developed by the Premier's Physical Activity Council (Update 2021, that name has since changed). The long-term plan is supported by implementation plans (3 years) and annual action plans. This policy links in with the *Get Moving Tasmania* (Link) campaign, driven by the Department of Premier and Cabinet. A range of resources are available for local governments (Link) including: *Healthy by Design* guidelines (Heart Foundation); *Neighbourhood Walkability Checklist* (Heart Foundation); *Recreation Planning Manual* (Department of Communities, Sport and Recreation).<br>Tasmanian government priority to increase the population and grow the economy, part of that is to improve aspects of liveability (Department of State Growth). The *Tasmanian Urban Passenger Transport Framework* (Link) 'supports improved accessibility, liveability and health outcomes for our communities'. Additionally, policies on land use and infrastructure to encourage walking and cycling are outlined in the *Tasmanian Walking and Cycling for Active Transport Strategy* (Link).<br>The informant felt there was renewed interest in transport for active living partnerships: "When it comes to the transport [department], there's a whole lot of active living stuff going on at the moment" (**Tas informant**).
	B.1.3 Are there food/nutrition policies aimed at ensuring a nutritious, affordable, accessible food system? (e.g. incentivise local food production or		Tasmania was the only jurisdiction to have a food and nutrition policy. The *Tasmanian Food and Nutrition Policy* was developed in 2004 (Link), touches on the economy, health and disease prevention, food safety, food security, and food environments. A Progress Report (Link) in 2009

	increase healthy food access in disadvantaged communities, zoning policies, or incentives to retailers)		indicated the policy drove action across government sectors, it secured funding for the Tasmanian Food Security Council, and influenced the sustainability lens brought to the *Tasmania's Sustainable Agri-Food Plan 2016-18* (Link). While many of the health department actions still flow out of the spirit of *the Food and Nutrition Policy*, it is now largely defunct. Attempts were made to re-develop the plan in 2014, but "Then there was all the cutbacks from the [NPAPH], but then our Minister decided that he wanted to do *Healthy Tasmania Strategic Plan*, so the timing was wrong to re-develop it and there'd been a big contraction with our partners in state growth and economic development and agriculture. They'd lost a lot of staff. Basically, to get them to buy in to the obesity prevention space was a bit tricky at that point. We've put it to bed temporarily" (**Tas informant**).<br>The Tasmanian Government is seeking to grow the agricultural sector in Tasmania, which provides opportunities for increasing local access to core foods as well as food-based tourism, there is overlap with the goals of the Food and Nutrition Policy. See *Tasmania's Sustainable Agri-Food Plan 2016-18* (Department of Primary Industries, Parks, Water and Environment). The growth of the agricultural sector is supported by the Department of State Growth (Link) and planning considerations protect agricultural land from urban sprawl.
	B.1.4 Are there programs to support vendors to improve food offerings in food outlets (restaurants, cafes, take-away, vending machines)?		None found at the time of mapping
	B.1.5 Is nutrition information at food outlets (menu board labelling) required by legislation?		No, additional laws in Tasmania were not deemed necessary as large outlets (i.e. national chains) decided to roll out consistent menu labelling in all jurisdictions and a scoping study found that going through the lengthy legislative process would only apply to one additional bakery.
	B.1.6 Is there engagement with food retail (supermarkets, grocers, corner stores, etc) to reduce the availability and promotion of discretionary choices in-store?		*Eat Well Tasmania* (see B.2.1b) engages with food retail. It also seeks to increase local farmers markets in rural areas.<br>Major supermarket stocking practices reduce the freshness of locally produced fresh produce in Tasmania, as the food is first shipped to Melbourne, sorted, and then sent back to local shops in Tasmania.
	B.1.7 Are local governments empowered to encourage health-supportive environments?		Tasmania has 29 local governments for a population of half a million. While the smaller councils only employ a handful of people, the larger councils (in and surrounding Hobart and Launceston) have greater capacity to engage in health promotion activities (e.g. the Huon Valley Council Health and Wellbeing Strategy (Link), 2018). Some of the larger local government areas are engaging with the department of premier and cabinet around the HiAP work in the 'liveability' or built environment space. Tasmania also has the *Local Government Forum* (Link) which is hosted by the Department of Sport, Recreation and Physical Activity.
	B.1.8 Are there any initiatives to reduce exposure to the marketing/promotion of discretionary choices in:<br>- B.1.8a out-of-home advertising (billboards, transport vehicles, street furniture, transport hubs such as train stations) within government control?		None found at the time of mapping
	- B.1.8b healthcare settings?		None found at the time of mapping

	- B.1.8c other government-controlled buildings/parks?		None found at the time of mapping
	B.1.9 Are there policies limiting the availability/provision of discretionary choices in:<br>- B.1.9a healthcare settings (for visitors and staff)?		None found at the time of mapping
	- B.1.9b buildings, community centres, and parks under government control?		None found at the time of mapping
B.2 Health promotion campaigns	B.2.1 Are there health promotion campaigns (any media type) aimed at:<br>- B.2.1a encouraging healthy lifestyle behaviours?		Some of the *Eat Well Tasmania* messages are focused on promoting healthy lifestyles, i.e. *Get Fruity* and *Veg it Up* (see B.2.1b).<br>*Healthy Tasmania Challenge* (Link) an annual social marketing campaign.<br>The *LiveLighter* campaign was being used in Tasmania on licence from the WA program of the same name. This program was aimed at adults, and at the Tasmanian participant noted that the program would not be re-funded (Link). Some other programs aimed at adults are in workplaces, i.e. *Ritualiz* (Link), and for physical activity, i.e. *Get Moving Tasmania* (Link)
	- B.2.1b developing/supporting healthy food systems and built environments (incl. community-capacity building)?		*Eat Well Tasmania Inc* (Link) is an NGO that champions healthy eating (aimed at all ages). A full-time position is funded by the health department, primarily to network and build inter-sectoral collaboration to connect food producers, retailers, manufacturers, food service outlets, with local communities to try to grow a local food culture, and promotes messages for eating healthy produce in season. Campaigns include *What's in season*, *Veg it up*, *Get fruity*, and *Local food procurement* (Link).<br>The *Tasmanian Healthy Families Food Coalition* is a partnership between Eat Well Tasmania, Tasmanian School Canteen Association, and the Child Health Association (Link), takes a food systems approach to improve food literacy across the community (ECEC and school settings, families, and food retailers and producers).<br>The *Breastfeeding Coalition Tasmania* (Link) receives government funding, membership includes not-for-profits, health professionals, health care organisations (incl. hospitals), government departments, local councils, Aboriginal organisations, and universities. Its main aim is to advocate for supportive environments for breastfeeding.<br>A range of community capacity building programs exist in Tasmania:<br>Skills-development program to help parents to provide healthy meals, led by Volunteer Family Food Educators – *Family Food Patch* (see D.2.2.) "What we do is, we train people in what healthy eating and physical activity is, but how can you, as an advocate in your community, make a difference. And we link them together with all the other programs that are going on… we really ground them in physical activity guidelines and dietary guidelines, so that they actually understand. We try and weed out the rubbish… We train them in how to work in that space, as an informal advocate" (**Tas informant**)<br>Several forums for community consultation: *Healthy Tasmania Community Forum* (Link); and the *Local Government Forum* (hosted by the Department of Sport, Recreation and Physical Activity) (Link)<br>The Heart Foundation has developed the *Neighbourhood Walkability Checklist* (Link) to encourage communities to identify ways of improving their built environments for health.

C. Early childhood education and care (ECEC) settings		Tas	
C.1 ECEC settings	C.1.1 Are there support programs for centre-based care settings to:<br>- C.1.1a encourage healthy food provision? (e.g. management: policies and menu audits; staff: training and resources; families: resources)		Ongoing support is provided to the sector through the *Move Well Eat Well - Early Childhood* program (Link) which has been running since 2012, this is based on Victoria's *Kids – Go for your life program*. The service is provided by the Health Department who also provides small teams of dietitians (program had approximately five full-time equivalent positions to manage program) to review service menu's and supported by the *Healthy Kids Coalition* (led by NGOs working in child nutrition) who act in an advisory role and share/develop resources. The program is supported by the *Move Well Eat Well Early Childhood Services Menu Planning Guidelines*.<br>Health and Education have a good relationship, developed relationships and trust over 20 years, e.g. ongoing funding provided for a school canteen program has helped to embed healthy eating in schools and curriculum.<br>Healthy food policies in school settings exist in most Australian jurisdictions, although such requirements have not been extended to the ECEC sector. The ECEC sector is regulated nationally under the National Quality Framework (NQF) (those regulations are implemented and monitored at a state level through the education department), so it would make sense to develop these standards in a nationally consistent way. The *feedAustralia* initiative offers support to ECEC sector via an online menu planning tool menu reviews (Link) – those jurisdictions which do not already provide such services could encourage services to access this program.
	- C.1.1b provide food and physical activity experiences as part of the curriculum?		The *Move Well Eat Well – Early Childhood* service is provided by the Health Department who also provides small teams of dietitians and physical activity officers to provide curriculum topics in addition to curriculum resources
**D. Health (community and tertiary health settings and health promotion activities)**		**Tas**	
D.1 Antenatal and birth services	D.1.1 Does antenatal care screen and manage hypertension, hyperglycaemia, appropriate gestational weight gain?		*Clinical Practice Guidelines: Pregnancy Care 2019 edition* (national guidelines) recommend monitoring of blood pressure, weight and screening for hyperglycaemia (Link).<br>At around 10-14 weeks gestation, pregnant women are provided with a *Maternity Information Package* pamphlet (Link) outlining all the services available to support them during pregnancy, birth, and early parenting. Health check-ups are scheduled throughout pregnancy and highlight when tests for BMI, hypertension, and screening for gestational diabetes will happen during usual care.
	D.1.2 Antenatal care within public health services:		
	- D.1.2a Do they include nutrition counselling for healthy pregnancy or are there other healthy lifestyle support programs available during pregnancy?		Emotional wellbeing and healthy lifestyle advice, including being active and additional nutrition requirements are embedded into the antenatal check-ups at 7-10 weeks, 10-14 weeks, 16-20, 24-28, 32, 34, 36, 38, and weekly from 40 weeks as required. In addition to these health check-ups there are also group based antenatal classes, informal sessions covering physical, emotional and mental preparation for pregnancy are parenthood. It is not clear if specific nutrition or physical activity counselling is available during group based antenatal classes. Continued exercise during pregnancy and dietary advice (along with weight gain recommendations) are both within the *Maternity Information Package*.
	- D.1.2b Is breastfeeding education free (separately or embedded into antenatal classes)?		The Tasmanian Health Service (THS) Women's Adolescents and Children's Service's (WACS) offers free antenatal classes from about 26 weeks gestation and free breastfeeding workshops from about 34 weeks gestation – details are available in the *Maternity Information Package*.

	D.1.3 Do maternity facilities fully adhere to the Baby Friendly Health Initiative (based on *Ten Steps to Successful Breastfeeding*)?		All THS hospitals have BFHI accreditation.
D.2 Early childhood health services	D.2.1 Are there free health/parenting services to support early childhood growth/nutrition (e.g. breastfeeding, complementary feeding, transition to family foods)?		The *Child Health and Parenting Service* (CHaPS) (Link) is a model of care offering a range of services for parents. The service offers age/life stage health and development checks at 2 weeks, 4, 8 weeks, 4-6 months, 12 months and 2 years, in addition to the (4 years) Preschool-ready *Healthy Kids Checks* (Link), services are provided by child and family health nurses and no referrals required. CHaPS services are offered at the *Child Health Centres* (Link). In addition to CHaPS, the *Child Health Centres* also support parents in drop-in style information provision on topics such as breastfeeding, infant and toddler nutrition, play, etc. at 66 centres across the state.<br>Additionally, 12 *Child & Family Centres* (Link) were established by the Education Department in areas of high disadvantage as a whole of government response to deliver child and family needs in a holistic (rather than co-located) way, i.e. taking an ecological view of the child to deliver services such as community development, education, and health. The *Child & Family Centres* were co-designed with communities (centres are also supported by the *Move Well Eat Well* program, see C.1)<br>(Update 2021: The Tasmanian *Child & Family Centres* influenced SA who took up this model since initial mapping with their 42 (and growing) *Children's Centres* (Link).
	- D.2.1a Is information to support parents readily available (e.g. phonelines, websites)?		Additional resources for families relating to pregnancy, baby, and early childhood (1-5 years) are available on the *Healthy Kids Tasmania* website (Link), see summary page of parent resources (Link).<br>Of note, the *Start Them Right* guide (Link) for transition to solids has step by step guidance on portions, textures and food groups (with pictures) to support parents with early feeding.<br>The Parent Line offers support 24/7 for parents (call costs apply) (Link).
	- D.2.1b Do these include breastfeeding support?		Antenatal breastfeeding workshops and postnatal support services are available – details are provided in the *Maternity Information Package*. Additional support is provided through the *Healthy Kids Tasmania* website (Link) and the national breastfeeding support phoneline as well as at drop-in services at CHaPS.
	D.2.2 Are there healthy lifestyle (education) programs to support families during early childhood?		There were no specific lifestyle programs found at the time of mapping, however *Child & Family Centres* (see D.2.1) are a potential setting to deliver these types of specialised programs.<br>The *Family Food Patch* program (Link) was originally developed in 2001, to meet an identified need to address food security issues in Tasmania (see a 2010 summary of the program, Link). Its funding shifted from state to national (under the NPAPH, until 2015), then delivery moved out to a not-for-profit organisation called Child Health Association Tasmania. This organisation provides workshops or information sessions for families. The *Family Food Patch* program trains *Family Food Educators*, who live and volunteer in the community a promote nutrition and being active – focusing on areas of disadvantage and rural. (Update 2021, the Child Health Association Tasmania is now called Families Tasmania).
	- D.2.2a Are target populations identified and actively recruited for programs?		*Family Food Patch* (see D.2.2) is aimed at areas of disadvantage and rural communities
	D.2.3 Are Supported Playgroups offered for families that need additional support and do they include healthy lifestyle skills?		None found at the time of mapping, although some Child & Family Centres (se D.2.1) do offer playgroups and take a holistic approach to meet child/family needs so such services may be provided on an ad hoc basis.

D.3 Workforce	D.3.1 Are there training and resources available for health care professionals to support families?		The *Healthy Tasmania Portal* (Link) has some resources for health and community workers, including a link to *Health Learning Online* for online training opportunities (Link).<br>The health department consider skill development and capacity building of health professionals their core business: "…basic information provision and skill development of the workforces. We actually invest quite a bit of energy internally in skill development of people like family and child health nurses, aged care nurses, maternity services nurses, and other professionals, who can spread the message. They're just sort of core business for us and always have been and the information provision on the website type sort of stuff, all the hand outs and fact sheets and all that stuff" (**Tas informant**)
	- D.3.1a Is preconception advice for nutrition and being active provided to prospective parents?		None found at the time of mapping
	D.3.2 Is there a state/territory health promotion…<br>- D.3.2a …agency (independent or adjunct to health department)?		The health promotion workforce capacity within the health department is limited. The Tasmanian approach is to partner with community organisations (both government community health organisations and not-for-profit organisations). The *Healthy Tasmania* website was designed as a 'one stop shop' to house supportive resources for health and community workers, information and tools relating to the preventive health plan.<br>*Population monitoring*: The *Tasmanian Population Health Survey* was last survey in 2016 (Link), next due in 2019 (Link), CATI survey of 6300 Tasmanian adults, questions include diet and physical activity, socio-economic data, and environmental health and wellbeing items.
	- D.3.2b …workforce (to implement initiatives locally)?		Within the health department there is a limited health promotion workforce, where the focus is on capacity building of health professionals in contact with families (maternal services nurses, child health nurses, "who can spread the message" (**Tas informant**). Many programs are run out of *Neighbourhood Houses* under a community development model; *Family Food Patch* is delivered by an NGO but funded by the Health Department; the 12 *Child & Family Centres* (see D.2.1) have a community development remit.

2.7 Victoria

Area	Guiding questions	Result	Notes
**A. Governance & leadership**		**Vic**	
A.1 Leadership	A.1.1 Has childhood obesity prevention been identified as a priority by leadership (Premier/First Minister or Health Minister)?		A key priority in the *Victorian Public Health and Wellbeing Plan* is healthy eating and active living, the prevention of obesity is seen as a key benefit to improvements in these areas. A target in the *Victorian Public Health and Wellbeing Outcomes Framework* is a 5% decrease in prevalence of overweight and obesity in children by 2025.<br>The VicHealth-led obesity consensus statement *A Healthier Start for Victorians* (Link) focuses on the prevention of obesity in childhood, developed by the Healthy Eating and Active Living Roundtable (which represents many health and wellbeing NGOs, professional organisations, and university research groups). However, VicHealth are an independent health promotion organisation and their priorities do not represent those of the Victorian Government. In the executive summary the consensus statement notes: "There is an urgent need for obesity prevention to become a more prominent health priority" (p. 2), indicating consensus organisations believed it was not a government priority at the time. This mirrors a statement by the informant that each Victorian Public Health and Wellbeing Plan reflects "the priorities of the government of the day" (**Vic informant**), rather than a long term approach.
	A.1.2 **Key policy/policies**: Is there an overarching policy framework, or a series of key policies or action plans to guide initiatives for the early prevention of obesity in childhood?		The Victorian Health Plan is supported by the *Victorian Health Priorities Framework 2012-2022* (Metropolitan Health Plan (Link) and Rural and Regional Health Plan (Link), referencing that the VPHWP and MPHWPs are the key prevention and health promotion mechanisms in Victoria (cross-government and cross-sector initiative).<br>The Department of Health and Human Services (DHHS) develops Public Health and Wellbeing Plans (VPHWP) every four years. The *Victorian Public Health and Wellbeing Plan 2015-2019* (VPHWP) (Link) notes life course and place-based approaches, identifying both early years settings and the broader context of liveable neighbourhoods and addressing inequities. Healthier eating and active living are the first priority of the VPHWP, for the prevention of obesity and chronic disease but also for the other social benefits that come with being active. The strategic directions for this section focus on promoting diets consistent with the Australian Dietary Guidelines, providing a supportive food system, and a supportive built environment (for active transport and access to nature). Local councils are required to develop Municipal Public Health and Wellbeing Plans (MPHWP), and this focus on local implementation is the central feature of health promotion and prevention work in Victoria. This fits within the broader Victorian context of focusing on the local context, such as with the nine *Regional Partnerships* established by the Victorian Government in 2016, each of the nine partnerships have several local councils (Link).

			Early Childhood Reform (Link) is led by the Department of Education and Training (DET) and includes a $202.1 million investment to support a cohesive early childhood system. It runs across ECEC settings (including kindergartens), Maternal and Child Health services, and community settings (such as playgroups and supported playgroups). The *Supporting Children and Families in the Early Years: A Compact between the Department of Education and Training, Department of Health and Human Services and Local Government (represented by the Municipal Association of Victoria (MAV))* (The Early Years Compact) (Link) aims to ensure coordination between DHHS, DET and MAV across the early childhood system. It sets out that DHHS, DET, and local councils are all responsible for the health and wellbeing of children in the early years – and links the early childhood systems (ECEC settings, health and community settings) with broader place-based environments of communities.<br>*Koolin Balit: Victorian Government Strategic Directions for Aboriginal Health 2012-2022* (Link) seeks to increase the duration and quality of life for Aboriginal people in Victoria Key priority 1: A healthy start to life focuses on support throughout pregnancy and the first year of life (including breastfeeding and appropriate timing of introduction to solids) via antenatal care, linking up services (continuity of care). Followed by Key priority 2: Healthy childhood to support attendance at the state-wide universal child health program (KAS, see D.2.1) and support the Victorian Aboriginal Controlled Community Health Organisation (VACCHO)-led Victorian Aboriginal nutrition and physical activity strategy (see (A.3.1)
	A.1.3 Does the state legislation for public health include prevention/health and wellbeing?		The *Victorian Public Health and Wellbeing Plan* (VPHWP) is a statutory requirement under the *Public Health and Wellbeing Act 2008* (Link), required every four years. Prevention is a primary principle of the Act. The Act provides an authorising environment to engage across government and partner with both community and private sectors to protect and promote health and wellbeing. The Victorian model for health promotion and prevention efforts focus on local delivery, primarily through local government but also through state-wide and community NGOs. The VPHWP is required to take the determinants of health into account, as are the MPHWPs from local government.
	A.1.4 Are their statutory grant-giving bodies with a remit to fund prevention-related community projects?		The Victorian Health Promotion Foundation, i.e. *VicHealth* (Link) is a statutory health promotion agency with independent authority to provide funding and other supports to local councils, NGOs and community organisations for the purpose of health promotion, such as the *VicHealth Partnership Grants* (Link), *Local Government Partnership Grants* (Link).
A.2 Partnerships	A.2.1 Are partnerships across government noted in 'key policy' identified above?		Partnerships are more clearly defined between governments (i.e. between state and local governments) and with community NGOs rather than across government agencies of the state government, in line with Victoria's model of health promotion.
	A.2.2 Are there formal mechanisms for collaborative exchange across sectors (e.g. working groups,		The *Victorian Healthy Eating Enterprise* is a forum with the aim of improving healthy food environments and includes membership across state government as well as local government, public health professional and advocacy organisations and businesses (see

	policy/outcome joint statements, embedded health positions in agencies outside of health)?		B.1.3). VicHealth has extensive partnerships with local councils – these are in line with Victoria's model of local implementation. A key feature of the Vic health system is the outsourcing of much of the prevention policy implementation beyond the health department. The department relies on these organisations to feedback on community needs: "We have Vic Health, which is a statutory body. We've got Cancer Council… Nutrition Australia, Diabetes Vic, and then a whole bunch of community health services and councils. All of them would be engaging directly with the community, and we hear from them" (**Vic informant**).
A.3 Equity	A.3.1 Do the key policies identified outline the structural (incl. social/commercial) causes of obesity?<br>(such as employment/family income, affordable or social housing, adverse early childhood experiences, food security, food systems including promotion, built environment and access to safe/appropriate spaces for being active, etc)		The 2015-2019 VPHWP identifies protective factors to reduce the likelihood of chronic disease include antenatal and early childhood health and positive early experiences, access to quality health, education, and care services, as well as access to healthy food, and 'social capital' including strong family connections and community networks. It identifies the 'proximal' causes of ill health and notes that socioeconomic factors have an estimated 40% impact on health, "compared with health behaviours (30 per cent), clinical care (20 per cent) and the physical environment (10 per cent)" (p.19). Listed examples of the determinants of health include: "early childhood experiences, education, employment, income, social and economic status, housing and geography, social support networks, access and use of health services…" (p.19). It notes the impact of global trends in employment (more casual positions, less security) on family income and the impacts of global industrialised food production on securing sustainable agricultural production and food security.<br>The *Early Years Compact* and *Early Childhood Reform* (see A.1.2) identify the high risk for poor health outcomes as a result of adverse early childhood experiences.<br>The Aboriginal nutrition and physical activity strategy, *Closing the Nutrition & Physical Activity Gap in Victoria* (Link), prioritises nutritional health of Aboriginal mothers and babies, health policy in key settings, community-based interventions, and food security.
	- A.3.1.a Do recommendations for action/initiatives address these structural causes?		The 2015-2019 VPHWP examples of strategic directions include working across the entire food system, encourage active transport and neighbourhood design, improving mental health and address discrimination and stigma, encourage interaction with the natural environment. The settings for these actions to undertake place-based approaches, focused on healthy and sustainable environments.<br>VicHealth supports a range of initiatives to improve food security, e.g. the *Food for All* project (see B.2.1b).<br>The *Early Years Compact* (see A.1.2) in July 2018 identified three priorities: increasing participation in MCH services and kindergarten for Aboriginal families, increased participation in early years services of children known to child protection, and improve
	A.3.2 Are target populations (with higher risk of developing obesity) identified for additional support?		Target populations identified as having higher risk are locality (those living in rural areas), socioeconomic status (especially relating to household income) and Aboriginal people.

B. Environments in which we live (e.g. work, shop, eat, be active and play)		Vic	
B.1 Health supportive environments	B.1.1 Do planning policies orientate built environments towards principles of active living?		The Planning Act has not been updated since 1987. This represents an opportunity to either integrate health promotion into planning legislation or devolve local planning decisions (especially as they relate to health promotion and the authority already devolved to local governments under the *Public Health and Wellbeing Act*).<br>Some guidelines exist to support planning and built environment design, e.g. the *Urban design and health guidelines* (Link) and the *Office of Victorian Government Architect* (Link).
	B.1.2 Are there investments for public infrastructure (e.g. footpaths, bikeways, or greenspace) to encourage being active?		Significant investments are made for active transport across the state, see *Walking & cycling* (Link) (Transport for Victoria). The VicHealth *Physical Activity Strategy 2018-2023* (Link) enables grants and supportive services to local councils.
	B.1.3 Are there food/nutrition policies aimed at ensuring a nutritious, affordable, accessible food system? (e.g. incentivise local food production or increase healthy food access in disadvantaged communities, zoning policies, or incentives to retailers)		Local governments have an enabling policy environment to implement health promoting policies, including *Environments for Health: Municipal Public Health Planning Framework* (Link) (based on WHO Healthy Cities) and further authorised via the *Public Health and Wellbeing Act*. This could be enhanced further by devolving authority to local governments to make planning decisions relating to food, based on health and wellbeing, at this stage planning legislation has not been updated from some time.<br>The *Victorian Public Health and Wellbeing Plan 2015-2019* upholds healthy eating as one of six pillars of health promotion and chronic disease prevention.<br>*Healthy Choices* (Link) are a range of several setting-specific guidelines to improve food offerings across a range of health, sport and recreation, park and workplace settings, underpinned by the *Healthy Choices: Food and drink classification guide* (Link), they relate to both the provision and promotion of these foods and drinks in a range of settings (hospital and health services, sport and recreation centres, workplaces, parks). This work relates to the COAG Obesity Working Group activities.<br>The VicHealth *Healthy Eating Strategy 2017-19* (Link) had four focus areas including reducing salt consumption, swapping SSBs for water, increase fruit and vegetable intake and food environment policy. At the time of mapping attention was predominantly on the first two focus areas.<br>Additionally, the *Victorian Healthy Eating Enterprise* (Link) sought to improve access to healthy food, increase fruit and vegetable consumption and decrease consumption of SSBs through a forum including state and local government, health sector and health promotion organisations, advocacy groups and not-for-profits and partners with businesses, workplaces, etc to improve local food environments.
	B.1.4 Are there programs to support vendors to improve food offerings in food outlets (restaurants, cafes, take-away, vending machines)?		The *Victorian Healthy Eating Enterprise* (VHEE) (also see B.1.3) seeks to encourage a healthy eating culture across the state and works with local governments and businesses to improve food and drink offerings in line with the *Healthy Choices* policy. While this policy applies to arrange of government settings, the VHEE seeks to extend its principles more broadly across the state’s food businesses (i.e. beyond government-controlled settings).

			The *Healthy Eating Advisory Service* is available to support food outlets, food industry, caterers and suppliers (Link) (NGO: Nutrition Australia Vic Division).<br>The *Healthy Food Connect Framework* (Link) is a model to improve local food systems partnering with local government (needs assessment, prioritise actions, local food network, implement initiatives, embed healthy food access into MPHWPs). The Framework is supported by the *Healthy Food Charter* and was developed under Healthy Together Victoria.
	B.1.5 Is nutrition information at food outlets (menu board labelling) required by legislation?		The *Food Amendment (Kilojoule Labelling Scheme and Other Matters) Act, 2017*, or the *Kilojoule Labelling Scheme* (Link), requires menus and food tags to display kilojoule content of ready-to-eat food and drinks – it applies to large chain food businesses with 20 or more in Victoria or 50 or more outlets nationally, and additionally to large chain supermarkets (those same outlet numbers apply).
	B.1.6 Is there engagement with food retail (supermarkets, grocers, corner stores, etc) to reduce the availability and promotion of discretionary choices in-store?		Several elements come together at this policy area. Some of the *Victorian Healthy Eating Enterprise* work engages with food retail, the *Healthy Choices* framework requires food procurement meet healthy food provision standards (which provide stable income to business and act as a test case for interest in businesses selling healthier food options), and the *Eat Well @ IGA* (Link). This trial is a partnership between researchers (Deakin University), government (VicHealth and City of Greater Bendigo), and food retail (7 IGA supermarkets), with national funding from the NHMRC. Its purpose is to use health promotion messages in store and provide evidence of the efficacy of such interventions.
	B.1.7 Are local governments empowered to encourage health-supportive environments?		Under the *Environments for Health* framework and the *Public Health and Wellbeing Act 2008* (see Part 3, Division 3, e.g. requirement for *Municipal Public Health and Wellbeing Plans*) local governments are required to impact on health supportive environments (although some policy tools are not available to them, e.g. land use/ zoning tools under the Planning Act).
	B.1.8 Are there any initiatives to reduce exposure to the marketing/promotion of discretionary choices in:<br>- B.1.8a out-of-home advertising (billboards, transport vehicles, street furniture, transport hubs such as train stations) within government control?		Not at the time of mapping
	- B.1.8b healthcare settings?		*Healthy Choices* (see B.1.3) has *Policy guidelines for hospitals and health services* (Link), including health department offices. These guidelines apply to promotion and provision of foods and drinks in specific settings.
	- B.1.8c other government-controlled buildings/parks?		*Healthy Choices* (see B.1.3) has *Healthy eating policy and catering guide for workplaces* (Link) and *Policy guidelines for sport and recreation centres* (Link) and *Policy guidelines for parks* (Link). These guidelines apply to promotion and provision of foods and drinks in specific settings.
	B.1.9 Are there policies limiting the availability/provision of discretionary choices in:<br>- B.1.9a healthcare settings (for visitors and staff)?		See B.1.8b

			(Update 2021, policy updated and extended to all 'in-house managed retail outlets, all vending machines and all catering within public health services' and the complete removal of all RED category drinks)
	- B.1.9b buildings, community centres, and parks under government control?		See B.1.8c
B.2 Health promotion campaigns	B.2.1 Are there health promotion campaigns (any media type) aimed at:<br>- B.2.1a encouraging healthy lifestyle behaviours?		At the time of mapping the main healthy lifestyle media campaigns were directed towards reducing SSB consumption through the promotion of water in the Water Initiative, including *The H3O Challenge* social marketing campaign and grants to local councils (18 participated) to promote a 30-day challenge to replace SSBs with water (Link).<br>Previously the *Healthy Together Victoria* program had state-wide and local health promotion campaigns aimed at healthy lifestyles of families (Link).
	- B.2.1b developing/supporting healthy food systems and built environments (incl. community-capacity building)?		A range of healthy eating support services exist to support communities, e.g. food outlets (see B.1.4) and local governments under the Public Health and Wellbeing Act, including VHEE, VicHealth, HEAS, etc.<br>VicHealth has multiple programs focused on improving access to fruit and vegetables and encouraging healthy food cultures. Some examples include *Food for All* (2005-2010) supported local councils to improve elements relating to food security (transport, housing, land use and economic development) (Link). Programs such as *3000 Acres* and the *Open Food Network* (Link) were new approaches to improve local fruit and vegetable supply through innovation grants (Healthy Eating (seed) Innovation Challenge). The *Healthy Eating Strategy* is updated every two years (e.g. 2017-2019 Link) and contributes to the evidence base for what works and what has public support, e.g. supporting businesses to have healthier food offerings by demonstrating public support or developing programs to trial healthier stocking and promotion practices in food retail/supermarket environments.<br>(previous) *Healthy Together Victoria* (Link) was delivered across 14 local government areas, based on "a long history of community-based obesity prevention initiatives" (**Vic informant**), such as *Collaboration of Community-based Obesity Prevention Sites* (CO-OPS), *Romp & Chomp*, *It's Your Move,* and *Fun & Healthy in Moreland*. This program funded staff in community health and local government to deliver community-specific interventions. *Healthy Together Victoria* has ceased, "but some of those local communities have continued to work on these issues and you know, continued to draw them into current policy environments." (**Vic informant**).
**C. Early childhood education and care (ECEC) settings**		**Vic**	
C.1 ECEC settings	C.1.1 Are there support programs for centre-based care settings to:<br>- C.1.1a encourage healthy food provision? (e.g. management: policies and menu audits; staff: training and resources; families: resources)		Two services are available state-wide to ECEC services – both are supplied by NGOs, funded by the DHHS. Ongoing support is provided through the *Achievement Program* (Link), which was developed in partnership with DHHS, the Cancer Council (an NGO), and the Department of Education and Training to "add further depth to the National Quality Standards, and in fact it's really marketed to early childhood services that if you want to meet these standards you can get help through joining the Achievement

			Program, and also using the Healthy Eating Advisory Service” (**Vic informant**). DHHS worked with both the schools and early childhood parts of the Education department and has developed a strong partnership over time.<br>Participation in the *Achievement Program* at the time of mapping was about 38% (of >1100 services) of early childhood services in Victoria. A concurrent program called the *Healthy Eating Advisory Service* (Link), run by Nutrition Australia Victorian Division (an NGO), “works hand in hand” with the *Achievement Program* (**Vic informant**). This service reviews ECEC service menus using an online tool called *FoodChecker* (Link).<br>These statewide services are supported by two policies: *Menu Planning guidelines for long day care* (DHHS, Nutrition Australia) and *Healthy Eating in the National Quality Standard: A guide for early childhood education and care services* (Department of Education and Training, Nutrition Australia, DHHS).<br>(Update 2021, Victoria will fund kindergarten programs for children from 3 years of age (the roll out is staged across the state, starting in areas of higher disadvantage first), and because so many children attend kindergarten through long day care services, it is an additional point of leverage to encourage services to improve food offering and embed health literacy and positive relationships with healthy lifestyle behaviours into the curriculum (Link))<br>The *feedAustralia* initiative is not actively promoted in Victoria although ECEC services are eligible to join and receive their support (Link).
	- C.1.1b provide food and physical activity experiences as part of the curriculum?		The *Achievement Program* supports curriculum (see C.1.1a)
**D. Health (community and tertiary health settings and health promotion activities)**		**Vic**	
D.1 Antenatal and birth services	D.1.1 Does antenatal care screen and manage hypertension, hyperglycaemia, appropriate gestational weight gain?		*Clinical Practice Guidelines: Pregnancy Care 2019 edition* (national guidelines) recommend monitoring of blood pressure, weight and screening for hyperglycaemia (Link). Victoria has separate guidelines for *Gestational diabetes* (Link) and *Hypertension in pregnancy* (Link) and *Obesity during pregnancy, birth and postpartum* (Link).<br>Three main modes of maternity/antenatal care: midwifery services (hospital
	D.1.2 Antenatal care within public health services:		Antenatal services are offered at public hospital midwife clinics and community-based centres, or through GP-shared care (or privately).
	- D.1.2a Do they include nutrition counselling for healthy pregnancy or are there other healthy lifestyle support programs available during pregnancy?		There is limited information available on the services offered during antenatal care in terms of education/ healthy lifestyle support for the client.<br>Some targeted, state-wide programs exist. The *Healthy Mothers, Healthy Babies* program (Link) supports at risk women during their pregnancy and across the transition of care from antenatal services (state) to postnatal MCH services (local). The program is the outer suburbs of the greater Melbourne region and in some regional areas (in areas of higher disadvantage). In this program healthy lifestyle education (including nutrition and being active) are included based on client need.

			Koori maternity services (Link) are an Aboriginal maternity service available at 14 sites across Victoria.<br>The *Better Health Channel* has some online *Healthy pregnancy* resources (Link)
	- D.1.2b Is breastfeeding education free (separately or embedded into antenatal education/services)?		The *Breastfeeding guidelines* recommend breastfeeding education should happen throughout antenatal education (Link).<br>*Healthy Mothers, Healthy Babies* program is a targeted program (not universal) that is offered to pregnant women at risk. It includes breastfeeding education.
	D.1.3 Do maternity facilities fully adhere to the Baby Friendly Health Initiative (BFHI) (based on *Ten Steps to Successful Breastfeeding*)?		Only eight public hospitals are BFHI-accredited.
D.2 Early childhood health services	D.2.1 Are there free health/parenting services to support early childhood growth/nutrition (e.g. breastfeeding, complementary feeding, transition to family foods)?		Overarching policy guidance for early years support sits under the *Maternal and Child Health Service framework* (Link).<br>The *Key Ages and Stages* program (Link) is a universal child health check offered across ten appointments, located at Maternal and Child Health (MCH) Services. After the initial home visit, the following nine sessions are held at 2 weeks, 4 weeks, 8 weeks, 4 months, 8 months, 1 year, 18 months, 2 years, 3 ½ years. In addition to the health check-up, the program focuses on health promotion and has an extensive policy framework including practice guidance. The program is well attended across the state.<br>Additionally, MCH services have drop-in clinics, and appointments can be made for additional support. *Enhanced MCH programs* are available for families needing additional support. *First time parent groups* (Link) are free group-based programs and also run by MCH nurses.<br>There are ten Aboriginal MCH services (Link) in Victoria, providing better health outcomes and ensuring access to the universal child health check program for Aboriginal and Torres Strait Islander families.
	- D.2.1a Is information to support parents readily available (e.g. phonelines, websites)?		The *Better Health Channel* website has some online *Child health (0-6)* resources (Link)<br>*Maternal and Child Health Line* is available 24/7 and offers support on nutrition, breastfeeding, child and family health and parenting (Link)
	- D.2.1b Do these include breastfeeding support?		Yes
	D.2.2 Are there healthy lifestyle (education) programs to support families during early childhood?		The *INFANT* program (Link) was developed in a research setting in 2007. The group-based program ran for 90 minutes across six sessions (when children are 3, 6, 9, 12, 15 and 18 months), delivered by MCH nurses, dietitians and health promotion officers. In 2011, with DHHS funding, a small-scale translation project was funded (under NPAPH), led to the delivery of the program in 8 of the 12 local government areas invited to run the program. Some local councils continued to run the program when the DHHS funding ended. While DHHS did not fund this program at the time of mapping, they supported Deakin University to apply for federal funding through a NHMRC Partnership Project to upscale the program/ make it available across all Victorian local governments, fitting into Victoria's local delivery model. (Update 2021, this grant application was successful, sessions were reduced to four at 3, 6, 9, and 12 months with an app to support extended content delivery).

	- D.2.2a Are target populations identified and actively recruited for programs?		The initial 12 local councils in the small-scale translation of the INFANT program, from a research to real world setting, were identified as being in areas of higher disadvantage
	D.2.3 Are Supported Playgroups offered for families that need additional support and do they include healthy lifestyle skills?		*Supported Playgroups* (Link) are targeted programs available state-wide from department of families. Focused on supporting parenting skills, attachment, and child development, they do include healthy lifestyle elements such as family mealtimes. Families can access these services through their local council or via the MCH nurse.
D.3 Workforce	D.3.1 Are there training and resources available for health care professionals to support families?		None found at the time of mapping.
	- D.3.1a Is preconception advice for nutrition and being active provided to prospective parents?		None found at the time of mapping.
	D.3.2 Is there a state health promotion…<br>- D.3.2a …agency (independent or adjunct to health department)?		*VicHealth* (Link) is an independent, statutory, health promotion agency, established under the Tobacco Act of 1987. One of its core functions is to fund health promotion activities with a diverse range of partners.<br><br>*Population monitoring*: regular CATI survey, similar to NSW with additional questions on social support/connectedness - adults only (*Victorian Public Health Survey* Link). Additionally, the *Public Health and Wellbeing Outcomes Framework* (Link) is comprehensive across a range of policy domains and has substantial potential to contribute to evidence-informed policy in Victoria and for other jurisdictions. The *Victorian Child Health and Wellbeing Survey* – parents of children from birth to 12 years (Department of Education) covers health in pregnancy, child health, child growth, family functioning, nutrition, etc (Link).
	- D.3.2b …workforce (to implement initiatives locally)?		While there are health promotion staff within community health settings, no information on centralised support mechanisms within the DHHS was found at the time of mapping. As Victoria does not have local hospital networks (rather it has 86 distinct entities across the state) this seems to further support the finding that health promotion through community health settings does not have a centralised support mechanism.<br>Local governments are required to develop RPHWPs in response to the VPHWP. Under the Public Health and Wellbeing Act, the primary health promotion workforce in Victoria are situated with local governments. Additional health promotion workforce exists within the DHHS and their partners (e.g. via VHEE) and VicHealth. *VicHealth* provides a range of grants to local governments and local NGOs across a range of health promotion areas. Previously, under *Healthy Together Victoria* (during the NPAPH) the DHHS funded the health promotion staff within the participating local councils. It is unclear what capacity local governments have to provide ongoing capacity building to the health promotion workforce, or if there is any state government oversight.

2.8 Western Australia

Area	Guiding questions	Result	Notes
**A. Governance & leadership**		**WA**	
A.1 Leadership	A.1.1 Has childhood obesity prevention been identified as a priority by leadership (e.g. Premier or Health Minister)?		The Sustainable Health Review – Interim report was released in early 2018. Its first recommendation was "The Department of Health should take and active leadership role across the public sector in developing whole-of-government targets with potential impact for better health outcomes, commencing with childhood obesity" (p.25, Link). These recommendations were endorsed by the WA Government.<br>The *WA Preventive Health Summit* in March 2018 (Link) focused on actions on obesity and alcohol. "That summit brought together key government agencies, NGOs, peak bodies and opinion leaders to talk about policy measures that government needs to consider in addressing both obesity and alcohol, two major public health issues for WA, though certainly not the only public health issues. Ideas explored in the Summit have enabled some policy work to be considered that perhaps wasn't necessarily on the government agenda… Well some of the options that were put forward included… the introduction of kilojoule labelling on fast food menus, strengthening of compliance with a more rigorous healthy food and drink policy within the WA health system, and a consideration to whether a healthy food and drink policy is something that could be adopted across public sector agencies as a whole" (**WA informant**).<br>At the time of mapping the *WA Healthy Weight Action Plan (2019-2024)* was being written. More than 1000 stakeholders contributed to the process, called the WA Obesity Collective Project. The approach focused on the collective responsibility for addressing obesity.<br>The informant noted that this was a change in direction from the previous party in leadership, that as an issue obesity prevention has "…been driven by the extent to which prevention has figured as a core policy priority for individual political parties… the new [WA] government, and particularly the new health minister has a very strong commitment to prevention" (**WA informant**).
	A.1.2 Key policy/policies: Is there an overarching policy framework, or a series of key policies or action plans to guide initiatives for the early prevention of obesity in childhood?		The *Sustainable Health Review* was undertaken by a group of experts appointed by Government who offered 8 strategies including 30 recommendations (Link). The final report identified four areas for sustainability: obesity, early childhood, family safety, and homelessness. Key recommendations relevant for the early prevention of obesity include:<br>Strategy 1 commitment and collaboration to address public health issues.<br>• Recommendations: Lotterywest/Healthway provide funding to local government and community organisations for nutrition and physical activity; develop an obesity prevention action plan; ban unhealthy food promotion and implement healthy food promotion policies in all state agencies and change planning laws to limit sale of unhealthy food and drinks, and increase access to healthy food (B.1.3); target health needs relating to social determinants including housing, disability support and child and family safety<br>Strategy 3 focus on both the start of life and the end of life<br>• Recommendations: WA Health should actively partner in the Early Years Initiative, Supporting Communities Forum, and Early Years Network; consider things like co-location of services

			with Health, Department of Education, Department of Communities; targets for prenatal health and breastfeeding; statewide program for culturally safe pregnancy, birth and postnatal care for Aboriginal families<br>Strategy 7 culture and workforce<br>• Recommendations: partnerships for cross-sector collaboration, policy, and research; active participation linking up between community organisations and across public sector agencies<br>The *Sustainable Health Review* influenced a range of policies. The *State Public Health Plan 2019-2024* (Link) (SPHP) endorses the *Sustainable Health Review*, and the implementation of the *WA Health Promotion Strategic Framework 2017-2021* (Link). The SPHP supports families, settings and environments to improve healthy eating and active living, at first to stop increases in obesity prevalence, then aim to decrease prevalence in the long term. The *Public Health Planning Guide for Local Government* (Link) provides support information for local councils to develop LPHPs (also relates to B.1.7). The principles of the *WA Health Promotion Strategic Framework* include a whole-of population approach, cross-government partnership and coordination, a life course approach, equity, and inclusivity. It provides more detailed actions for the priorities set out in the SPHP.<br>The Education and Health Standing Committee of WA Legislative Assembly tabled a report on the role of diet in type 2 diabetes prevention and management, *The Food Fix* (Link). Some of the recommendations include: 9. Restrictions on food marketing in settings government control; 10. Implement menu labelling regulation; 11. Extend *Healthy Options WA* (see B.1.9a) to all government-funded settings; 12. Amend the planning Act to allow consideration of health and wellbeing in fast food planning applications; 13. Undertake nudging strategies to shift consumer grocery shopping behaviour in food retail.<br>*Connecting Early Years Networks* (Link) (Department of Communities) is a platform to link up and support collaborative practices between a range of services focused on the early years and parent support: education, health, local government, community services in any given area. The (targeted) *Early Years Initiative* (Link) supports families of children 0-4 years. It is a partnership between the Departments of Communities, Education, and Health; CoLab (Collaborate for Kids, Telethon Kids Institute)), the Minderoo Foundation and several partner communities in metro, remote, and very remote areas. It aligns with areas of vulnerability identified by the Australian Early Development Census (including health and wellbeing). The learnings from these partnerships could provide evidence for ways to support families experiencing vulnerability and scaling up services statewide.
	A.1.3 Does the state legislation for public health include prevention/health and wellbeing?		The *Public Health Act 2016* (Link) defines public health as "the wider health and wellbeing of the community and the combination of safeguards, policies and programs designed to protect, maintain, promote and improve the health of individuals and their communities and to prevent and reduce the incidence of illness and disability" (SPHP, p.2). The WA participant noted that the Act, "although it was passed in 2016, has been a 20-year journey… The new legislation takes in preventive health, for instance, and includes general principles such as the precautionary principle. So it replaced an out-dated Act that hadn't anticipated innovation by industry, for instance, or how the health system would need to be able to address this" (**WA informant**).

			Implementation of the Act was a staged process (see timeline Link). Principles include: sustainability, precautionary, proportionality, intergenerational equity, respect of local government authority in public health matters. The Act does not contain language on partnerships across sectors.<br>The first four stages were mostly technical to enable later stages of the Act, e.g. requiring local governments to report on their performance. The Act requires changes to public health planning, and a State Public Health Plan (SPHP) to be developed every five years, the first full SPHP is the *State Public Health Plan for Western Australia 2019-2024* (Link). The fifth and final stage of the Act implementation will be the fulfilment of public health planning at the local level, requiring local councils to develop Local Public Health Plans (LPHP) – which will come into effect in July 2022. Some local councils have already commenced with LPHPs. The Act complements the integrated planning process (Link) and section 5.56 of Local Government Act 1995 (Link).<br>Additionally, the *Health Services Act 2016* (Link) was an update that provides governance, accountability, and control over allocation of resources across WA Health: the Health Department and its Health Service Providers (North/South/East Metropolitan Health Services (NMHS/SMHS/EMHS), The Child and Adolescent Health Service (CAHS), and WA Country Health Service (WACHS).
	A.1.4 Are their statutory grant-giving bodies with a remit to fund prevention-related community projects?		The *Western Australian Health Promotion Foundation Act 2016* (Healthway Act) (Link) merged two grant-giving bodies, Healthway (~$20 million spend) and Lotterywest (~$260 million community grant spend), and potential to leverage off the bigger buying power of Lotterywest. "The view was that the two organizations merging together provided some efficiency in terms of some shared corporate governance system. But also had potential to expand the reach and influence of Healthway and its messages" (**WA informant**).<br>The goal of Healthway was to build a healthy WA through funding community activities, sports, arts, health promotion projects and research. Priorities are outlined in the *Active Healthy People 2018-2023: Strategic Plan* (Link) identify funding availability for improve food security, food environments, built environments for physical activity as well as programs to improve food/nutrition literacy and being active. The WA study participant noted "There's potential to link some of the health messages to general Lotterywest grants and sponsorships as well. So there's a potential I suppose to spread some of the health promoting policies that would probably be limited to those programs, and organizations that are receiving funding from Healthway. And at the same time to amplify some of the health messages that Healthway supports. For instance, the Healthy Food and Drink policies, the Minimum Health Policy Requirements relating to issues such as [healthy food provision] that were always a condition of Healthway grants and sponsorships are now something that can be incorporated within Lotterywest's grants and sponsorships" (**WA informant**)<br>WA Healthway position on nutrition (Link) has a working definition of junk food to guide policies, to "ensure all organisations receiving $20,000 or more from Healthway develop and implement policies on healthy food choices" (p.3).<br>Department of Communities also has the *Community grants program* (Link).
A.2 Partnerships	A.2.1 Are partnerships across government noted in 'key policy/policies' identified above?		Yes, noted in the *Sustainable Health Review*, the SPHP, and the *Public Health Act 2016* – e.g. "The health and wellbeing of a community is a shared responsibility, and not the sole responsibility of a single agency" (SPHP, p.6)

	A.2.2 Are there formal mechanisms for collaborative exchange across sectors (e.g. working groups, policy/outcome joint statements, embedded health positions in agencies outside of health)?		The *Sustainable Health Review* notes utilising two cross-government mechanisms to progress priorities including obesity, early childhood, family safety and homelessness.<br>The *Supporting Communities Forum* brings together public sector and community services leaders to support the Supporting Communities Policy (Link), overseen by the Community Safety and Family Support cabinet sub-committee. The *Director General Implementation Group* both directs and implements cross-government social policies.<br>Noting the use of HiAP in SA, the informant felt the methodologies were over burdensome, but still saw some merit in elements of the approach: "I'm not a great fan of Health in All Policies in its pure sense… often it seems to require a fairly weighty, complex bureaucratic governance framework... I would probably call [our processes] Health in All Policies by stealth…" (**WA informant**).
A.3 Equity	A.3.1 Do the key policies identified outline the structural (incl. social/commercial) causes of obesity?<br>(such as employment/family income, affordable or social housing, adverse early childhood experiences, food security, food systems including promotion, built environment and access to safe/appropriate spaces for being active, etc)		SPHP: The Chief Health Officer selected the objectives for the SPHP by considering a range of elements including the "ability to influence the determinants of health in some way" (p.2) and identifies public health as: nutritious food, walking and cycling infrastructure, recreational, sports, and green spaces, safe housing, built environment design, etc…<br>The high cost of food in WA is noted, with statewide food insecurity at 3.5%, and additionally for Aboriginal people aged over 15 years was 27% (p.15).<br>*Homelessness in Western Australia* (Department of Communities Link) identified the strong links between insufficient income to meet basic needs let alone undertake preventive steps for health and wellbeing. "Without sufficient resources it is incredibly difficult to take the necessary proactive, preventative steps with regard to issues facing those experiencing homelessness, let alone to gain secure employment of filling gaps" (p. xiii)
	- A.3.1.a Do recommendations for action/initiatives address these structural causes?		Policy priorities under objective to empowering people to live healthy lives include healthy eating (foster healthy food environments, increase availability and affordability of nutritious food), a more active WA (promote environments for physical activity, reduce barriers to being active across the life course), and stop rise in obesity (promote health supportive environments) – in addition to motivation and skill/knowledge-based activities aimed at individual behaviour change.<br>Strategic directions identified in the *WA Health Promotion Strategic Framework 2017-2021* identify healthy policy, legislation/regulation, economic interventions, etc, for health supportive environments.
	A.3.2 Are target populations (with higher risk of developing obesity) identified for additional support?		Aboriginal people, people living in low socioeconomic circumstances and/or rural and remote areas, with mental illness, with disability, carers and family of people with sickness and disability, some culturally and linguistically diverse populations.
**B. Environments in which we live (e.g. work, shop, eat, be active and play)**		**WA**	
B.1 Health supportive environments	B.1.1 Do planning policies orientate built environments towards principles of active living?		At the time of mapping public health did not feature in the Planning Act. However, in anticipation of Stage 5 of the *Public Health Act* (i.e. the requirement for LPHPs) commencing by mid-2022, preparatory work to consider new regulations for the built environment had commenced at the time of mapping, including submissions by WA Health for the consideration of healthy eating and active living in planning legislation.<br>The state planning framework (Department of Planning, Lands and Heritage) has several relevant elements. The overarching long-term plan, the *State Planning Strategy 2050* (Link) identifies several strategic directions: economic development (sustainability of agricultural land, ensure local food supply

			chains) physical infrastructure (movement of people through connected networks) social infrastructure (housing, public spaces, and health and wellbeing) and protecting the environment. *Design WA* (Link) was an initiative to ensure good design in planning and development. *Liveable neighbourhoods* (Link) sits under *Design WA*. It is an planning operational guide that started in 2009 from the Western Australian Planning Commission (WAPC) and revised in 2015. It has six elements (community design, movement network, activity centres, lot design, public open space, education).<br>Health statement/information for use by state and local governments and developers, *Evidence supporting the creation of environments that encourage healthy active living* (Link) (2014) this work was linked to the Heart Foundation (NGO) *Healthy Active by Design* work (see Link)
	B.1.2 Are there investments for public infrastructure (e.g. footpaths, bikeways, or greenspace) to encourage being active?		The Department of Transport has the *Active Transport* strategy (Link) and the *Your Move* (Link) program to encourage active transport and aims to reduce congestion and pollution while improving community health and wellbeing.<br>The Department of Local Government, Sport and Cultural Industries has the *Active Living for All 2017-2019* strategy (Link) takes a life course approach (recognising the early years) and key settings such as ECEC, schools and workplaces and 'active places' in the built environment (i.e. through planning and design). The strategy links to work on liveable neighbourhoods, the state planning strategy, the health promotion framework, and Early Years Plans.<br>Healthway's strategic plan *Active Healthy People: 2018-2023* (Link) has five priorities (healthy eating, physical activity, mental health, preventing alcohol harm and creating a smoke-free WA) which include funding projects relating to shaping environments to support good mental health and enable physical activity.<br>In 2019, $146 million of state budget was allocated to cycle infrastructure investment for local councils and alongside major transport infrastructure upgrades (media statement, Link), and Department of Transport Perth/Regional Bicycle Network Grants Program ($3.58/2.9 million over two years). The overarching strategy is the *Western Australian Bicycle Network Plan (2014-2031)* (Link), with oversight from the cycling team within the Department of Transport.<br>Public open space is included in the draft 2015 Liveable Neighbourhoods (see B.1.1)
	B.1.3 Are there food/nutrition policies aimed at ensuring a nutritious, affordable, accessible food system? (e.g. incentivise local food production or increase healthy food access in disadvantaged communities, zoning policies, or incentives to retailers)		The cost of healthy food in WA was recognised by the health department as unaffordable for many WA families (e.g. 2013 Healthy Food Basket was $581.27, in 2014 66,180 people reported food insecurity, see Link). In 2017 Lotterywest funded the Western Australian Council of Social Service to lead the *Food Relief Framework Project* (final report Link) which included the development of the *Food Stress Index* to support policy action at the local government level in a range of scenarios. In this report, the City of Mandurah was cited as having strong governance for their local network meeting.
	B.1.4 Are there programs to support vendors to improve food offerings in food outlets (restaurants, cafes, take-away, vending machines)?		*Healthy Menu Options* was a program that ran in some local councils (e.g. City of Armadale, Link) that supported food outlets to improve their food offerings. Menu items and stores that in general promote healthy foods are able to place the logo on their menu. The initiative was supported with collateral explaining the program to the public.
	B.1.5 Is nutrition information at food outlets (menu board labelling) required by legislation?		Was under consideration at the time of mapping

	B.1.6 Is there engagement with food retail (supermarkets, grocers, corner stores, etc) to reduce the availability and promotion of discretionary choices in-store?		None found at the time of mapping.
	B.1.7 Are local governments empowered to encourage health-supportive environments?		Under the *Public Health Act* (part 5) local councils will be required to develop *Local Public Health Plans* in line with State Public Health Plans (see A.1.2), although the implementation of stage 5 of the Act is not required until mid-2022, many local councils commenced the development of health and wellbeing plans from 2014 onwards (see Examples of WA local public health plans, Link). Supporting guidelines exist for local councils: *Public Health Planning Guide for Local Government* (2018) (Link), *Pathway to improving food security: A guide for local government* (2014) (Link), *Pathway to increasing active living: A guide for local government* (2015) (Link). In anticipation of Stage 5 commencing, preparatory work to consider new regulations for the build environment had commenced at the time of mapping.<br>Local councils could potentially use their LPHPs to justify a range of interventions such as restricting out-of-home advertising in settings within their control. However, to do so they would probably need significant capacity building support (see also D.3.2b). Another example, local governments are the enforcement agencies for the *Food Act 2008 (WA)* – this arm of the councils are in touch with food outlets often and could be upskilled to deliver a range of programs with food outlets and food retail to improve food and drink offering.<br>(Update 2021, City of Mandurah prohibited advertising of unhealthy food, tobacco, alcohol on all City managed lands and road reserves – effectively all street furniture. *Advertising in Road Reserves Council Policy*, see p.202 Link).<br>Finding 28 of *The Food Fix* (see A.1.2) : "The Public Health Act 2016 is a major public health reform that will require local governments to understand the health priorities of their communities and put in place programs to respond to them. However, resources for local governments to implement the reforms are lacking, particularly for those that are smaller" (p.111)
	B.1.8 Are there any initiatives to reduce exposure to the marketing/promotion of discretionary choices in:<br>- B.1.8a out-of-home advertising (billboards, transport vehicles, street furniture, transport hubs such as train stations) within government control?		In 2018 the WA Preventive Health Summit was held. It's focus was action on obesity and alcohol (Link), where a decision was made to ban advertising of alcohol on all public transport vehicles in order to 'limit young people's exposure to alcohol promotions via legislation'. This policy will take a phased in approach between the Public Transport Authority and the advertising company holding the two contracts for buses (for 2019) and trains (2022) and their related infrastructure. The media statement (Link) noted that advertising contributes a revenue stream of $8 million for the Public Transport Authority and the provision of transport services (with alcohol advertising comprising about 2% of revenue). Noting this may signal a greater barrier in the potential future removal of junk food advertising across OoH advertising spaces.
	- B.1.8b healthcare settings?		*Healthy Options WA Food and Nutrition Policy* (see B.1.9) requires that unhealthy food and beverages are not promoted.
	- B.1.8c other government-controlled buildings/parks?		No policies for government department/agency buildings at the time of mapping.<br>Canteens in sporting and leisure centres were supported through the *Fuel to Go Program* (Link). It is a requirement for any agency receiving funds from Healthway to adhere to the *WA Health Sponsorship*

			Policy (Link), mandatory policy for any entity receiving funds from WA Health cannot sponsorship related marketing practices into community organisations receiving government funding.
	B.1.9 Are there policies limiting the availability/provision of discretionary choices in:<br>- B.1.9a healthcare settings (for visitors and staff)?		*Healthy Options WA: Food and Nutrition Policy for WA Health Services and Facilities* (Link) is a mandatory requirement at all health services and facilities in WA. It is supported by the *How to Classify Food and Drinks Guide* (Link) which uses a traffic light system, and a range of resources for all retail outlets that operate in these settings.
	- B.1.9b buildings, community centres, and parks under government control?		None found at the time of mapping
B.2 Health promotion campaigns	B.2.1 Are there health promotion campaigns (any media type) aimed at encouraging healthy lifestyle behaviours?		WA health promotion focuses more on adults than children, e.g. *LiveLighter* campaign (obesity prevention campaign, delivered by Cancer Council WA) – online programs also available. *Your Move* (Link) is a program from the Department of Transport to encourage active transport
	B.2.2 Are there health promotion campaigns aimed at developing/supporting healthy food systems and built environments (incl. community-capacity building)?		No specific statewide programs found at the time of mapping. *Healthier Workplace WA* (supports workplaces to encourage healthy lifestyle behaviours) – approach is to impact on parents to then influence children (Link)
**C. Early childhood education and care (ECEC) settings**		**WA**	
C.1 ECEC settings	C.1.1 Are there support programs for centre-based care settings to encourage healthy food provision? (e.g. management: policies and menu audits; staff: training and resources; families: resources)		Not at the time of mapping.<br>A previous program existed in the early 2000s, *Start Right-Eat Right* was an award scheme to encourage childcare services to provide healthy meals. A study (Link) showed 80% of services that signed up improved their menus and at 2 years there was a 40% reach of centres. It used the SA resources (see SF2.5 area C.1)<br>Health funds an NGO to support the Healthy Food and Drink Policy in WA public schools. The NGO (WA School Canteens Association) only works in the school space so would not have the capacity to support the ECEC sector. In schools, principals are required to develop food provision policies for the whole-of-school. A similar policy requirement could be applied in WA.<br>Healthy food policies in school settings exist in most Australian jurisdictions, although such requirements have not been extended to the ECEC sector. The ECEC sector is regulated nationally under the National Quality Framework (NQF) (those regulations are implemented and monitored at a state level through the department of communities), so it would make sense to develop these standards in a nationally consistent way. The *feedAustralia* initiative offers support to ECEC sector via an online menu planning tool menu reviews (Link) – those jurisdictions which do not already provide such services could encourage services to access this program.
	C.1.2 Are there programs to support provision of food and physical activity experiences as part of the curriculum?		Not at the time of mapping

**D. Health (community and tertiary health settings and health promotion activities)**		**WA**	
D.1 Antenatal and birth services	D.1.1 Does antenatal care screen and manage hypertension, hyperglycaemia, appropriate gestational weight gain?		*Clinical Practice Guidelines: Pregnancy Care 2019 edition* (national guidelines) recommend monitoring of blood pressure, weight and screening for hyperglycaemia (Link).<br>The *Antenatal Care Schedule* is given to pregnant women from their chosen hospital (e.g. Link) along with their *Pregnancy Health Record* that they are to bring to all appointments. Scheduled health check-ups monitor pregnancy weight gain, hypertension and risk of gestational diabetes.
	D.1.2 Antenatal care within public health services:		Mothers who choose antenatal hospital care (10 hospitals and birth centres in the Perth-area (under North, South, and East Metropolitan Health Services) and 19 country hospitals (7 regions, all under the WA Country Health Service)) or midwifery care (under the *Community Midwifery Program* (Link) for women in Perth only) are seen solely through the state provided healthcare system. Other options include GP-shared care (includes all antenatal appointments) and private health care options.
	- D.1.2a Do they include nutrition counselling for healthy pregnancy or are there other healthy lifestyle support programs available during pregnancy?		Under *Antenatal Care Schedule*, recommendations are made for supplementation.<br>It was not clear at the time of mapping if all hospital and community care settings offer antenatal classes which include healthy lifestyle information during pregnancy, but some settings have pregnancy-care booklets (e.g. *Pregnancy, Birth & Baby* (Link) from King George Memorial Hospital/ North Metropolitan Health Service) which include a healthy pregnancy discussion at around 12-20 weeks in addition to antenatal classes. It is not clear how/if antenatal classes are offered to women living outside the metropolitan area.
	- D.1.2b Is breastfeeding education free (separately or embedded into antenatal education/services)?		Antenatal classes and mother's groups are offered at public hospitals/birth centres for mother's who choose antenatal hospital care and at community centres for those who choose midwifery care. Introduction to breastfeeding is included in these sessions and there are additional breastfeeding workshops offered (although these are women-only environments). It is not clear how/if antenatal classes are offered to women living outside the metropolitan area.
	D.1.3 Do maternity facilities fully adhere to the Baby Friendly Health Initiative (based on *Ten Steps to Successful Breastfeeding*)?		Three (out of 29) public hospitals and birth centres in WA have BFHI accreditation. The North Metropolitan Health Service has a range of educational tools available, Baby Friendly Health initiative Education Tools (Link).
D.2 Early childhood health services	D.2.1 Are there free health/parenting services to support early childhood growth/nutrition (e.g. breastfeeding, complementary feeding, transition to family foods)?		The *Community Child Health Program* (Link) offers health check ups at key developmental stages (noted in the child health record or 'purple book' appointments) at the *WA Child Health Centres* (Link). The Child Health Centres also offer drop in sessions and group sessions on early parenting, introduction of solids, sleep.
	- D.2.1a Is information to support parents readily available (e.g. phonelines, websites)?		The *Ngala Parenting Line* (Link) is co-funded by the Department of Local Government and Communities and the Child and Adolescent Health Service (external provider). The service is available 8am-8pm daily for parents of children aged 0 -18 years. The focus of this helpline is for parenting and child development concerns, but also to connect families to other services as they need them. The *HealthyWA* website is a consumer website, it has a section on *Parenting* and healthy lifestyle activities for children (Link)
	- D.2.1b Do these include breastfeeding support?		*HealthyWA* website has a section on *Breastfeeding* (under Parenting) and healthy lifestyle activities for children (Link) and provides links for additional support (the *Ngala Parenting Line*, and the metropolitan *Breastfeeding Centre of WA* (Link))

	D.2.2 Are there healthy lifestyle (education) programs to support families during early childhood?		The *Toddler Better Health Program* (Link) supported families of children aged 2-4 years, includes 10 weekly 90 minute face-to-face sessions, delivered by the Better Health Company. It was an extension of an existing program for children aged 7 to 13 years, due to roll out late 2018/ early 2019 (therefore not yet available as a statewide service at the time of mapping). WA was the only jurisdiction with a state-wide toddler-focused i.e. 2-4 years program. (Update 2021, the service was not being offered by the Better Health Company, it is unclear if this program has continued)
	- D.2.2a Are target populations identified and actively recruited for programs?		Nothing specific found at the time of mapping
	D.2.3 Are Supported Playgroups offered for families that need additional support and do they include healthy lifestyle skills?		None found at the time of mapping
D.3 Workforce	D.3.1 Are there training and resources available for health care professionals to support families?		Free, online training is available for health and community professionals in WA, provides training on how to have a non-judgemental and supportive conversation with parents about children's weight. *Talking with parents about children's weight* (Link) sessions are delivered by the Better Health Company (independent health provider).
	- D.3.1a Is preconception advice for nutrition and being active provided to prospective parents?		None found at the time of mapping
	D.3.2 Is there a state/territory health promotion…<br>- D.3.2a …agency (independent or adjunct to health department)?		The Chronic Disease Prevention Directorate (Link) is responsible for health promotion in WA. The *WA Health Promotion Strategic Framework 2017-2021* (Link) sets out the strategic 5 year plan for health promotion in WA. It identifies target groups (people experiencing lower socio-economic conditions, disability, newly arrived migrants from non-English speaking backgrounds, and people who identify as Aboriginal).<br><br>*Population monitoring*: Continuous data collection (550 households per month) via CATI survey – all ages (*Western Australia Health and Wellbeing Surveillance System* (Link) annual collection and Nutrition Monitoring Survey every 3 years. In 2018, 599 children aged 0-15years were sampled, there was limited reporting on BMI and physical activity/sedentary behaviour for children under five years in the 2018 report (Link).
	- D.3.2b …workforce (to implement initiatives locally)?		The Chronic Disease Prevention Directorate is a discrete workforce within the Department of Health. The *Public Health Act 2016* requires the Chief Health Officer to develop a *Public Health Plan*, and then each local government is required to develop *Local Public Health Plans*. Local councils are to receive support from their associated Health Service (e.g. SMHS Health Promotion, Link) to develop and implement their local public health plans.
